# Geospatial and modelling analyses reveal diverse tick and tick-associated microbes in the East African Community

**DOI:** 10.1186/s40249-025-01310-y

**Published:** 2025-05-22

**Authors:** Abakundana Nsenga Ariston Gabriel, Xiao-Yang Wang, Guo-Yao Zu, Pei-Yu Zhen, Laila Jamil, Shi-Jing Shen, Cheng Li, Ntakirutimana Theoneste, Lin Zhao, Wu-Chun Cao

**Affiliations:** 1 Institute of EcoHealth, School of Public Health, Cheeloo College of Medicine, Shandong University, Jinan, 250012 Shandong People’s Republic of China; 2 State Key Laboratory of Pathogen and Biosecurity, Beijing Institute of Microbiology and Epidemiology, Beijing, 100071 People’s Republic of China; 3 School of Public Health, College of Medicine and Health Sciences, University of Rwanda, Kigali, Rwanda

**Keywords:** Tick, Tick-associated microbes, East African Community, Geographical distribution, Ecological niche model

## Abstract

**Background:**

The continuous geographic expansion of ticks and the emergence of tick-borne diseases have raised tremendous global public health concerns, particularly in the East African Community (EAC). This study aimed to investigate the distribution of ticks and tick-associated microbes and to predict the potential extension of dominant tick species in the EAC.

**Methods:**

Data were collected from literature reviews and related websites and analyzed using ArcGIS to generate maps showing the geographical distribution of ticks and associated microbes. Meta-analyses were conducted to estimate the positive rates of microbes. Ecological niche modelling was used to project the potential expansion of predominant tick species.

**Results:**

A total of 138 tick species were recorded in the seven EAC countries, including five genera of the Argasidae family, eight of the Ixodidae family, and monospecific Nuttalliellidae. Overall, 64 tick-associated microbes, including 22 viruses, 26 bacteria, and 16 protists, were identified, of which 43 (11 viruses, 21 bacteria, and 11 protists) were pathogenic to humans or animals. Among them, 5 (2 viruses and 3 bacteria) have been reported in humans, while 10 pathogens (1 virus, 4 bacteria, and 5 protists) have been reported in animals. The predictive model identified suitable habitats for four dominant tick species, with certain species flourishing under ideal conditions, such as elevation, temperature, and vegetation. Our study revealed that ticks might affect broader areas where they have never been previously reported.

**Conclusions:**

Ticks are widely prevalent in the EAC, and some ticks harbor a variety of microbial agents that can have significant pathogenetic implications for human and animal health. Therefore, EAC authorities and medical personnel should acknowledge the potential threat posed by ticks and tick-associated pathogens to the well-being of people and animals. Surveillance and etiological diagnosis should be enhanced to control ticks and prevent tick-borne infections.

**Supplementary Information:**

The online version contains supplementary material available at 10.1186/s40249-025-01310-y.

## Background

Tick populations are continuously increasing globally, partly because of climatic change and socioeconomic developments, resulting in a quick geographical expansion [1, 2]. Known to feed on animals and sometimes on humans, ticks are among the most important vectors for people and animals [3]. Each tick species favours particular biotopes or eco-environments, which determine its geographical range and related risk areas of tick-borne infections in humans and animals. Ticks can transmit viral, bacterial, and parasitic diseases; a single tick may transmit more than one pathogen [4]. The prevalence and transmission of endemic and emerging tick-borne diseases have steadily risen, posing a growing public health problem worldwide [5–9].

The East African Community (EAC), consisting of Tanzania, Kenya, Uganda, Rwanda, the Democratic Republic of the Congo (DR Congo), Burundi, and South Sudan, has reported several tick-borne diseases, including but not limited to East Coast fever (ECF), Crimean-Congo hemorrhagic fever (CCHF), and African tick-bite fever [10–12]. Furthermore, the economic losses in Tanzania due to tick-borne animal diseases were estimated at 364 million US dollars, primarily because of cattle mortality caused by *Theileria*, *Anaplasma,* and *Babes*ia [13]. Despite extensive efforts to investigate tick-borne infections, they have been neglected by health authorities, primary healthcare providers, and the public. Additionally, the lack of adequate national tick monitoring programs to track the spread of tick populations has consequently led to a poor understanding of the substantial public health threats caused by ticks and tick-borne diseases in the EAC region. The zoonotic nature of tick-borne diseases needs a thorough knowledge of their transmission patterns, frequently poorly described due to weak surveillance and monitoring efforts.

Thus, this study aims to address these gaps by compiling data on different tick species from multiple sources, conducting a comprehensive investigation on the geographical distribution of ticks and their associated microbes, predicting suitable areas for ticks, and providing valuable data to support effective prevention and control of tick and tick-borne infections.

## Methods

### Data extraction and management

Data on ticks and tick-associated microbes in the seven EAC countries were collected from various sources, including literature reviews and reputable websites. Three independent reviewers (Gabriel ANA, Wang X–Y, and Zu G-Y) searched electronic databases Web of Science, PubMed, Scopus, and Embase for papers published from January 1, 1968, to December 31, 2022, using the terms “ticks”, “tick-borne diseases”, “tick-borne zoonosis”, “tick-borne zoonotic disease”, “tick-associated agent”, and “tick-associated microbe”, and this was done by adding each country's name or by adding East African Community. The detailed search strategy for each database is provided in Text S1. While acknowledging the significance of incorporating literature in Kiswahili, most publications in this field were in English; consequently, full articles were included if they were only published in English and provided information on tick collection locations. Articles lacking adequate information or duplicates were excluded. Additional information on the literature search, data extraction, and references used in the meta-analysis can be found in Fig. S1 and Text S2. We also searched the websites of GenBank (https://www.ncbi.nlm.nih.gov/genbank/), the Walter Reed Biosystematics Unit Vectormap (WRBU Vectormap, https://vectormap.si.edu/), and the Global Biodiversity Information Facility (GBIF, https://www.gbif.org/), to collect related information. The dataset was compiled by merging data from the websites and published literature. Duplicate entries were removed, and the refined database was subsequently utilized.

The data of 19 bioclimatic variables were downloaded from WorldClim (www.worldclim.org). Slope, aspect, and elevation variables were derived from WorldClim (www.worldclim.org) by ArcGIS using the Spatial Analyst Tool. Vegetation data (Percent Tree Cover) and land cover information (GLCNMO) were obtained from the Resource and Environmental Science and Data Center of Global Map (https://globalmaps.github.io/). These variables, each with a spatial resolution of 10 km, were employed to develop a predictive model for the potential distribution of ticks; the variables used in the analysis were measured on either a monthly or an annual basis; more details are provided in Table S1.

### Mapping of ticks and associated microbes

Each tick collection site's latitude and longitude coordinates were used for mapping. When exact locations were unavailable, the centroids of administrative areas were used for mapping purposes. Thematic maps depicting the geographical distribution of ticks and tick-associated microbes were generated using ArcGIS software (version 10.6; ESRI, Redlands, CA, USA).

### Meta-analysis

A meta-analysis was performed to estimate the combined positive rate and 95% confidence interval (*CI*) for each tick-associated microbe using R software (version 4.0.5; The R Development Core Team, R Foundation for Statistical Computing, Vienna, Austria) meta package. The *I*^2^ statistic was used to evaluate the heterogeneity of the data. A random effects model was applied if the heterogeneity was significant (*I*^2^ > 50%). Otherwise, a common effect model was used. If only one study was included for a microbe, the positive rate was determined by dividing the number of positive ticks by the total number of ticks without considering a 95% *CI*.

### Modelling prediction of dominant tick potential distribution

An ecological niche model (ENM) was used to predict EAC's potential distributions of four dominant tick species: *Amblyomma variegatum Haemaphysalis leachi*, *Hyalomma truncatum*, and *Rhipicephalus appendiculatus*. The model excluded location data associated with the centroids of administrative regions. Ultimately, 3143 unique geographic data points were retained to study the four tick species. The model parameters were set using the R software with the Kuenm package, and ENM was executed in Maxent software (version 3.4.1; American Museum of Natural History, New York, USA) [14]. A detailed description of the analysis process is provided in Text S3.

## Results

### Characteristics of tick and tick-associated microbes database

**Fig. 1:**
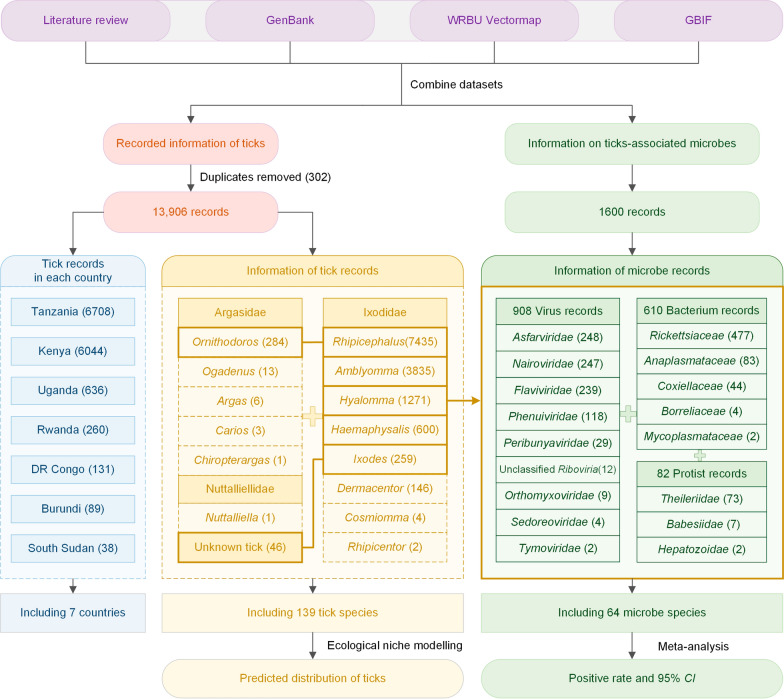
Study design and data sources. *WRBU Vectormap* the Walter Reed Biosystematics Unit Vectormap, *GBIF* Global Biodiversity Information Facility, *CI* Confidence interval. The tick genus within the thick outlines were identified to carry the microbes

After removing 302 duplicates, the analysis of multi-source data revealed 13,906 records of ticks, of which 421 were from published papers, 1522 from GenBank, 11,708 from WRBU Vectormap, and 255 from GBIF, including 6708 in Tanzania, 6044 in Kenya, 636 in Uganda, 260 in Rwanda, 131 in Democratic Republic of the Congo (DRC), 89 in Burundi and 38 in South Sudan. These records represented a diverse array of 138 tick species from 5 genera of the Argasidae family, 8 genera of the Ixodidae family, and the monospecific Nuttalliellidae (Table S2). Among 1600 microbe records, 908 were associated with viruses, 610 with bacteria, and 82 with protists (Fig. 1).

### Geographical distribution of ticks

**Fig. 2:**
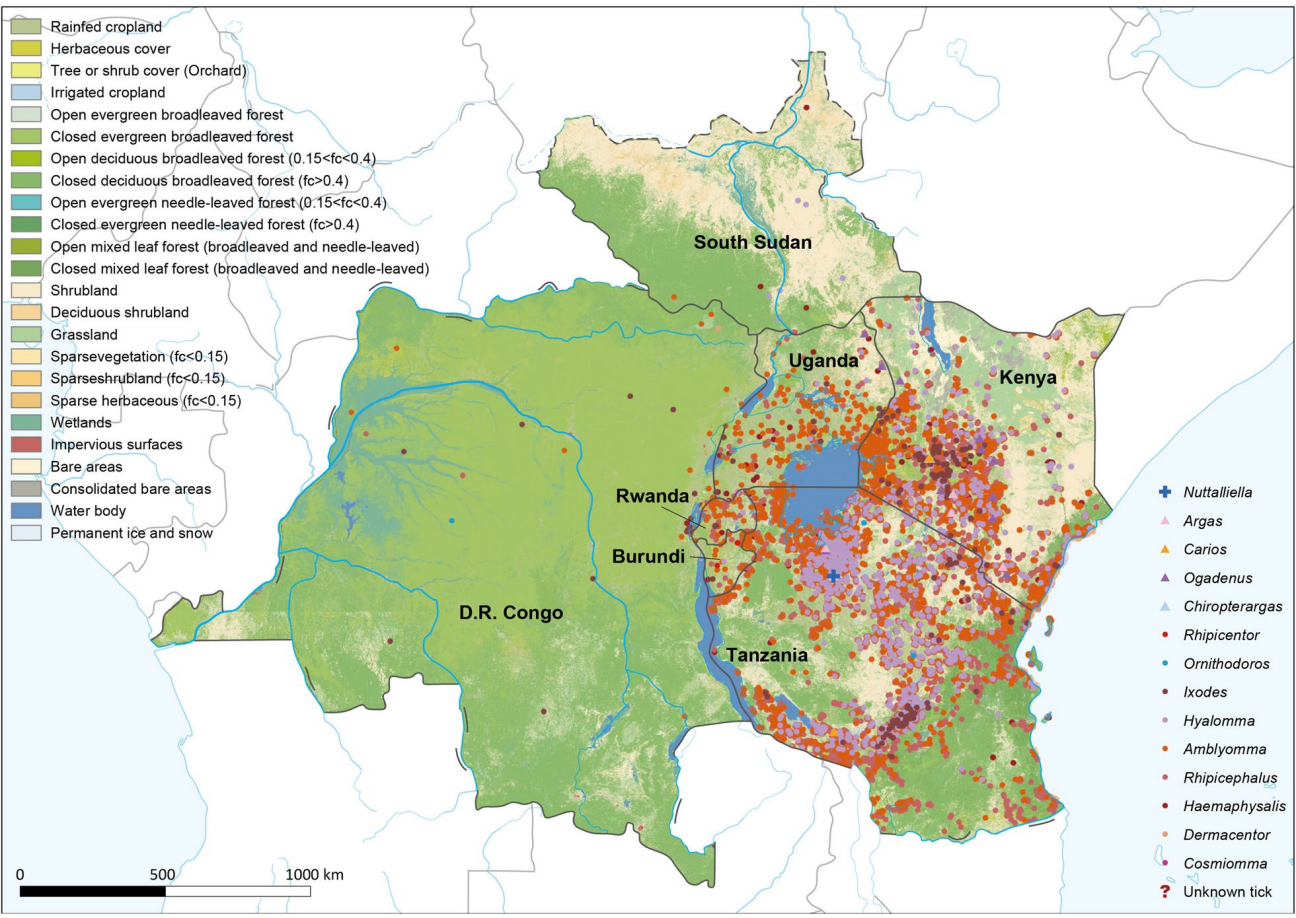
Geographical distribution maps of ticks in East African Community. The base map is landcover. Shows recorded locations of each tick genus*.* Map approval No.: GS(2025)1426

As shown in Fig. 2, despite the variations in the geographical distribution of different tick genera, regardless of their genera, they were more abundant in areas with evergreen broadleaved forests and near water bodies. The most commonly encountered ticks belonged to the genus *Rhipicephalus*, which encompassed 45 species with 7299 records and distributed in the widest ranges of all seven EAC countries. *Amblyomma* was the second most abundant genus, with 3461 records of 23 species, and it showed a similar spatial distribution to *Rhipicephalus* ticks. The genera *Hyalomma* and *Haemaphysalis* had 1186 records of 8 species and 588 records of 14 species, respectively, both of which displayed various distributions. Notably, the *Ixodes* genus had fewer records (255) but more diverse species (31) than the above three tick genera. The geographical distribution maps of the remaining tick species are presented in Fig.S2. Moreover, tick distributions across the seven EAC countries revealed that ticks were more prevalent in regions with high altitudes and high population count (Fig.S3 and S4).

### Hosts of ticks in East African Community

Besides a few free-living ticks, the overwhelming majority of ticks in EAC countries were collected from a variety of animal families with microbe-associated ticks, including Bovidae (cows), Camelidae (camels), Canidae (dogs), Felidae (cats), Suidae (pigs), which were widely distributed in Kenya, Tanzania, and Uganda (Fig. S5). No records of animal hosts with microbe-associated ticks were reported in Rwanda, Burundi, DR Congo, and South Sudan (Fig. S5). A total of 125 species of animals could serve as hosts of 106 tick species, mainly in the genera *Rhipicephalus*, *Amblyomma*, *Hyalomma*, and *Haemophysalis* (Fig. S6).

### Presence and prevalence of tick-associated microbe

**Fig. 3:**
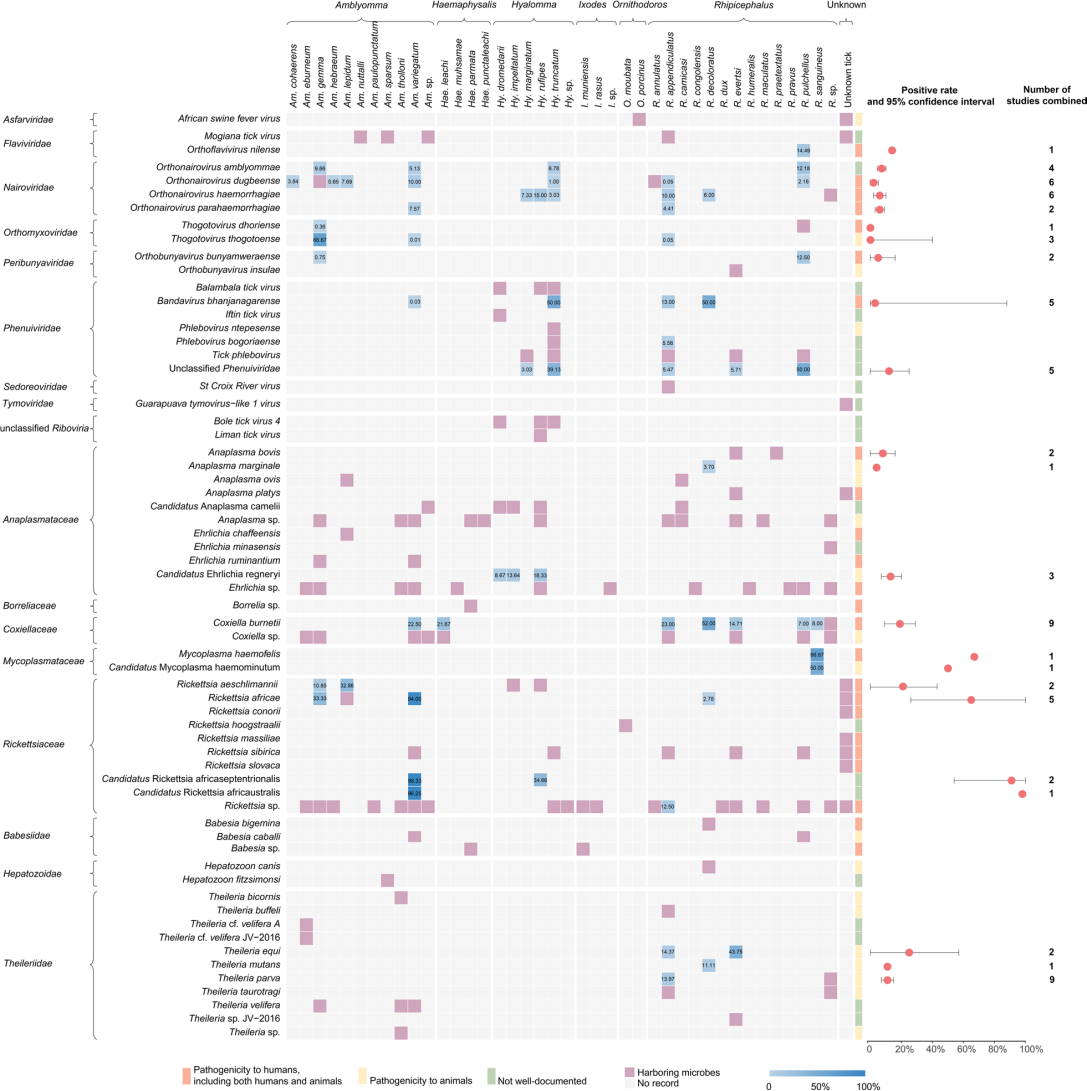
Presence and prevalence of each tick-associated microbe in East African Community. In the Gantt chart, the violet grid indicates the presence of each tick-associated microbe, which is unable to calculate the positive rate. The blue grid indicates the positive rate for each microbe, with the color intensity and the number representing the value of the positive rate (percent scale). The merged positive rate and 95% confidence interval (*CI*) for each microbe in multiple tick species are provided at the end of each row

A total of 40 tick species were identified as carriers of 64 microbial species, including14 from the genus *Rhipicephalus*, 11 *Amblyomma*, six *Hyalomma*, four *Haemaphysalis*, three Ixodes, and two *Ornithodoros*. The microbial diversity consisted of 22 viral species belonging to nine families, 26 bacterial species from five families, and 16 protist species from three families (Fig. 3). Except for 10 tick species, most species carried two or more microbes. *R. appendiculatus* was found to carry the highest number of microbes, with 19 species, including 10 viruses, five bacteria, and four protists. Furthermore, *Amblyomma variegatum* ranked second, harboring 17 microbes (5 viruses, 10 bacteria, and 2 protists). Notably, the more prevalent a tick species was, the more tick-associated microbes were identified (Fig. S7). On the other hand, several tick-associated microbes were observed to infect multiple tick species. For instance, *Orthonairovirus dugbeense* was discovered in 9 tick species, *Coxiella burnetii* in 8 tick species, and *Orthonairovirus haemorrhagiae* (the causative agent of CCHFV) in 6 tick species. Additionally, many unclassified *Rickettsia*, *Ehrlichia*, and *Anaplasma* species were each identified in over 11 tick species. Among the 64 recorded microbes’ species, 24 were classified as human pathogens (including those simultaneously pathogenic to both humans and animals), 19 were identified as animal pathogens (exclusively pathogenic to animals), whereas 21 were categorized as microbes with unknown pathogenicity (Fig. 3).

The meta-analysis was conducted on the tick-associated microbes, for which information on the number of tick samples tested and the number of positive samples was available. As a result, 11 viruses, 10 bacteria, and 3 protists were included to estimate their positive rates, which are displayed as blue grids in Fig. 3. The estimation of the positive rate for each microbe in a tick species is provided in Table S3 and Fig. S8. Four viruses across the *Orthonairovirus* genus were each identified in two or more tick species. *Orthonairovirus amblyommae* was identified in four tick species, with an overall positive rate of 8.47% (*I*^*2*^ = 77%, 95% *CI* 5.35–11.60). *Orthonairovirus haemorrhagiae*, the pathogen of CCHFV, was detected in *Hy. marginatum*, *Hy. rufipes*, *Hy. truncatum*, *R. appendiculatus*, *R. decoloratus*, and *R*. sp., with an overall estimated positive rate of 6.35% (*I*^*2*^ = 82%, 95% *CI* 2.50–10.20). The other three viral pathogens, i.e. *Orthoflavivirus nilense*, *Orthobunyavirus bunyamweraense* and *Bandavirus bhanjanagarense*, infected various tick species, each of which had an estimated positive rate of 14.49%, 4.71% (*I*^*2*^ = 67%, 95% *CI* 0.00–15.60), and 2.91% (*I*^*2*^ = 97%, 95% *CI* 0.10–87.90), respectively. Among the tick-associated bacteria, rickettsiae showed high infection rates. *Candidatus Rickettsia africaustralis* exhibited a highest pooled positive rate of 96.25%, followed by *Candidatus Rickettsia africaseptentrionalis* (*I*^*2*^ = 100%, 66.55%, 95% *CI* 4.15–100.00), and *Rickettsia africae* (*I*^*2*^ = 100%, 65.10%, 95% *CI* 26.16–100.00), which has been known to cause African tick-bite fever [15]. *Rickettsia aeschlimannii*, another known human pathogen [16], had a pooled positive rate of 21.31% (*I*^*2*^ = 92%, 95% *CI* 0.00–42.85). *C. burnetii*, the causative agent of Q fever [17], had a pooled positive rate of 19.27% (*I*^*2*^ = 89%, 95% *CI* 9.22–29.33). *Anaplasma bovis* had a pooled positive rate of 7.63% (*I*^*2*^ = 0%, 95% *CI* 0.00–16.49), which had been reported as the causative agent of human and animal infections [18]. *Candidatus Ehrlichia regneryi*, which is pathogenic to animals, had a prevalence rate of 13.33% (*I*^*2*^ = 81%, 95% *CI* 6.83–19.84). Besides, in the Theridiidae family, *Theileria equi* had a high pooled positive rate of 24.67% (*I*^*2*^ = 86%, 95% *CI* 0.00–57.42), while *T. mutans* and *T. parva* had pooled positive rates of 11.11% and 9.90% (*I*^*2*^ = 97%, 95% *CI* 6.01–13.78), respectively. The forest figure of combined positive rates for each microbe in all positive tick species can be found in Fig. S9.

### Geographical distribution of tick-associated microbes

**Fig. 4:**
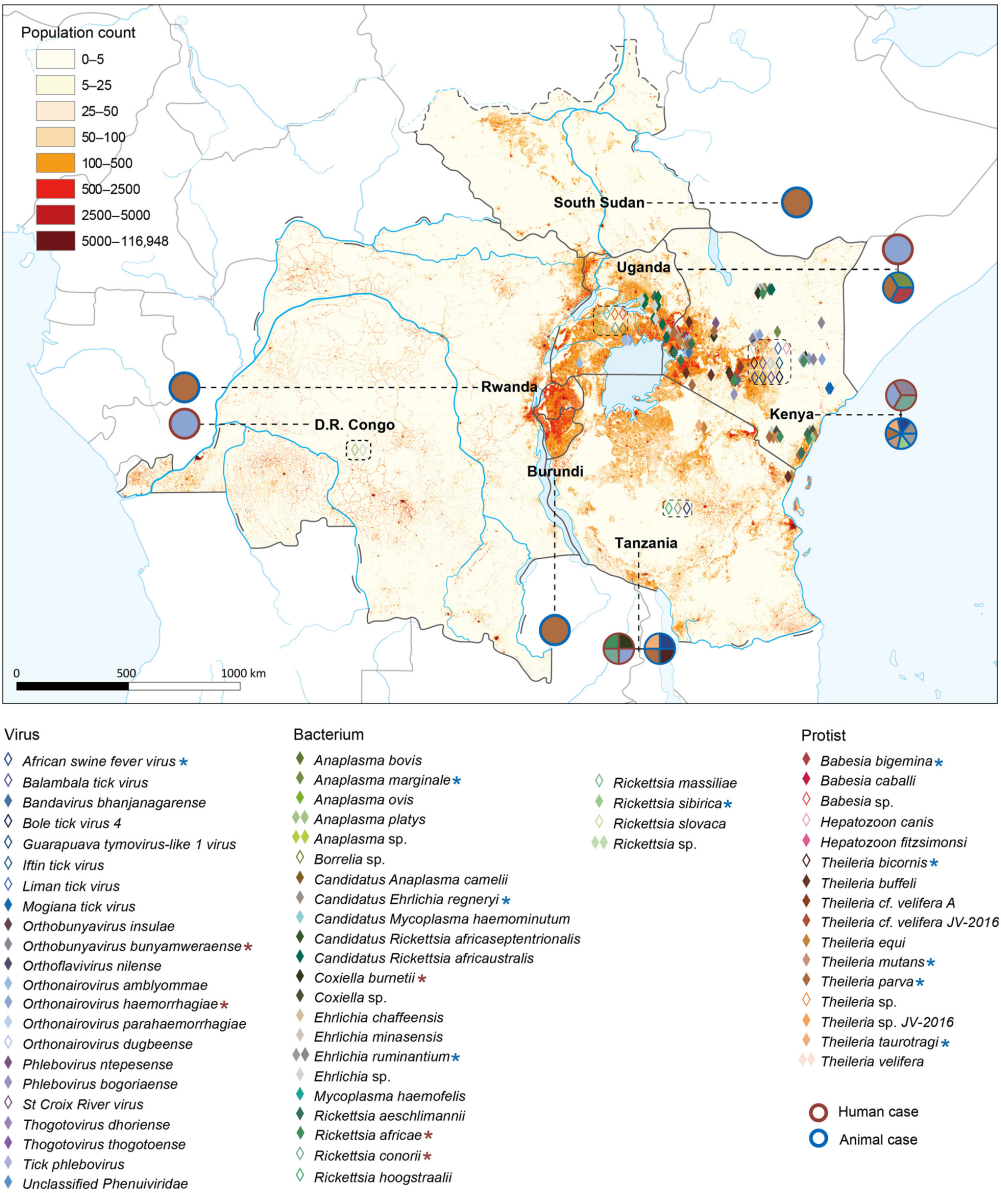
Geographical distribution of tick-associated microbes in East African Community. The base map is density of human population. The solid diamond represents a microbe with known location. A hollow diamond indicates the presence of the microbe in the country but without a specific location; thus, it is marked at the center of the country and enclosed with a dashed box. A pie with the dark red circle outline and “*” indicates pathogens that infect humans, while a pie with a dark blue circle outline and “*” indicates pathogens that infect animals. Each color within the diamonds and pies corresponds to a specific microbe, as shown in the legend. Map approval No.: GS(2025)1426

We mapped the geographical distribution of tick-associated microbes carried by various tick species (Fig. 4). Although ticks were distributed across all seven EAC countries, tick-associated microbes were only reported in ticks from Kenya, Uganda, Tanzania, and DR Congo, with no report in the remaining three countries. Most (89.63%) tick-associated microbes were reported in Kenya, where 21 species of viruses, 5 species of spotted fever group *rickettsiae*, 11 species within the family *Anaplasmataceae,* 8 *Theileria* species, and 2 *Babesia* species were detected (Fig. 4). Fourteen tick-associated microbes were identified in ticks from Uganda, including two viruses, 10 bacteria and two protists (Fig. 4). One virus, 5 bacteria, and 3 protists were detected in ticks from two regions of Tanzania (Fig. 4). Only two bacteria (*Anaplasma platys* and *Ehrlichia* sp.) were detected in DR Congo. Patient cases of CCHF caused by *Orthonairovirus haemorrhagiae* were reported in Kenya, Tanzania, Uganda, and the DR Congo [19–23]. *Orthobunyavirus bunyamweraense* and *Rickettsia conorii* were identified in febrile patients from Kenya [24, 25]. Patients infected with *R. africae*, *Rickettsia conorii*, and *Coxiella burnetii* infections were reported in Tanzania [26, 27]. Livestock disease ECF caused by *T. parva* has been reported in six EAC countries, excluding the DR Congo [28–33]. Additionally, animal infections with African swine fever virus, *Rickettsia sibirica*, *Ehrlichia ruminantium*, *Candidatus Ehrlichia regneryi*, *Anaplasma marginale*, *Theileria bicornis*, *T. mutans*, *Theileria taurotragi*, and *Babesia bigemina* were recorded in Kenya, Tanzania, and Uganda [34–41].

### Predicted suitable habitats of four dominant tick species

**Fig. 5:**
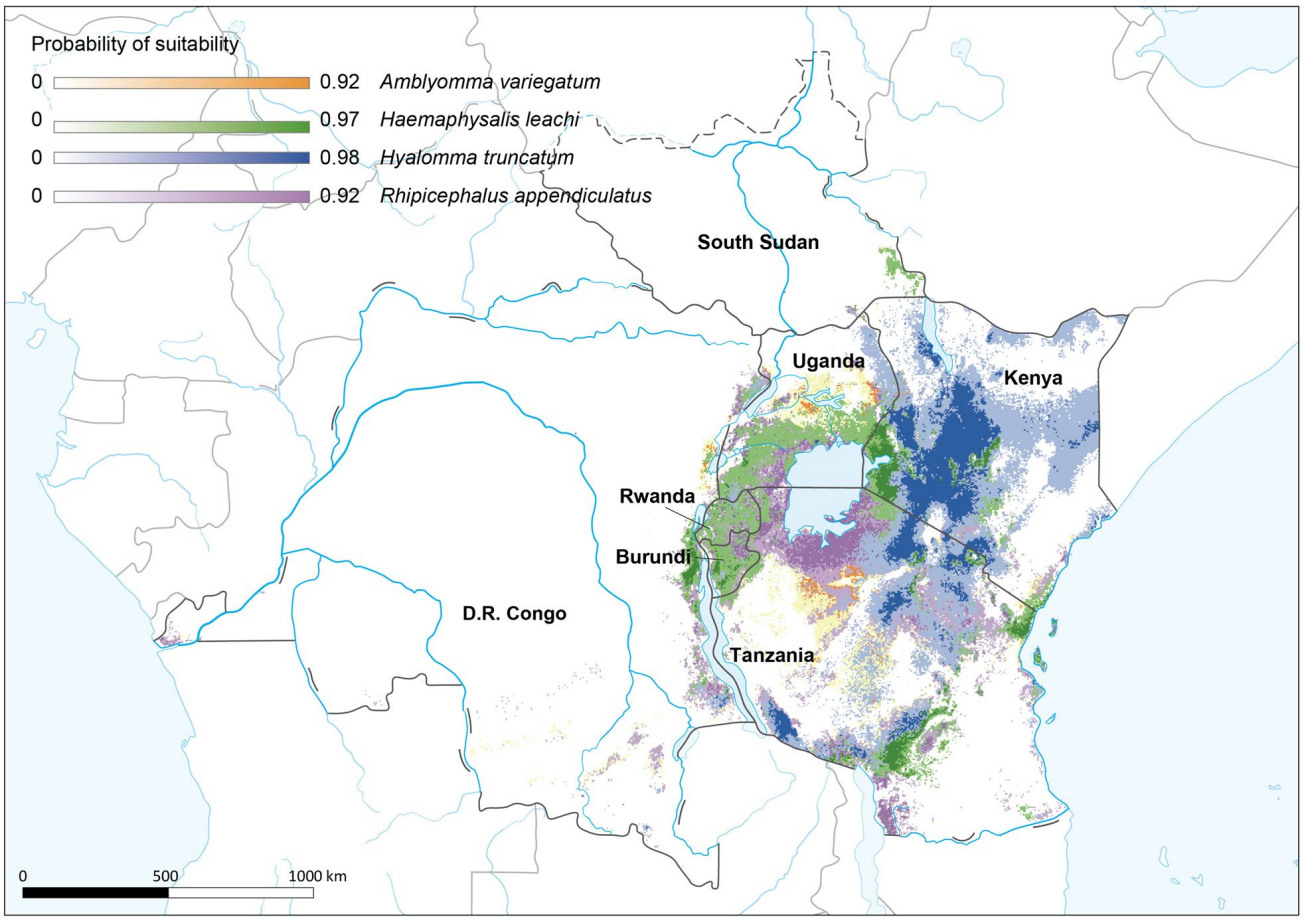
Potential distribution map of four dominant ticks’ species in East African Community. The predicted probabilities of suitability for each tick species are presented as continuous values ranging from 0 to 1, as generated by Maxent. The predicted areas suitable for *Amblyomma variegatum, Haemaphysalis leachi, Hyalomma truncatum* and *Rhipicephalus appendiculatus.* Map approval No.: GS(2025)1426

We predicted areas suitable for four dominant ticks species by ecological niche modelling. The model for *Am. variegatum* included nine independent variables and produced an average AUC value of 0.854 ± 0.0155 (Table S4, Fig. S10), indicating a respectable level of predictive ability. The modelling revealed that elevation and mean temperature of the coldest quarter were the primary variables influencing the geographical distribution of *Am. variegatum* ticks, contributing over 71.80% to the model (Fig. 5, Fig. S11). Furthermore, when including all the variables in the model, the predicted response curves showed that low and high mountainous areas were suitable habitats. Additionally, the most suitable areas for *Am*. *variegatum* was the northwest of Uganda, the north of Tanzania, and some eastern parts of DR. Congo (Fig. 5, Fig. S12).

On the other hand, the prediction model for *Hae. leachi* included thirteen independent variables with an optimization AUC score of 0.907 ± 0.028 (Fig. S13), showing excellent prediction accuracy. The distribution of *Hae. leachi* ticks were mainly influenced by elevation, the mean temperature of the wettest quarter, the mean temperature of the coldest quarter, and the precipitation of the driest quarter (Table S5), accounting for 82.50% of the model’s distribution (Fig. 5, Fig. S14, S15). Kenya was identified as the most suitable site for *Hae. leachi*, particularly in its southwestern region. Some areas in the southern part of Tanzania were also found suitable sites for *Hae. leachi*. Twelve environmental variables were included in the model to predict *Hy. truncatum* distribution, achieving an AUC value of 0.888 ± 0.045 (Fig. S16). The four major variables, including annual precipitation, annual mean temperature, percentage of tree cover, and elevation, explained 75.30% of the model (Fig. 5, Table S6, and Fig. S17, S18). Furthermore, the southwestern part of Kenya and the northern part of Tanzania were identified as the most suitable habitat for *Hy. truncatum*. Ten variables were included in the prediction model for *R. appendiculatus* ticks, obtaining an AUC value of 0.867 ± 0.014 (Fig. S19). The distribution of *R. appendiculatus* was mainly influenced by elevation, mean temperature of the coldest quarter, tree cover percentage, and the precipitation of the driest month, explaining 83.40% of the model (Fig. 5, Table S7, Fig. S20, S21). Suitable habitats for *R. appendiculatus* were identified in Kenya as well as in the northern and southern parts of Tanzania. Most areas of Burundi,Rwanda and Uganda also exhibited suitable conditions for the distribution of *R. appendiculatus*.

## Discussion

COVID-19 outbreaks have drawn significant attention to emerging infectious diseases. However, emerging tick-borne infections have been neglected worldwide, particularly in Africa. We mapped the distribution of ticks and tick-associated microbes in EAC and revealed that 138 tick species have been identified. These included 127 species belonging to 8 genera of the Ixodidae (so-called hard tick) family, 10 species across 5 genera of the Argasidae (soft tick) family, and a single species of the monospecific Nuttalliellidae family. They can transmit tens of pathogens when feeding on a range of animals.

As ectothermic, ticks are profoundly influenced by environmental factors, climate, weather, and biotopes that affect their reproduction, survival, geographic distribution, and capacity to transmit pathogens [1, 41]. The predictive models identified the most suitable habitats for different tick species and predicted their potential presence in previously unrecorded areas. This underscores the risk posed by ticks and tick-borne pathogens to humans and livestock, emphasizing the need for medical and veterinary attention.

The integrated data from EAC countries reveal that the ticks in this region are highly diverse, comprising 138 species across all known genera. Moreover, the density in certain areas, especially in Kenya, is exceptionally high. These ticks are mainly distributed from 10 °N to 10 °S close to the equator. Considering that suitable environmental conditions and the availability of appropriate hosts are crucial to ticks’ presence, this broad geographic distribution in EAC suggests ticks are tolerant to extreme temperatures [42]. This may also be due to their range of animal hosts, which includes 43 animal families. The most commonly recorded ticks in the genera of *Rhipicephalus*, *Amblyomma*, and *Hyalomma* usually feed on median or large-sized mammalian hosts such as those in Bovidae and Camelidae families, as does *Ixodes ricinus* in Europe [43]. These animals primarily graze on grasslands, which might account for the widespread presence of these ticks in this region.

Notably, out of the 64 tick-associated recorded microbes in EAC, 26 are recognized human pathogens, highlighting the substantial threat to public health. However, only a few patients with tick-borne diseases have been reported in the EAC countries. This is likely because of the nonspecific febrile symptoms caused by tick-borne pathogens and the lack of accessible laboratory tests, which makes diagnosis challenging. For instance, *R. africae*, the causative agent of African tick-bite fever, is a frequently reported tick-borne pathogen in Africa. However, the disease has been underreported due to nonspecific clinical symptoms with human infection [44]. The high prevalence of tick-associated pathogens and their vectors implies that the reported human cases are only a small fraction of the actual burden. Unfortunately, the threats of tick-borne diseases to human and animal health are often overlooked due to the unavailability of diagnostic etiological tests and inadequate surveillance in medical facilities. There is an urgent need to develop convenient and practical laboratory diagnostic tests for these pathogens to assess the burden of tick-borne infections.

Furthermore, our pooled data revealed that pathogens, such as CCHFV and *C. burnetii*, can infect more tick species, indicating that more possible transmitting vectors exist in this region. For example, ticks in the genus *Hyalomma* are typically considered the primary vectors for CCHFV. However, a cross-sectional and modelling study discovered that *Rhipicephalus,* rather than *Hyalomma* ticks, plays a vital role in the transmission of the virus in Uganda [45], supported by the broad distributions and substantial CCHFV infections of *R. appendiculatus* and *R. decoloratus* across the country [46, 47]. Integrated, landscape-level eco-epidemiological studies are needed to inform surveillance and control efforts for the region's CCHF and other tick-borne diseases. Despite these findings, some countries like Rwanda, Burundi, and South Sudan have never reported tick-associated microbes in ticks, which may be attributed to the lack of investigations. This suggests a need to conduct further studies.

Our prediction modelling in our study indicated that the four dominant tick species, also potential vectors for multiple pathogens in the EAC, may occupy significantly larger areas than previously recorded, thereby increasing the probability of transmitting more tick-borne infections to humans and animals. Various climatic, ecological, and environmental factors influence this expansion and abundance of the different tick species [1, 48].

The distribution of *Am. variegatum* was mainly influenced by elevation and the mean temperature during the coldest quarter. These findings align with the study on *Amblyomma americanum*, which similarly highlighted the role of elevation and mean temperature of the coldest quarter in determining the distribution of this species in North America [49, 50]. Similarly, higher elevations and an increased mean temperature during the wettest quarter correlate with a greater likelihood of suitability for *Hae. leachi*. These findings are consistent with the study's results on the ecology and management of ixodid ticks in Zimbabwe, which identified elevation and wettest quarter temperature as crucial factors influencing the distribution of *Hae. Leachi* [51]. *Hy. Truncatum* ticks are more prevalent in areas with high annual precipitation and tree cover, which aligns with findings from Cameron on distribution, seasonal dynamics, and *Hy. Truncatum’s* suitability [52]. For *R. appendiculatus* ticks, the model indicates that its distribution is predominantly influenced by elevation and the mean temperature of the coldest quarter. These climatic factors are crucial for predicting the distribution of ticks and understanding their ecological dynamics, suggesting that the effective management of tick populations and control of tick-borne infections require a detailed understanding of these influencing factors.

Although our study reveals essential results, it has some limitations. Firstly, only literature published in English was included, potentially excluding pertinent reports in other languages. Secondly, microbe-positive rates may vary owing to differences in test methodologies, reagents, and sensitivities. Finally, unquantifiable elements such as human activities were not examined, which may have influenced the accuracy of the prediction.

## Conclusions

Ticks in EAC countries pose significant threats to public health due to their high availability and capacity to feed on various hosts and carry a variety of tick-associated microbes that may cause diseases in humans and animals. Our predictive models suggest that ticks might present in more extensive areas. An effective tick monitoring system involving all the EAC members is needed to control ticks and tick-borne infections. Practical cooperation across EAC governments and all their respective health institutions is critical to ensuring the well-being of people and animals via improved data exchange and coordinated, evidence-based decision-making.

## Supplementary Information

Supplementary material 1.

## Data Availability

Data are available on reasonable request. The data can be accessed by contacting caowuchun@126.com or zhaolin1989@sdu.edu.cn.

## Abbreviations

EAC: East African Community
DR Congo: Democratic Republic of the Congo
ECF: East Coast fever
CCHF: Crimean-Congo hemorrhagic fever
WRBU Vectormap: The Walter Reed Biosystematics Unit Vectormap
GBIF: Global Biodiversity Information Facility
ENM: Ecological niche model
CCHFV: Crimean-Congo hemorrhagic fever virus
*CI*: Confidence interval
