# Supplementary material for: Geospatial and modelling analyses reveal diverse tick and tick-associated microbes in the East African Community
Source: Infect Dis Poverty. 2025 May 22;14:39. doi: 10.1186/s40249-025-01310-y (PMC12096497; doi:10.1186/s40249-025-01310-y)

**Supplemental material**

# **Contents**

[Text S1. The detailed search strategy for each database 1](#_Toc197537586)

[Fig. S1. PRISMA flow diagram of study selection process 2](#_Toc197537587)

[Text S2. References of ticks and tick-associated microbes in the East African Community 3](#_Toc197537588)

[Table S1. Environmental and meteorological factors for prediction of ticks distribution 10](#_Toc197537589)

[Text S3. Detailed method for predicting the potential distribution of ticks 13](#_Toc197537590)

[Table S2. Ticks identified in the East African Community 14](#_Toc197537591)

[Fig. S2. Geographic distribution maps of remaining ticks in the East African Community 19](#_Toc197537592)

[Fig. S3. Geographic distribution maps of ticks in the East African Community based on altitude 20](#_Toc197537593)

[Fig. S4. Geographic distribution maps of ticks in the East African Community based on population count 21](#_Toc197537594)

[Fig. S5. Sankey diagram of hosts, tick species and microbes in East African Community 22](#_Toc197537595)

[Fig. S6. Matrix of tick species and hosts in the East African Community 23](#_Toc197537596)

[Fig. S7. Correlation analysis of tick species and tick-associated microbes 24](#_Toc197537597)

[Table S3. The estimated positive rate and 95% confidence interval (CI) for each microbe in a tick species 25](#_Toc197537598)

[Fig. S8. Meta-analysis of positive rate of each tick species-associated microbe in a tick species 32](#_Toc197537599)

[Fig. S9. The forest Fig. of combined positive rates for each microbe in all positive tick species 34](#_Toc197537600)

[Table S4. Relative contributions of environmental and meteorological variables to the MaxEnt model 39](#_Toc197537601)

[Fig. S10. Receiver Operating Characteristic (ROC) curve of the best MCP model for *Amblyomma variegatum* 40](#_Toc197537602)

[Fig. S11. Jackknife plots of MaxEnt model for *Amblyomma variegatum* prediction. 41](#_Toc197537603)

[Fig. S12. Response curves of environmental variables indicate the likelihood of *Amblyomma variegatum* being present. 42](#_Toc197537604)

[Fig. S13. Receiver Operating Characteristic (ROC) curve of the best MCP model for *Haemaphysalis leachi* 43](#_Toc197537605)

[Table S5. Relative contributions of environmental and meteorological variables to the MaxEnt model 44](#_Toc197537606)

[Fig. S14. Jackknife plots of MaxEnt model for *Haemaphysalis leachi* prediction. 45](#_Toc197537607)

[Fig. S15. Response curves of environmental variables indicate the likelihood of *Haemaphysalis leachi* being present. 46](#_Toc197537608)

[Fig. S16. Receiver Operating Characteristic (ROC) curve of the best MCP model for *Hyalomma truncatum* 48](#_Toc197537609)

[Table S6. Relative contributions of environmental and meteorological variables to the MaxEnt model 49](#_Toc197537610)

[Fig. S17. Jackknife plots of MaxEnt model for *Hyalomma truncatum* prediction. 50](#_Toc197537611)

[Fig. S18. Response curves of environmental variables indicate the likelihood of *Hyalomma truncatum* being present. 51](#_Toc197537612)

[Fig. S19. Receiver Operating Characteristic (ROC) curve of the best MCP model for *Rhipicephalus appendiculatus* 53](#_Toc197537613)

[Table S7. Relative contributions of environmental and meteorological variables to the MaxEnt model 54](#_Toc197537614)

[Fig. S20. Jackknife plots of MaxEnt model for *Rhipicephalus appendiculatus* prediction. 55](#_Toc197537615)

[Fig. S21. Response curves of environmental variables indicate the likelihood of *Rhipicephalus appendiculatus* being present. 56](#_Toc197537616)

# **Text S1. The detailed search strategy for each database**

**PubMed:**

((Tick[Title/Abstract]) OR (tick[Title/Abstract]) OR ([Ixodida](https://www.ncbi.nlm.nih.gov/Taxonomy/Browser/wwwtax.cgi?mode=Info&id=6935&lvl=3&p=has_linkout&p=blast_url&p=genome_blast&lin=f&keep=1&srchmode=1&unlock)[Title/Abstract]) OR (tick borne disease[Title/Abstract]) OR (tick borne illnesses[Title/Abstract])) AND (([Burundi](https://en.wikipedia.org/wiki/Burundi)[Title/Abstract]) OR (Democratic Republic of the Congo[Title/Abstract]) OR (DRC[Title/Abstract]) OR ([DR Congo](https://en.wikipedia.org/wiki/Democratic_Republic_of_the_Congo)[Title/Abstract]) OR (Kenya[Title/Abstract]) OR (Rwanda[Title/Abstract]) OR (South Sudan[Title/Abstract]) OR (Tanzania[Title/Abstract]) OR (Uganda[Title/Abstract]) OR (East Africa[Title/Abstract]) OR (Eastern Africa[Title/Abstract]) OR (East of Africa[Title/Abstract]) OR ([East African Community](https://en.wikipedia.org/wiki/East_African_Community)[Title/Abstract]))

**WOS:**

(TS=(Tick) OR TS=(tick) OR TS=(Ixodida) OR TS=(tick borne disease) OR TS=(tick borne illnesses)) AND (TS=([Burundi](https://en.wikipedia.org/wiki/Burundi)) OR TS=(Democratic Republic of the Congo) OR TS=(DRC) OR TS=([DR Congo](https://en.wikipedia.org/wiki/Democratic_Republic_of_the_Congo)) OR TS=(Kenya) OR TS=(Rwanda) OR TS=(South Sudan) OR TS=(Tanzania) OR TS=(Uganda) OR TS=(East Africa) OR TS=(Eastern Africa) OR TS=(East of Africa) OR TS=([East African Community](https://en.wikipedia.org/wiki/East_African_Community)))

**Scopus[Title/Abstract/Keywords]:**

((Tick) OR (tick) OR ([Ixodida](https://www.ncbi.nlm.nih.gov/Taxonomy/Browser/wwwtax.cgi?mode=Info&id=6935&lvl=3&p=has_linkout&p=blast_url&p=genome_blast&lin=f&keep=1&srchmode=1&unlock)) OR (tick-borne disease) OR (tick-borne illnesses) OR (tick-borne zoonotic disease) OR (tick-borne zoonotic) OR (tick-associated agent) OR (tick-associated microbe)) AND (([Burundi](https://en.wikipedia.org/wiki/Burundi)) OR (Democratic Republic of the Congo) OR (DRC) OR ([DR Congo](https://en.wikipedia.org/wiki/Democratic_Republic_of_the_Congo)) OR (Kenya) OR (Rwanda) OR (South Sudan) OR (Tanzania) OR (Uganda) OR (East Africa) OR (Eastern Africa) OR (East of Africa) OR ([East African Community](https://en.wikipedia.org/wiki/East_African_Community)))

**Embase:**

(Tick:ti,ab,kw OR tick:ti,ab,kw OR [Ixodida](https://www.ncbi.nlm.nih.gov/Taxonomy/Browser/wwwtax.cgi?mode=Info&id=6935&lvl=3&p=has_linkout&p=blast_url&p=genome_blast&lin=f&keep=1&srchmode=1&unlock):ti,ab,kw OR tick-borne disease:ti,ab,kw OR tick-borne illnesses:ti,ab,kw OR tick-borne zoonotic disease:ti,ab,kw OR tick-borne zoonotic:ti,ab,kw OR tick-associated agent:ti,ab,kw OR tick-associated microbe:ti,ab,kw) AND ([Burundi](https://en.wikipedia.org/wiki/Burundi):ti,ab,kw OR Democratic Republic of the Congo:ti,ab,kw OR DRC:ti,ab,kw OR [DR Congo](https://en.wikipedia.org/wiki/Democratic_Republic_of_the_Congo):ti,ab,kw OR Kenya:ti,ab,kw OR Rwanda:ti,ab,kw OR South Sudan:ti,ab,kw OR Tanzania:ti,ab,kw OR Uganda:ti,ab,kw OR East Africa:ti,ab,kw OR Eastern Africa:ti,ab,kw OR East of Africa:ti,ab,kw OR [East African Community](https://en.wikipedia.org/wiki/East_African_Community):ti,ab,kw)

# **Fig. S1. PRISMA flow diagram of study selection process**


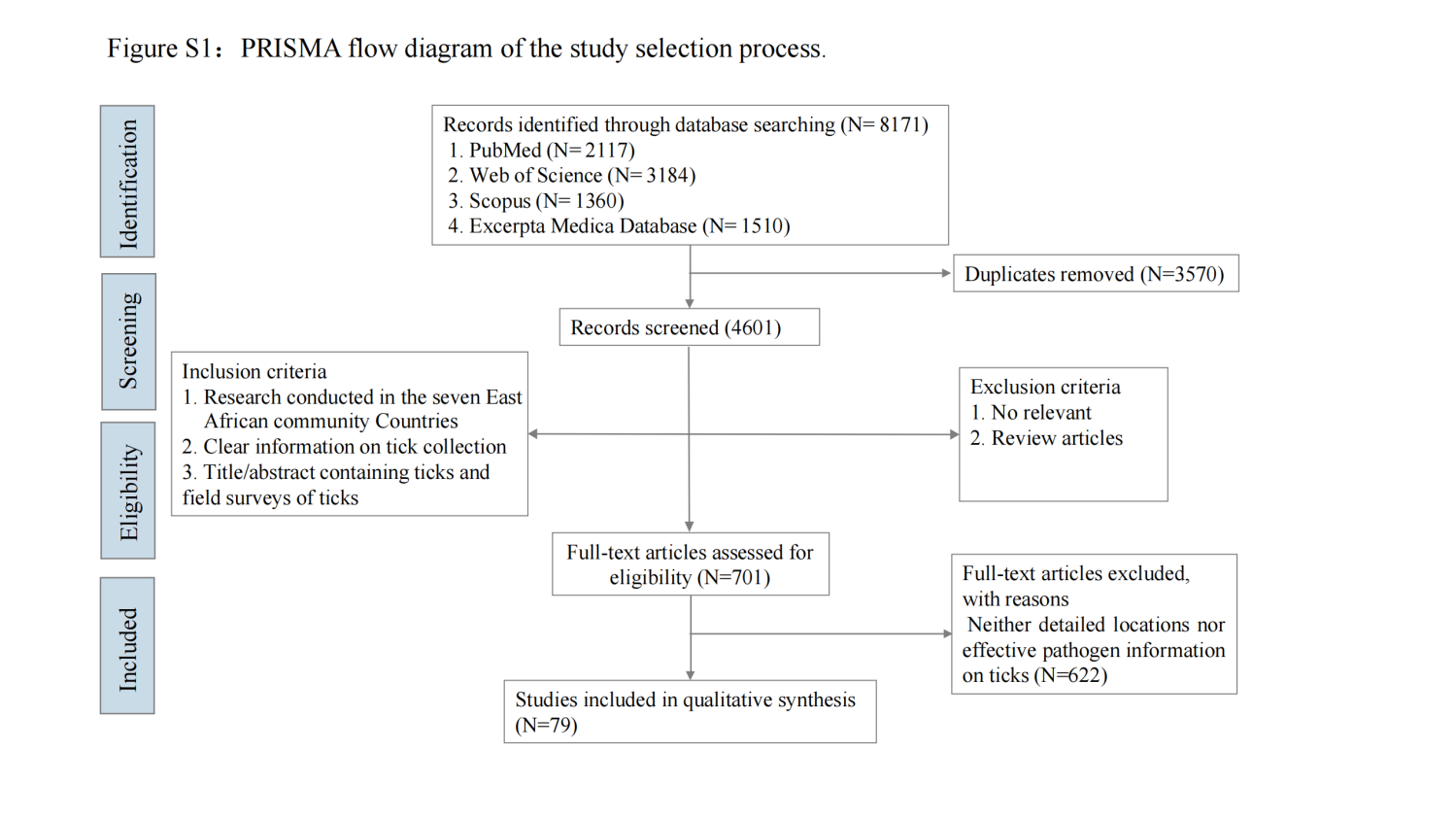


# **Text S2. References of ticks and tick-associated microbes in the East African Community**

The following information has been extracted from each eligible publication: first author, publication year, locations of collection sites, number of tick samples tested, and number of samples positive for a specific pathogen.

1. Dupont HT, La Scola B, Williams R, Raoult D: A focus of tick-borne relapsing fever in southern Zaire. *Clin Infect Dis* 1997, 25(1):139-144.

2. Swai ES, French NP, Beauchamp G, Fitzpatrick JL, Bryant MJ, Kambarage D, Ogden NH: A longitudinal study of sero-conversion to tick-borne pathogens in smallholder dairy youngstock in Tanzania. *Vet Parasitol* 2005, 131(1-2):129-137.

3. Horak IG, Apanaskevich DA, Kariuki EK: A new species of Rhipicephalus (Acari: Ixodidae), a parasite of giraffes in Kenya. *J Med Entomol* 2013, 50(4):685-690.

4. Apanaskevich DA, Horak IG, Mulumba-Mfumu LK: A new species of Rhipicephalus (Acari: Ixodidae), a parasite of red river hogs and domestic pigs in the Democratic Republic of Congo. *J Med Entomol* 2013, 50(3):479-484.

5. Zulu FP, Okello-Onen J, Punyua D, Essuman S, Malonza MM: A Note on the Ticks of Domestic Animals in Coast Province, Kenya. *International Journal of Tropical Insect Science* 1998, 18(2):163-165.

6. Irvin AD, Brown CG, Burridge MJ, Cunningham MP, Musoke AJ, Pierce MA, Purnell RE, Radley DE: A pathogenic theilerial syndrome of cattle in the Narok District of Kenya. I. Transmission studies. *Trop Anim Health Prod* 1972, 4(4):220-229.

7. Watt D, Kiara H, Sparagano OA: A PCR-based field evaluation of Theileria infections in cattle and ticks in Kenya. *Ann N Y Acad Sci* 1998, 849:69-77.

8. Crampton PL, Gichanga MM: A survey of resistance to acaricides in economically important Ixodidae (Acari) of the major cattle-raising areas of Kenya. *Bulletin of Entomological Research* 1979, 69(3):427-439.

9. Warwick BT, Bak E, Baldassarre J, Gregg E, Martinez R, Kioko J, Saning’o K, Kiffner C: Abundance estimations of ixodid ticks on Boran cattle and Somali sheep in Northern Tanzania. *International Journal of Acarology* 2016, 42(1):12-17.

10. Matthysse JG, Colbo MH, Kamya E: Acaricide trials against Rhipicephalus appendiculatus Neum., Amblyomma variegatum (F.) and Boophilus decoloratus (Koch)(Ixodidae) on cattle in Uganda. *Bulletin of Entomological Research* 1969, 58(3):465-486.

11. Plowright W, Parker J, Peirce M: African swine fever virus in ticks (Ornithodoros moubata, Murray) collected from animal burrows in Tanzania. *Nature* 1969, 221(5185):1071-1073.

12. Nakao R, Qiu Y, Igarashi M, Magona JW, Zhou L, Ito K, Sugimoto C: High prevalence of spotted fever group rickettsiae in Amblyomma variegatum from Uganda and their identification using sizes of intergenic spacers. *Ticks and tick-borne diseases* 2013, 4(6):506-512.

13. D’Amico G, Dumitrache MO, Široký P, Albrechtová K, Sloboda M, Domşa C, Sándor AD, Balázsi R, Kanyari PW, Modrý D: Altitudinal and seasonal differences of tick communities in dogs from pastoralist tribes of Northern Kenya. *Veterinary Parasitology* 2015, 212(3-4):318-323.

14. Moran M, Nigarura G, Pegram R: An assessment of host resistance to ticks on cross‐bred cattle in Burundi. *Medical and Veterinary Entomology* 1996, 10(1):12-18.

15. Wesonga F, Kitala P, Gathuma J, Njenga M, Ngumi P: An assessment of tick-borne diseases constraints to livestock production in a smallholder livestock production system in Machakos District, Kenya. 2010.

16. Latif A, Rowlands G, Punyua D, Hassan S, Capstick P: An epidemiological study of tick-borne diseases and their effects on productivity of zebu cattle under traditional management on Rusinga Island, western Kenya. *Preventive Veterinary Medicine* 1995, 22(3):169-181.

17. Bazarusanga T, Geysen D, Vercruysse J, Madder M: An update on the ecological distribution of Ixodid ticks infesting cattle in Rwanda: countrywide cross-sectional survey in the wet and the dry season. *Experimental and Applied Acarology* 2007, 43:279-291.

18. Sang R, Onyango C, Gachoya J, Mabinda E, Konongoi S, Ofula V, Dunster L, Okoth F, Coldren R, Tesh R: Tickborne arbovirus surveillance in market livestock, Nairobi, Kenya. *Emerging infectious diseases* 2006, 12(7):1074.

19. Johnson B, Chanas A, Squires E, Shockley P, Simpson D, Parsons J, Smith D, Casals J: Arbovirus isolations from ixodid ticks infesting livestock, Kano Plain, Kenya. *Transactions of the Royal Society of Tropical Medicine and Hygiene* 1980, 74(6):732-737.

20. Lynen G, Zeman P, Bakuname C, Di Giulio G, Mtui P, Sanka P, Jongejan F: Cattle ticks of the genera Rhipicephalus and Amblyomma of economic importance in Tanzania: distribution assessed with GIS based on an extensive field survey. *Experimental and Applied Acarology* 2007, 43:303-319.

21. Wesonga F, Orinda G, Ngae G, Grootenhuis J: Comparative tick counts on game, cattle and sheep on a working game ranch in Kenya. *Tropical Animal Health and Production* 2006, 38:35-42.

22. Byaruhanga J, Odua F, Ssebunya Y, Aketch O, Tayebwa DS, Rwego IB, Vudriko P: Comparison of tick control and antibiotic use practices at farm level in regions of high and low acaricide resistance in Uganda. *Veterinary Medicine International* 2020, 2020.

23. Maingi N, Njoroge G: Constraints on production, disease perceptions and ticks and helminths control practices on dairy cattle farms in Nyandarua District, Kenya. *Livestock Research for Rural Development* 2010, 22(8):138.

24. Muberuka J: La lutte contre les tiques au Rwanda: Situation actuelle. *International Journal of Tropical Insect Science* 1992, 13:621-628.

25. Juvenal N, Edward M: Seasonal dynamics and distribution of ticks in Rwanda: implications for tick control strategy in Rwanda. *Journal of Animal and Veterinary Advances* 2010, 2(1):21-25.

26. Okello-Onen J, Heinonen R, Ssekitto C, Mwayi W, Kakaire D, Kabarema M: Control of tsetse flies in Uganda by dipping cattle in deltamethrin. *Tropical Animal Health and Production* 1994, 26(1):21-27.

27. Koka H, Sang R, Kutima HL, Musila L: Coxiella burnetii detected in tick samples from pastoral communities in Kenya. *BioMed Research International* 2018, 2018.

28. Knobel DL, Maina AN, Cutler SJ, Ogola E, Feikin DR, Junghae M, Halliday JE, Richards AL, Breiman RF, Cleaveland S: Coxiella burnetii in humans, domestic ruminants, and ticks in rural western Kenya. *The American journal of tropical medicine and hygiene* 2013, 88(3):513.

29. Nyakarahuka L, Whitmer S, Kyondo J, Mulei S, Cossaboom CM, Telford CT, Tumusiime A, Akurut GG, Namanya D, Kamugisha K: Crimean-Congo hemorrhagic fever outbreak in refugee settlement during COVID-19 pandemic, Uganda, April 2021. *Emerging Infectious Diseases* 2022, 28(11):2326.

30. Sang R, Lutomiah J, Koka H, Makio A, Chepkorir E, Ochieng C, Yalwala S, Mutisya J, Musila L, Richardson JH: Crimean-Congo hemorrhagic fever virus in Hyalommid ticks, northeastern Kenya. *Emerging infectious diseases* 2011, 17(8):1502.

31. Msami H, Khaschabi D, Schöpf K, Kapaga A, Shibahara T: Dermatophilus congolensis infection in goats in Tanzania. *Tropical Animal Health and Production* 2001, 33:367-377.

32. Young A, Leitch B, Morzaria S, Irvin A, Omwoyo P, De Castro J: Development and survival of Theileria parva parva in Rhipicephalus appendiculatus exposed in the Trans-Mara, Kenya. *Parasitology* 1987, 94(3):433-441.

33. Gitau G, McDermott JJ, Katende J, O'callaghan C, Brown R, Perry BD: Differences in the epidemiology of theileriosis on smallholder dairy farms in contrasting agro-ecological and grazing strata of highland Kenya. *Epidemiology & Infection* 2000, 124(2):325-335.

34. Fyumagwa RD, Runyoro V, Horak IG, Hoare R: Ecology and control of ticks as disease vectors in wildlife of the Ngorongoro Crater, Tanzania. *South African Journal of Wildlife Research-24-month delayed open access* 2007, 37(1):79-90.

35. Kaiser M, Sutherst R, Bourne A, Gorissen L, Floyd R: Population dynamics of ticks on Ankole cattle in five ecological zones in Burundi and strategies for their control. *Preventive Veterinary Medicine* 1988, 6(3):199-222.

36. Wampande EM, Waiswa P, Allen DJ, Hewson R, Frost SD, Stubbs SC: Phylogenetic characterization of Crimean-Congo hemorrhagic fever virus detected in African blue ticks feeding on cattle in a Ugandan abattoir. *Microorganisms* 2021, 9(2):438.

37. Cutler SJ, Browning P, Scott JC: Ornithodoros moubata, a soft tick vector for Rickettsia in east Africa? *Annals of the New York Academy of Sciences* 2006, 1078(1):373-377.

38. Mamiro KA, Magwisha HB, Rukambile EJ, Ruheta MR, Kimboka EJ, Malulu DJ, Malele II: Occurrence of ticks in cattle in the new pastoral farming areas in Rufiji district, Tanzania. *Journal of Veterinary Medicine* 2016, 2016.

39. Keesing F, Allan BF, Young TP, Ostfeld RS: Effects of wildlife and cattle on tick abundance in central Kenya. *Ecological applications* 2013, 23(6):1410-1418.

40. De Castro J, Young A, Dransfield R, Cunningham M, Dolan T: Effects of tick infestation on Boran (Bos indicus) cattle immunised against theileriosis in an endemic area of Kenya. *Research in veterinary science* 1985, 39(3):279-288.

41. Vudriko P, Okwee-Acai J, Tayebwa DS, Byaruhanga J, Kakooza S, Wampande E, Omara R, Muhindo JB, Tweyongyere R, Owiny DO: Emergence of multi-acaricide resistant Rhipicephalus ticks and its implication on chemical tick control in Uganda. *Parasites & vectors* 2016, 9:1-13.

42. Byaruhanga C, Collins N, Knobel D, Kabasa W, Oosthuizen M: Endemic status of tick-borne infections and tick species diversity among transhumant zebu cattle in Karamoja Region, Uganda: Support for control approaches. *Veterinary Parasitology: Regional Studies and Reports* 2015, 1:21-30.

43. Lule SA, Gibb R, Kizito D, Gladys N, Mutyaba J, Balinandi S, Owen L, Jones KE, Abubakar I, Lutwaama JJ *et al*: Widespread exposure to Crimean-Congo haemorrhagic fever in Uganda might be driven by transmission from Rhipicephalus ticks: evidence from cross-sectional and modelling studies. *The Journal of infection* 2022.

44. Etiang P, Musoba A, Nalumenya D, Ndekezi C, Bbira J, Ochwo S, Tweyongyere R, Muhanguzi D: Distribution and prevalence of ixodid tick species (Acari: Ixodidae) infesting cattle from North-eastern Uganda [Karamoja]; 2019-2022. In*.*: Research Square; 2023.

45. Ochanda H, Young AS, Medley GF, Perry BD: Vector competence of 7 rhipicephalid tick stocks in transmitting 2 Theileria parva parasite stocks from Kenya and Zimbabwe. *Parasitology* 1998, 116 ( Pt 6):539-545.

46. Nyabongo L, Odongo DO, Milton G, Machuka E, Vudriko P, Pelle R, Kanduma EG: Molecular survey of cattle ticks in Burundi: First report on the presence of the invasive Rhipicephalus microplus tick. *PLoS One* 2021, 16(12):e0261218.

47. Nyoap SSM, Majok AA, Salih DA: A survey of ticks and East Coast fever among cattle in Fangak County, Jonglei State, South Sudan. In*: 2015*; 2015.

48. Njanja JC, Rinkanya FGF, Kiara HK: Ticks of camels, sheep and goats in northwestern Kenya rangelands. *Tropical Pest Management* 1991, 37(2):166-168.

49. Marcellino WL, Julla, II, Salih DA, El Hussein AR: Ticks infesting cattle in Central Equatoria region of South Sudan. *Onderstepoort J Vet Res* 2011, 78(1):336.

50. Lutomiah J, Musila L, Makio A, Ochieng C, Koka H, Chepkorir E, Mutisya J, Mulwa F, Khamadi S, Miller BR *et al*: Ticks and tick-borne viruses from livestock hosts in arid and semiarid regions of the eastern and northeastern parts of Kenya. *J Med Entomol* 2014, 51(1):269-277.

51. Getange D, Bargul JL, Kanduma E, Collins M, Bodha B, Denge D, Chiuya T, Githaka N, Younan M, Fèvre EM *et al*: Ticks and Tick-Borne Pathogens Associated with Dromedary Camels (Camelus dromedarius) in Northern Kenya. *Microorganisms* 2021, 9(7).

52. Kariuki EK, Penzhorn BL, Horak IG: Ticks (Acari: Ixodidae) infesting cattle and African buffaloes in the Tsavo conservation area, Kenya. *Onderstepoort J Vet Res* 2012, 79(1):E1-4.

53. Wanzala W, Okanga S: Ticks (Acari: Ixodidae) associated with wildlife and vegetation of Haller park along the Kenyan coastline. *J Med Entomol* 2006, 43(5):789-794.

54. Chiuya T, Masiga DK, Falzon LC, Bastos ADS, Fèvre EM, Villinger J: Tick-borne pathogens, including Crimean-Congo haemorrhagic fever virus, at livestock markets and slaughterhouses in western Kenya. *Transbound Emerg Dis* 2021, 68(4):2429-2445.

55. Kivaria FM, Kapaga AM, Mbassa GK, Mtui PF, Wani RJ: Epidemiological perspectives of ticks and tick-borne diseases in South Sudan: cross-sectional survey results. *Onderstepoort J Vet Res* 2012, 79(1):E1-e10.

56. Haji I, Simuunza M, Kerario, II, Jiang N, Chen Q: Epidemiology of tick-borne pathogens of cattle and tick control practices among mixed farming and pastoral communities in Gairo and Monduli districts, Tanzania. *Vet Parasitol Reg Stud Reports* 2022, 32:100738.

57. Ogden NH, Gwakisa P, Swai E, French NP, Fitzpatrick J, Kambarage D, Bryant M: Evaluation of PCR to detect Theileria parva in field-collected tick and bovine samples in Tanzania. *Vet Parasitol* 2003, 112(3):177-183.

58. Rinkanya FGR, Tatchell RJ: Evaluation of the efficacy of different pour‐on formulations against cattle ticks in Kenya 1. *Tropical Pest Management* 1988, 34(3):324-327.

59. Swai ES, Karimuribo ED, Rugaimukamu EA, Kambarage DM: Factors influencing the distribution of questing ticks and the prevalence estimation of T. parva infection in brown ear ticks in the Tanga region, Tanzania. *J Vector Ecol* 2006, 31(2):224-228.

60. Swai E S MAN, Kessy V, Kaaya E, Sanka P and Loomu P M Farm constraints, cattle disease perception and tick management practices in pastoral Maasai community-Ngorongoro, Tanzania. *Livestock Research for Rural Development* 2005, 2(9):20-30.

61. Price JE, Karstad LH: Free-living jackals (Canis mesomelas)-potential reservoir hosts for Ehrlichia canis in Kenya. *J Wildl Dis* 1980, 16(4):469-473.

62. Balinandi S, Mugisha L, Bbira J, Kabasa W, Nakayiki T, Bakkes DK, Lutwama JJ, Chitimia-Dobler L, Malmberg M: General and Local Morphological Anomalies in Amblyomma lepidum (Acari: Ixodidae) and Rhipicephalus decoloratus Infesting Cattle in Uganda. *Journal of Medical Entomology* 2019, 56(3):873-877.

63. Lwande OW, Venter M, Lutomiah J, Michuki G, Rumberia C, Gakuya F, Obanda V, Tigoi C, Odhiambo C, Nindo F *et al*: Whole genome phylogenetic investigation of a West Nile virus strain isolated from a tick sampled from livestock in north eastern Kenya. *Parasit Vectors* 2014, 7:542.

64. Vudriko P, Umemiya-Shirafuji R, Okwee-Acai J, Tayebwa DS, Byaruhanga J, Jirapattharasate C, Liu M, Adjou Moumouni PF, Fujisaki K, Xuan X *et al*: Genetic mutations in sodium channel domain II and carboxylesterase genes associated with phenotypic resistance against synthetic pyrethroids by Rhipicephalus (Boophilus) decoloratus ticks in Uganda. *Pestic Biochem Physiol* 2017, 143:181-190.

65. Maina AN, Jiang J, Omulo SA, Cutler SJ, Ade F, Ogola E, Feikin DR, Njenga MK, Cleaveland S, Mpoke S *et al*: High prevalence of Rickettsia africae variants in Amblyomma variegatum ticks from domestic mammals in rural western Kenya: implications for human health. *Vector Borne Zoonotic Dis* 2014, 14(10):693-702.

66. Kalume MK, Saegerman C, Mbahikyavolo DK, Makumyaviri AM, Marcotty T, Madder M, Caron Y, Lempereur L, Losson B: Identification of hard ticks (Acari: Ixodidae) and seroprevalence to Theileria parva in cattle raised in North Kivu Province, Democratic Republic of Congo. *Parasitol Res* 2013, 112(2):789-797.

67. Proboste T, Kalema-Zikusoka G, Altet L, Solano-Gallego L, Fernández de Mera IG, Chirife AD, Muro J, Bach E, Piazza A, Cevidanes A *et al*: Infection and exposure to vector-borne pathogens in rural dogs and their ticks, Uganda. *Parasit Vectors* 2015, 8:306.

68. Davies FG: Karai virus, a probable arbovirus isolated from sheep and from the tick Rhipicephalus evertsi in Kenya. *J Comp Pathol* 1982, 92(1):9-14.

69. Rothen J, Githaka N, Kanduma EG, Olds C, Pflüger V, Mwaura S, Bishop RP, Daubenberger C: Matrix-assisted laser desorption/ionization time of flight mass spectrometry for comprehensive indexing of East African ixodid tick species. *Parasit Vectors* 2016, 9:151.

70. Bazarusanga T, Geysen D, Vercruysse J, Madder M: An update on the ecological distribution of Ixodid ticks infesting cattle in Rwanda: countrywide cross-sectional survey in the wet and the dry season. *Exp Appl Acarol* 2007, 43(4):279-291.

71. Juvénal N, Edward M: Seasonal dynamics and distribution of ticks in Rwanda: implications for tick control strategy in Rwanda. In*: 2010*; 2010.

72. Corrigan J, Marion B, English J, Eneku W, Weng JL, Rugg M, Dotrang T, Dunford J, Byaruhanga AM, Byarugaba DK *et al*: Minimal Rickettsial Infection Rates and Distribution of Ticks in Uganda: An Assessment of the Seasonal Effects and Relevance to Tick-Borne Disease Risk in East Africa. *J Med Entomol* 2023, 60(1):185-192.

73. Fyumagwa R, Simmler P, Meli ML, Hoare RE, Hofmann-Lehmann R, Lutz H: Prevalence of Anaplasma marginale in different tick species from Ngorongoro Crater, Tanzania. *Veterinary parasitology* 2009, 161 1-2:154-157.

74. Vudriko P, Umemiya-Shirafuji R, Okwee-Acai J, Tayebwa DS, Byaruhanga JK, Jirapattharasate C, Liu M, Adjou Moumouni PF, Fujisaki K, Xuan X *et al*: Genetic mutations in sodium channel domain II and carboxylesterase genes associated with phenotypic resistance against synthetic pyrethroids by Rhipicephalus (Boophilus) decoloratus ticks in Uganda. *Pesticide biochemistry and physiology* 2017, 143:181-190.

75. Adrien N, Maurice BM, Annick L, Juvénal N: Preliminary Study on Ixodid Ticks Population of the Akagera National Park in Rwanda. In*: 2013*; 2013.

76. Vector-Borne Diseases, Surveillance, Prevention Minimal Rickettsial Infection Rates and Distribution of Ticks in Uganda: An Assessment of the Seasonal Effects and Relevance to Tick-Borne Disease Risk in East Africa. In*: 2022*; 2022.

77. Fyumagwa R, Simmler P, Meli ML, Hoare RE, Hofmann-Lehmann R, Lutz H: Molecular detection of and species in a diversity of tick species from Ngorongoro Crater, Tanzania. In*: 2011*; 2011.

78. Fyumagwa R, Simmler P, Willi B, Meli ML, Sutter A, Hoare RE, Dasen G, Hofmann-Lehmann R, Lutz H: Molecular detection of haemotropic Mycoplasma species in Rhipicephalus sanguineus tick species collected on lions (Panithera leo) from Ngorongoro Crator, Tanzania. In*: 2008*; 2008.

79. Byamukama B, Vudriko P, Tumwebaze MA, Tayebwa DS, Byaruhanga JK, Angwe MK, Li J, Galon EMS, Ringo AE, Liu M *et al*: Molecular detection of selected tick-borne pathogens infecting cattle at the wildlife-livestock interface of Queen Elizabeth National Park in Kasese District, Uganda. *Ticks and tick-borne diseases* 2021, 12 5:101772.

# **Table S1. Environmental and meteorological factors for prediction of ticks distribution**

| Environment variable | Description | Source |
| --- | --- | --- |
| BIO1 | Annual Mean Temperature | WorldClim database  ([www.worldclim.org](http://www.worldclim.org)) |
| BIO2 | Mean Diurnal Range  (Mean of monthly (max temp - min temp)) |  |
| BIO3 | Isothermality (BIO2/BIO7) (×100) |  |
| BIO4 | Temperature Seasonality (standard deviation ×100) |  |
| BIO5 | Max Temperature of Warmest Month |  |
| BIO6 | Min Temperature of Coldest Month |  |
| BIO7 | Temperature Annual Range (BIO5-BIO6) |  |
| BIO8 | Mean Temperature of Wettest Quarter |  |
| BIO9 | Mean Temperature of Driest Quarter |  |
| BIO10 | Mean Temperature of Warmest Quarter |  |
| BIO11 | Mean Temperature of Coldest Quarter |  |
| BIO12 | Annual Precipitation |  |
| BIO13 | Precipitation of Wettest Month |  |
| BIO14 | Precipitation of Driest Month |  |
| BIO15 | Precipitation Seasonality (Coefficient of Variation) |  |
| BIO16 | Precipitation of Wettest Quarter |  |
| BIO17 | Precipitation of Driest Quarter |  |
| BIO18 | Precipitation of Warmest Quarter |  |
| BIO19 | Precipitation of Coldest Quarter |  |
| Slope | Slope | Extracted from theWorldClim database ([www.worldclim.org](http://www.worldclim.org)) by ArcGIS" |
| Aspect | Aspect |  |
| Elevation | Elevation |  |
| Land Cover | Land Cover | Resource Environmental Sciences and Global Map Data  (https://globalmaps.github.io/) |
| Percent Tree Cover | Percent Tree Cover |  |

Environmental and meteorological factors for prediction

Legend for Land Cover type

| Code | Class Name |
| --- | --- |
| 1 | Broadleaf Evergreen Forest |
| 2 | Broadleaf Deciduous Forest |
| 3 | Needleleaf Evergreen Forest |
| 4 | Needleleaf Deciduous Forest |
| 5 | Mixed Forest |
| 6 | Tree Open |
| 7 | Shrub |
| 8 | Herbaceous |
| 9 | Herbaceous with Sparse Tree/Shrub |
| 10 | Sparse vegetation |
| 11 | Cropland |
| 12 | Paddy field |
| 13 | Cropland/Other Vegetation Mosaic |
| 14 | Mangrove |
| 15 | Wetland |
| 16 | Bare area, consolidated (gravel, rock) |
| 17 | Bare area, unconsolidated (sand) |
| 18 | Urban |
| 19 | Snow/lce |
| 20 | Water bodies |

# **Text S3. Detailed method for predicting the potential distribution of ticks**

We used the ENMTools program (version 1.4.4) to remove duplicate entries and occurrences within 10 km of each other ^1^. To identify the optimal combination of variables, we conducted an initial modeling analysis using Maxent software (version 3.4.1). The jackknife test was used to identify the key environmental variables that influence tick distribution. Response curves were used to show the association between distribution probabilities and environmental conditions. The model was tested using a random subsampling technique, where 75% of the dataset was used as training data and 25% was used as testing data. Pearson's correlation analysis, performed using the ENMTools program, was used to assess the relationships among the 24 variables.^1^ To address multicollinearity, variables with correlation coefficients exceeding 0.9 and those contributing less according to pre-modeling were excluded. Additionally, factors contributing less than 1% were not included in the model. Model parameters were determined using the R program, specifically the kuenm package. This involved testing every possible combination of regularization multipliers and feature classes, including linear (L), quadratic (Q), product (P), threshold (T), and hinge (H). The model with the best fit was chosen by comparing the corrected Akaike Information Criterion (AICc) values and selecting the one with the lowest value. This criterion was used to avoid overfitting.^2,3^ The final ecological niche model was generated using Maxent software with cross-validation, using 25 replicates and a maximum of 10,000 iterations. The performance of the model was evaluated using the area under the Receiver Operating Characteristic (ROC) curve (AUC), which ranges from 0 to 1.^4^

**References**

1. Warren DL, Glor RE, Turelli M: ENMTools: a toolbox for comparative studies of environmental niche models. *Ecography* 2010, 33(3):607-611.

2. Cobos ME, Peterson AT, Barve N, Osorio-Olvera L: kuenm: an R package for detailed development of ecological niche models using Maxent. *PeerJ* 2019, 7:e6281.

3. Guevara L, Gerstner BE, Kass JM, Anderson RP: Toward ecologically realistic predictions of species distributions: A cross-time example from tropical montane cloud forests. *Glob Chang Biol* 2018, 24(4):1511-1522.

4. Phillips SJ, Anderson RP, Schapire RE: Maximum entropy modeling of species geographic distributions. *Ecological Modelling* 2006, 190(3):231-259.

# **Table S2. Ticks identified in the East African Community**

| Family name of ticks | Genus name of ticks | Species name of ticks |
| --- | --- | --- |
| [Argasidae](https://www.ncbi.nlm.nih.gov/Taxonomy/Browser/wwwtax.cgi?mode=Undef&id=6936&lvl=3&p=has_linkout&p=blast_url&p=genome_blast&lin=f&keep=1&srchmode=1&unlock) | *Argas* | *Argas vansomereni* |
|  |  | *Argas africolumbae* |
|  |  | *Argas persicus* |
|  |  | *Argas* sp. |
|  | *Ogadenus* | *Ogadenus brumpti* |
|  | *Carios* | *Carios vespertilionis* |
|  | *Chiropterargas* | *Chiropterargas confusus* |
|  | *Ornithodoros* | *Ornithodoros moubata* |
|  |  | *Ornithodoros porcinus* |
|  |  | *Ornithodoros savignyi* |
| [Ixodidae](https://www.ncbi.nlm.nih.gov/Taxonomy/Browser/wwwtax.cgi?mode=Undef&id=6939&lvl=3&p=has_linkout&p=blast_url&p=genome_blast&lin=f&keep=1&srchmode=1&unlock) | *Amblyomma* | *Amblyomma astrion* |
|  |  | *Amblyomma cohaerens* |
|  |  | *Amblyomma compressum* |
|  |  | *Amblyomma eburneum* |
|  |  | *Amblyomma exornatum* |
|  |  | *Amblyomma falsomarmoreum* |
|  |  | *Amblyomma flavomaculatum* |
|  |  | *Amblyomma gemma* |
|  |  | *Amblyomma hebraeum* |
|  |  | *Amblyomma latum* |
|  |  | *Amblyomma lepidum* |
|  |  | *Amblyomma marmoreum* |
|  |  | *Amblyomma nuttalli* |
|  |  | *Amblyomma paulopunctatum* |
|  |  | *Amblyomma personatum* |
|  |  | *Amblyomma pomposum* |
|  |  | *Amblyomma rhinocerotis* |
|  |  | *Amblyomma* sp. |
|  |  | *Amblyomma sparsum* |
|  |  | *Amblyomma splendidum* |
|  |  | *Amblyomma tholloni* |
|  |  | *Amblyomma transversale* |
|  |  | *Amblyomma variegatum* |
|  | *Haemaphysalis* | *Haemaphysalis aciculifer* |
|  |  | *Haemaphysalis bequaerti* |
|  |  | *Haemaphysalis calcarata* |
|  |  | *Haemaphysalis elliptica* |
|  |  | *Haemaphysalis hoodi* |
|  |  | *Haemaphysalis houyi* |
|  |  | *Haemaphysalis leachi* |
|  |  | *Haemaphysalis moreli* |
|  |  | *Haemaphysalis muhsamae* |
|  |  | *Haemaphysalis orientalis* |
|  |  | *Haemaphysalis paraleachi* |
|  |  | *Haemaphysalis parmata* |
|  |  | *Haemaphysalis punctaleachi* |
|  |  | *Haemaphysalis renschi* |
|  |  | *Haemaphysalis* sp. |
|  |  | *Haemaphysalis spinulosa* |
|  | *Hyalomma* | *Hyalomma albiparmatum* |
|  |  | *Hyalomma dromedarii* |
|  |  | *Hyalomma glabrum* |
|  |  | *Hyalomma impeltatum* |
|  |  | *Hyalomma marginatum* |
|  |  | *Hyalomma rufipes* |
|  |  | *Hyalomma* sp. |
|  |  | *Hyalomma truncatum* |
|  | *Ixodes* | *Ixodes alluaudi* |
|  |  | *Ixodes arabukiensis* |
|  |  | *Ixodes auriculaelongae* |
|  |  | *Ixodes brewsterae* |
|  |  | *Ixodes browningi* |
|  |  | *Ixodes cavipalpus* |
|  |  | *Ixodes cumulatimpunctatus* |
|  |  | *Ixodes daveyi* |
|  |  | *Ixodes elongatus* |
|  |  | *Ixodes festai* |
|  |  | *Ixodes latus* |
|  |  | *Ixodes lewisi* |
|  |  | *Ixodes moreli* |
|  |  | *Ixodes muniensis* |
|  |  | *Ixodes nairobiensis* |
|  |  | *Ixodes okapiae* |
|  |  | *Ixodes oldi* |
|  |  | *Ixodes pilosus* |
|  |  | *Ixodes procaviae* |
|  |  | *Ixodes rageaui* |
|  |  | *Ixodes rasus* |
|  |  | *Ixodes ricinus* |
|  |  | *Ixodes rotundatus* |
|  |  | *Ixodes schillingsi* |
|  |  | *Ixodes simplex* |
|  |  | *Ixodes* sp. |
|  |  | *Ixodes thomasae* |
|  |  | *Ixodes ugandanus* |
|  |  | *Ixodes vanidicus* |
|  |  | *Ixodes vespertilionis* |
|  |  | *Ixodes walkerae* |
|  | *Dermacentor* | *Dermacentor circumguttatus* |
|  |  | *Dermacentor rhinocerinus* |
|  | *Rhipicentor* | *Rhipicentor bicornis* |
|  | *Rhipicephalus* | *Rhipicephalus annulatus* |
|  |  | *Rhipicephalus appendiculatus* |
|  |  | *Rhipicephalus aquatilis* |
|  |  | *Rhipicephalus armatus* |
|  |  | *Rhipicephalus bequaerti* |
|  |  | *Rhipicephalus camicasi* |
|  |  | *Rhipicephalus capensis* |
|  |  | *Rhipicephalus carnivoralis* |
|  |  | *Rhipicephalus complanatus* |
|  |  | *Rhipicephalus compositus* |
|  |  | *Rhipicephalus decoloratus* |
|  |  | *Rhipicephalus deltoideus* |
|  |  | *Rhipicephalus distinctus* |
|  |  | *Rhipicephalus dux* |
|  |  | *Rhipicephalus humeralis* |
|  |  | *Rhipicephalus hurti* |
|  |  | *Rhipicephalus interventus* |
|  |  | *Rhipicephalus kochi* |
|  |  | *Rhipicephalus longicoxatus* |
|  |  | *Rhipicephalus longus* |
|  |  | *Rhipicephalus lunulatus* |
|  |  | *Rhipicephalus maculatus* |
|  |  | *Rhipicephalus masseyi* |
|  |  | *Rhipicephalus microplus* |
|  |  | *Rhipicephalus muehlensi* |
|  |  | *Rhipicephalus muhsamae* |
|  |  | *Rhipicephalus planus* |
|  |  | *Rhipicephalus praetextatus* |
|  |  | *Rhipicephalus punctatus* |
|  |  | *Rhipicephalus sanguineus* |
|  |  | *Rhipicephalus sculptus* |
|  |  | *Rhipicephalus senegalensis* |
|  |  | *Rhipicephalus simpsoni* |
|  |  | *Rhipicephalus simus* |
|  |  | *Rhipicephalus* sp. |
|  |  | *Rhipicephalus sulcatus* |
|  |  | *Rhipicephalus supertritus* |
|  |  | *Rhipicephalus tricuspis* |
|  |  | *Rhipicephalus turanicus* |
|  |  | *Rhipicephalus zambeziensis* |
|  |  | *Rhipicephalus ziemanni* |
|  |  | *Rhipicephalus congolensis* |
|  |  | *Rhipicephalus evertsi* |
|  |  | *Rhipicephalus pravus* |
|  |  | *Rhipicephalus pulchellus* |
|  | *Cosmiomma* | *Cosmiomma hippopotamensis* |
| [Nuttalliellidae](https://www.ncbi.nlm.nih.gov/Taxonomy/Browser/wwwtax.cgi?mode=Undef&id=297309&lvl=3&p=has_linkout&p=blast_url&p=genome_blast&lin=f&keep=1&srchmode=1&unlock) | *Nuttalliella* | *Nuttalliella namaqua* |
| Unkown tick | Unkown tick | Unkown tick |

# **Fig. S2. Geographic distribution maps of remaining ticks in the East African Community**

The base map is landcover. Recorded locations of tick species in the genera *Rhipicentor, Ornithodoros, Ogadenus, Nuttalliella, Dermacentor, Cosmiomma, Chiropterargas, Argas* and Unkown tick*.*


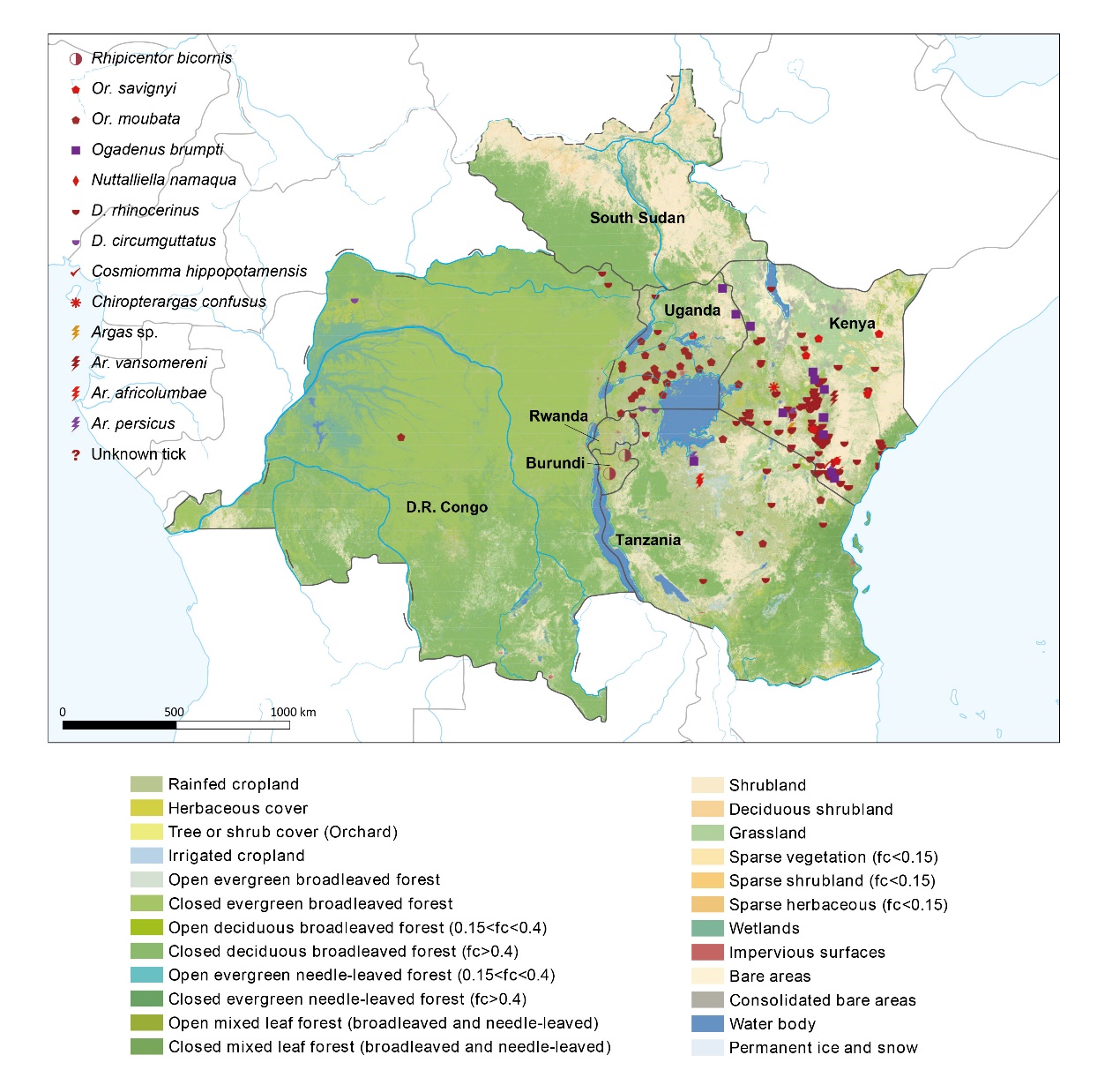


# **Fig. S3. Geographic distribution maps of ticks in the East African Community based on altitude**

The base map is altitude (unit: meters). Recorded locations of each tick genus.


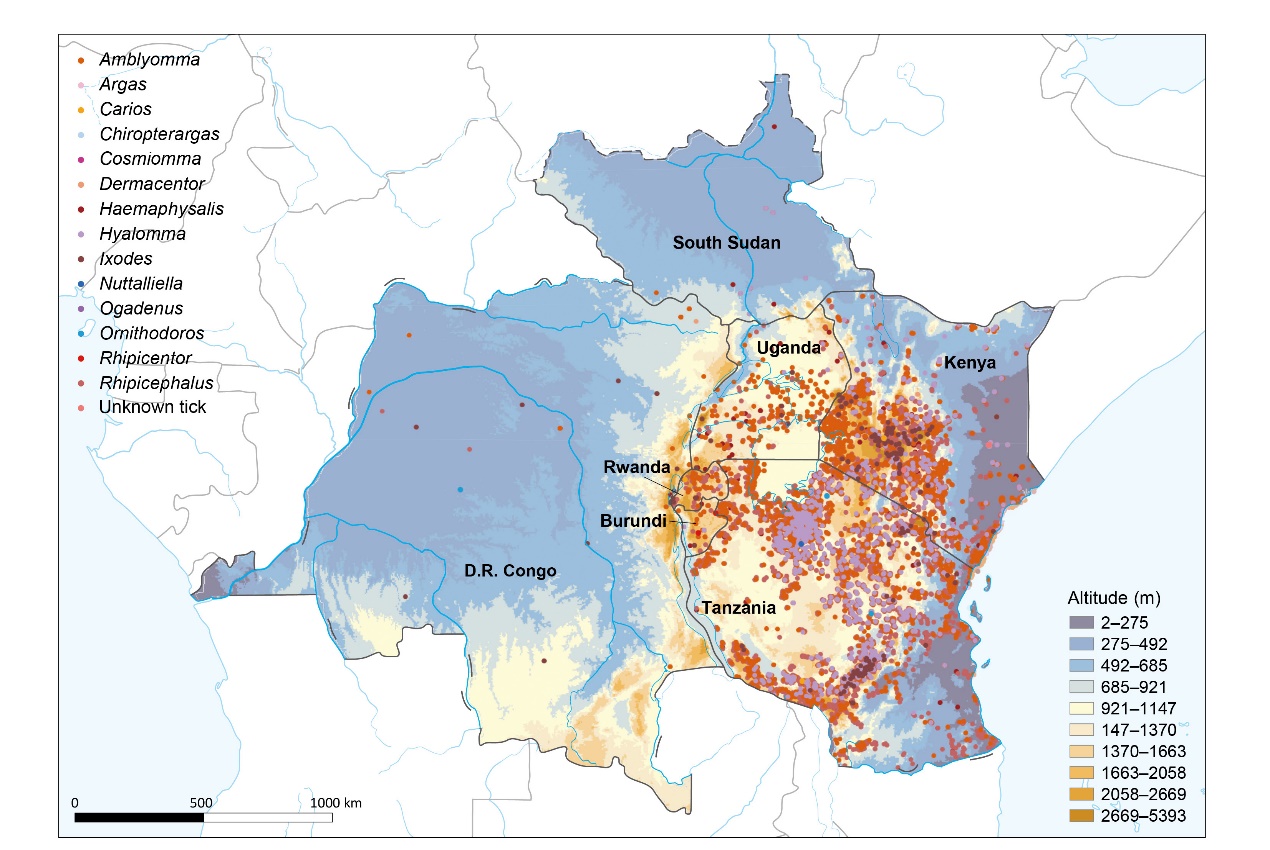


# **Fig. S4. Geographic distribution maps of ticks in the East African Community based on population count**

The base map is population count recorded locations of each tick genus.


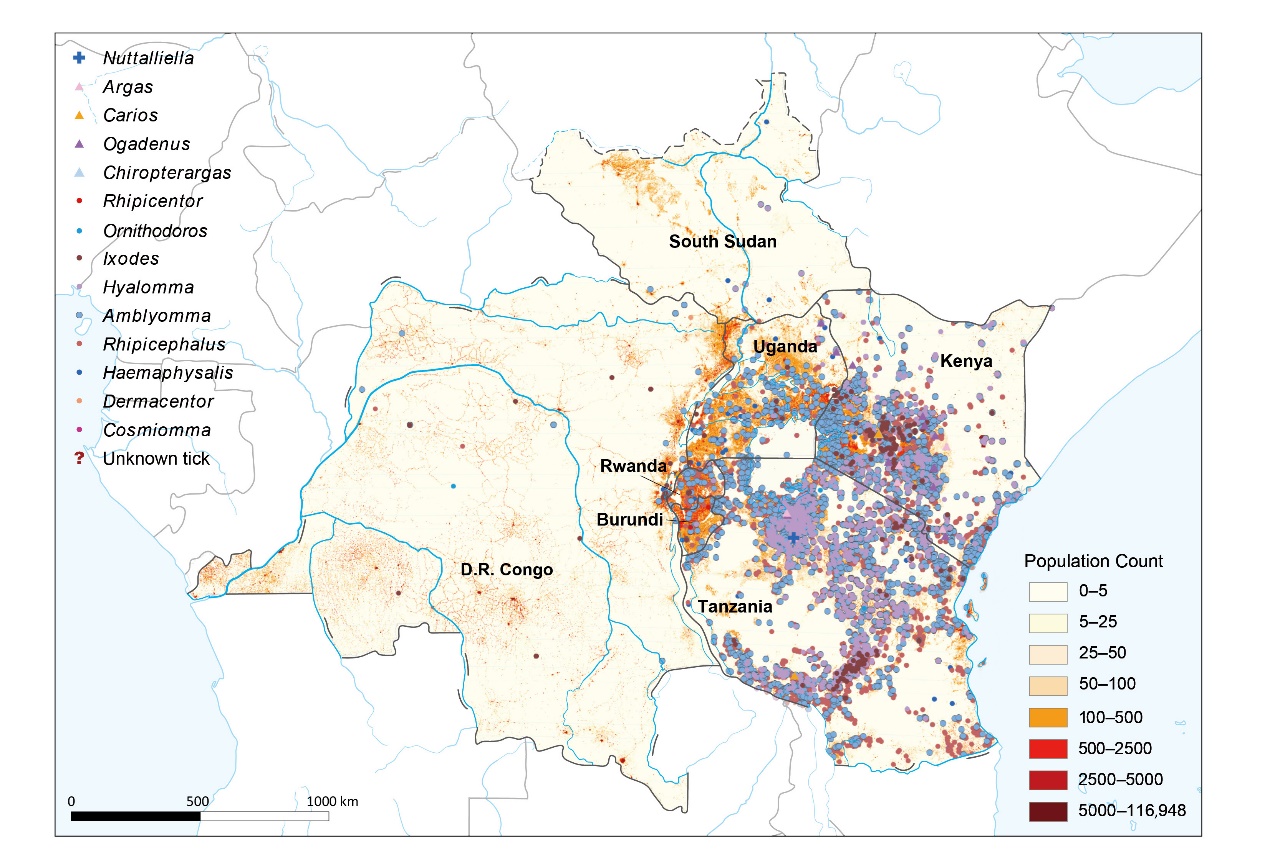


# **Fig. S5. Sankey diagram of hosts, tick species and microbes in East African Community**


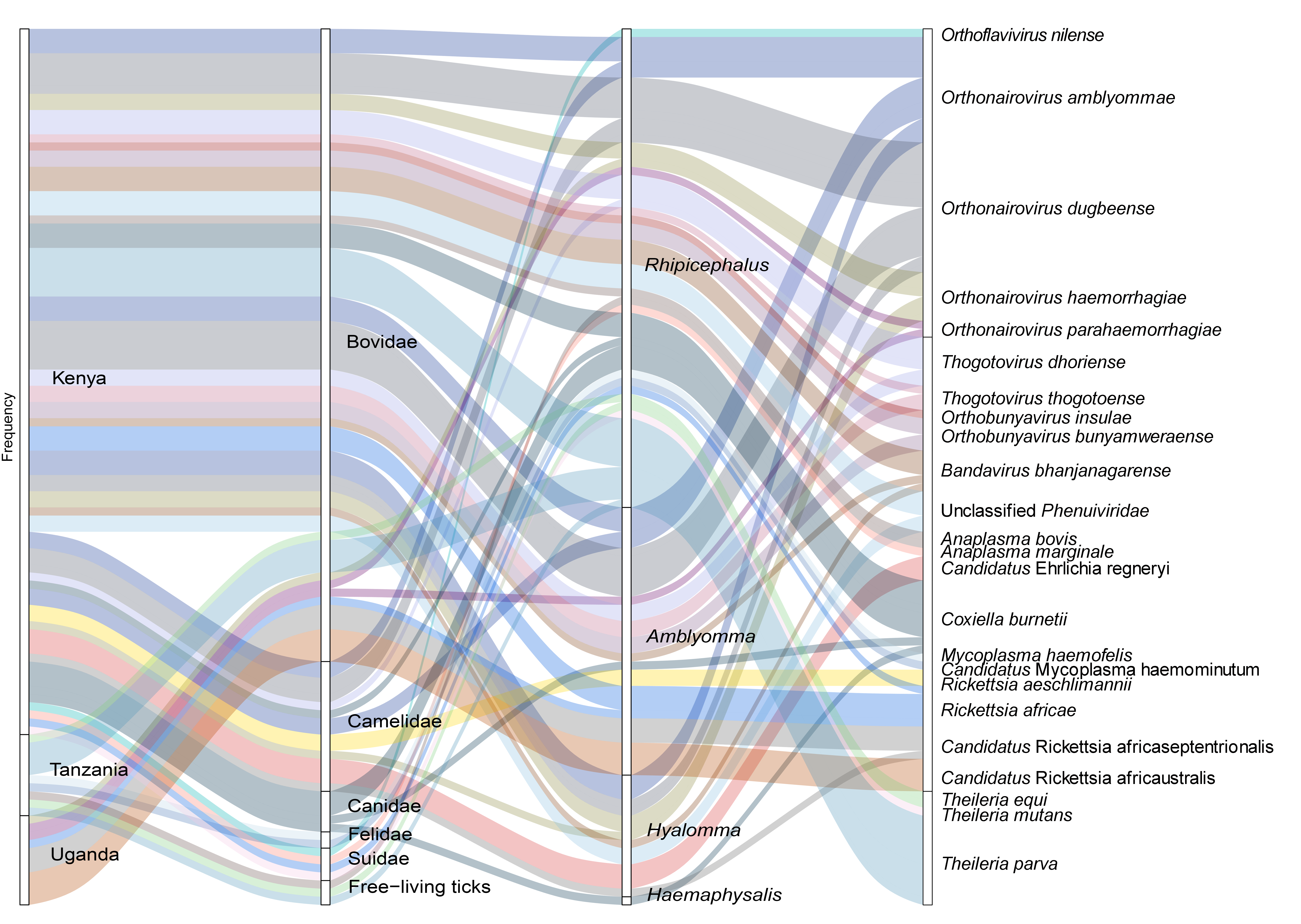


# **Fig. S6. Matrix of tick species and hosts in the East African Community**


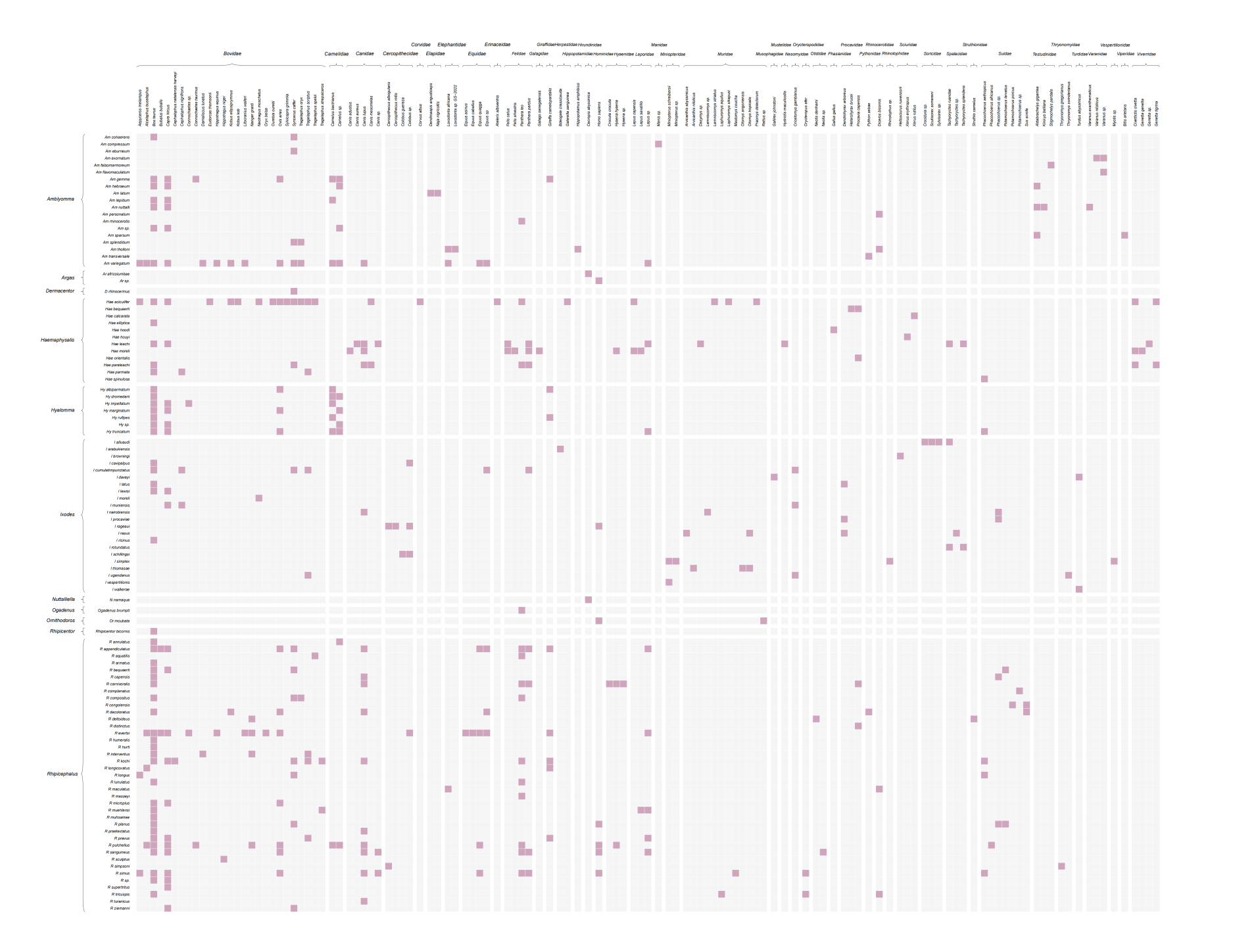


# **Fig. S7. Correlation analysis of tick species and tick-associated microbes**


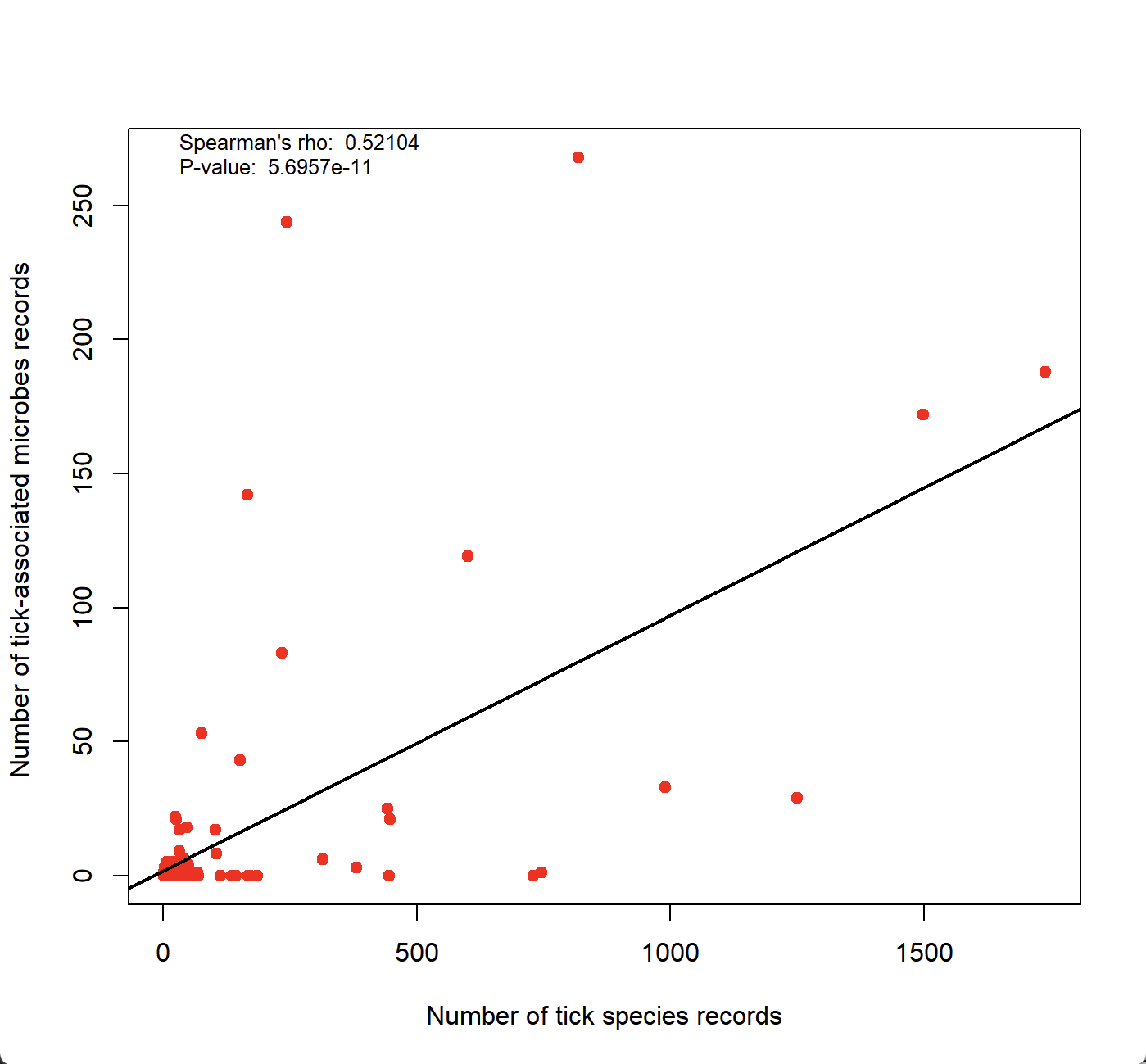


Sperman r = 0.5210352, *P* < 0.0001

# **Table S3. The estimated positive rate and 95% confidence interval (CI) for each microbe in a tick species**

| Tick-related microbes | Positive rate | 95% CI | Combined studies |
| --- | --- | --- | --- |
| Virus |  |  |  |
| Asfarviridae |  |  |  |
| *African swine fever virus* |  |  |  |
| *Ornithodoros porcinus* | NA | NA | NA |
| Flaviviridae |  |  |  |
| *Mogiana tick virus* |  |  |  |
| *Amblyomma nuttalli* | NA | NA | NA |
| *Amblyomma sparsum* | NA | NA | NA |
| *Amblyomma* sp. | NA | NA | NA |
| *Rhipicephalus appendiculatus* | NA | NA | NA |
| *Orthoflavivirus nilense* |  |  |  |
| *Rhipicephalus pulchellus* | 0.1449 | NA | 1 |
| Nairoviridae |  |  |  |
| *Orthonairovirus amblyommae* |  |  |  |
| *Amblyomma gemma* | 0.0986 | NA | 1 |
| *Amblyomma variegatum* | 0.0513 | NA | 1 |
| *Hyalomma truncatum* | 0.0678 | NA | 1 |
| *Rhipicephalus pulchellus* | 0.1218 | NA | 1 |
| *Orthonairovirus dugbeense* |  |  |  |
| *Amblyomma cohaerens* | 0.0385 | NA | 1 |
| *Amblyomma gemma* | NA | NA | NA |
| *Amblyomma hebraeum* | 0.0065 | NA | 1 |
| *Amblyomma lepidum* | 0.0769 | NA | 1 |
| *Amblyomma variegatum* | 0.1024 | 0.0000~0.3225 | 2 |
| *Hyalomma truncatum* | 0.0100 | NA | 1 |
| *Rhipicephalus annulatus* | NA | NA | NA |
| *Rhipicephalus appendiculatus* | 0.0005 | NA | 1 |
| *Rhipicephalus pulchellus* | 0.0022 | NA | 1 |
| *Orthonairovirus haemorrhagiae* |  |  |  |
| *Hyalomma marginatum* | 0.0733 | NA | 1 |
| *Hyalomma rufipes* | 0.1500 | NA | 1 |
| *Hyalomma truncatum* | 0.0303 | NA | 1 |
| *Rhipicephalus appendiculatus* | 0.1000 | NA | 1 |
| *Rhipicephalus decoloratus* | 0.0553 | 0.0000~0.1574 | 2 |
| *Rhipicephalus* sp. | NA | NA | NA |
| *Orthonairovirus parahaemorrhagiae* |  |  |  |
| *Amblyomma variegatum* | 0.0757 | NA | 1 |
| *Rhipicephalus appendiculatus* | 0.0441 | NA | 1 |
| Orthomyxoviridae |  |  |  |
| *Thogotovirus dhoriense* |  |  |  |
| *Amblyomma gemma* | 0.0036 | NA | 1 |
| *Rhipicephalus pulchellus* | NA | NA | NA |
| *Thogotovirus thogotoense* |  |  |  |
| *Amblyomma gemma* | 0.6667 | NA | 1 |
| *Amblyomma variegatum* | 0.0001 | NA | 1 |
| *Rhipicephalus appendiculatus* | 0.0005 | NA | 1 |
| Peribunyaviridae |  |  |  |
| *Orthobunyavirus bunyamweraense* |  |  |  |
| *Amblyomma gemma* | 0.0075 | NA | 1 |
| *Rhipicephalus pulchellus* | 0.1250 | NA | 1 |
| *Orthobunyavirus insulae* |  |  |  |
| *Rhipicephalus evertsi* | NA | NA | NA |
| Phenuiviridae |  |  |  |
| *Balambala tick virus* |  |  |  |
| *Hyalomma dromedarii* | NA | NA | NA |
| *Hyalomma rufipes* | NA | NA | NA |
| *Hyalomma truncatum* | NA | NA | NA |
| *Bandavirus bhanjanagarense* |  |  |  |
| *Amblyomma variegatum* | 0.0003 | NA | 1 |
| *Hyalomma truncatum* | 0.5000 | NA | 1 |
| *Rhipicephalus appendiculatus* | 0.1251 | 0.0000~0.5488 | 2 |
| *Rhipicephalus decoloratus* | 0.5000 | NA | 1 |
| *Iftin tick virus* |  |  |  |
| *Hyalomma dromedarii* | NA | NA | NA |
| *Phlebovirus ntepesense* |  |  |  |
| *Hyalomma truncatum* | NA | NA | NA |
| *Phlebovirus bogoriaense* |  |  |  |
| *Hyalomma truncatum* | NA | NA | NA |
| *Rhipicephalus appendiculatus* | 0.5556 | NA | 1 |
| *Tick phlebovirus* |  |  |  |
| *Hyalomma marginatum* | NA | NA | NA |
| *Hyalomma truncatum* | NA | NA | NA |
| *Rhipicephalus appendiculatus* | NA | NA | NA |
| *Rhipicephalus evertsi* | NA | NA | NA |
| *Rhipicephalus pulchellus* | NA | NA | NA |
| Unclassified *Phenuiviridae* |  |  |  |
| *Hyalomma marginatum* | 0.0303 | NA | 1 |
| *Hyalomma truncatum* | 0.3913 | NA | 1 |
| *Rhipicephalus appendiculatus* | 0.0547 | NA | 1 |
| *Rhipicephalus evertsi* | 0.0571 | NA | 1 |
| *Rhipicephalus pulchellus* | 0.5000 | NA | 1 |
| Sedoreoviridae |  |  |  |
| *St Croix River virus* |  |  |  |
| *Rhipicephalus appendiculatus* | NA | NA | NA |
| Tymoviridae |  |  |  |
| *Guarapuava tymovirus-like 1 virus* |  |  |  |
| unknown *tick* | NA | NA | NA |
| unclassified *Riboviria* |  |  |  |
| *Bole tick virus 4* |  |  |  |
| *Hyalomma dromedarii* | NA | NA | NA |
| *Hyalomma rufipes* | NA | NA | NA |
| *Hyalomma truncatum* | NA | NA | NA |
| *Liman tick virus* |  |  |  |
| *Hyalomma rufipes* | NA | NA | NA |
| Bacterium |  |  |  |
| Anaplasmataceae |  |  |  |
| *Anaplasma bovis* |  |  |  |
| *Rhipicephalus evertsi* | NA | NA | NA |
| *Rhipicephalus praetextatus* | NA | NA | NA |
| *Anaplasma marginale* |  |  |  |
| *Rhipicephalus decoloratus* | 0.0370 | NA | 1 |
| *Anaplasma ovis* |  |  |  |
| *Amblyomma lepidum* | NA | NA | NA |
| *Rhipicephalus camicasi* | NA | NA | NA |
| *Anaplasma platys* |  |  |  |
| *Rhipicephalus evertsi* | NA | NA | NA |
| *Candidatus* Anaplasma camelii |  |  |  |
| *Amblyomma* sp. | NA | NA | NA |
| *Hyalomma dromedarii* | NA | NA | NA |
| *Hyalomma impeltatum* | NA | NA | NA |
| *Hyalomma rufipes* | NA | NA | NA |
| *Rhipicephalus camicasi* | NA | NA | NA |
| *Anaplasma* sp. |  |  |  |
| *Amblyomma gemma* | NA | NA | NA |
| *Amblyomma tholloni* | NA | NA | NA |
| *Amblyomma variegatum* | NA | NA | NA |
| *Haemaphysalis parmata* | NA | NA | NA |
| *Haemaphysalis punctaleachi* | NA | NA | NA |
| *Hyalomma rufipes* | NA | NA | NA |
| *Rhipicephalus appendiculatus* | NA | NA | NA |
| *Rhipicephalus camicasi* | NA | NA | NA |
| *Rhipicephalus evertsi* | NA | NA | NA |
| *Rhipicephalus maculatus* | NA | NA | NA |
| *Rhipicephalus* sp. | NA | NA | NA |
| *Ehrlichia chaffeensis* |  |  |  |
| *Amblyomma lepidum* | NA | NA | NA |
| *Ehrlichia minasensis* |  |  |  |
| *Rhipicephalus* sp. | NA | NA | NA |
| *Ehrlichia ruminantium* |  |  |  |
| *Amblyomma gemma* | NA | NA | NA |
| *Amblyomma variegatum* | NA | NA | NA |
| *Candidatus* Ehrlichia regneryi |  |  |  |
| *Hyalomma dromedarii* | 0.0866 | NA | 1 |
| *Hyalomma impeltatum* | 0.1364 | NA | 1 |
| *Hyalomma rufipes* | 0.1833 | NA | 1 |
| *Ehrlichia* sp. |  |  |  |
| *Amblyomma eburneum* | NA | NA | NA |
| *Amblyomma gemma* | NA | NA | NA |
| *Amblyomma tholloni* | NA | NA | NA |
| *Amblyomma variegatum* | NA | NA | NA |
| *Haemaphysalis muhsamae* | NA | NA | NA |
| *Hyalomma rufipes* | NA | NA | NA |
| *Ixodes* sp. | NA | NA | NA |
| *Rhipicephalus congolensis* | NA | NA | NA |
| *Rhipicephalus humeralis* | NA | NA | NA |
| *Rhipicephalus pravus* | NA | NA | NA |
| *Rhipicephalus pulchellus* | NA | NA | NA |
| *Rhipicephalus* sp. | NA | NA | NA |
| Borreliaceae |  |  |  |
| *Borrelia* sp. |  |  |  |
| *Haemaphysalis parmata* | NA | NA | NA |
| Coxiellaceae |  |  |  |
| *Coxiella burnetii* |  |  |  |
| *Amblyomma variegatum* | 0.2250 | NA | 1 |
| *Haemaphysalis leachi* | 0.2167 | NA | 1 |
| *Rhipicephalus appendiculatus* | 0.2257 | 0.0000~0.5497 | 2 |
| *Rhipicephalus decoloratus* | 0.5200 | NA | 1 |
| *Rhipicephalus evertsi* | 0.1471 | NA | 1 |
| *Rhipicephalus pulchellus* | 0.0719 | 0.0000~0.1602 | 2 |
| *Rhipicephalus sanguineus* | 0.0800 | NA | 1 |
| *Rhipicephalus* sp. | NA | NA | NA |
| *Coxiella* sp. |  |  |  |
| *Amblyomma eburneum* | NA | NA | NA |
| *Amblyomma gemma* | NA | NA | NA |
| *Amblyomma variegatum* | NA | NA | NA |
| *Amblyomma* sp. | NA | NA | NA |
| *Haemaphysalis leachi* | NA | NA | NA |
| *Rhipicephalus appendiculatus* | NA | NA | NA |
| *Rhipicephalus evertsi* | NA | NA | NA |
| *Rhipicephalus pulchellus* | NA | NA | NA |
| *Rhipicephalus* sp. | NA | NA | NA |
| Mycoplasmataceae |  |  |  |
| *Mycoplasma haemofelis* |  |  |  |
| *Rhipicephalus sanguineus* | 0.6667 | NA | 1 |
| *Candidatus*  Mycoplasma haemominutum |  |  |  |
| *Rhipicephalus sanguineus* | 0.5000 | NA | 1 |
| Rickettsiaceae |  |  |  |
| *Rickettsia aeschlimannii* |  |  |  |
| *Amblyomma gemma* | 0.1085 | NA | 1 |
| *Amblyomma lepidum* | 0.3286 | NA | 1 |
| *Hyalomma impeltatum* | NA | NA | NA |
| *Hyalomma rufipes* | NA | NA | NA |
| *Rickettsia africae* |  |  |  |
| *Amblyomma gemma* | 0.3333 | NA | 1 |
| *Amblyomma lepidum* | NA | NA | NA |
| *Amblyomma variegatum* | 0.9409 | 0.8434~1.0000 | 3 |
| *Rhipicephalus decoloratus* | 0.0278 | NA | 1 |
| *Rickettsia conorii* |  |  |  |
| *Rickettsia hoogstraalii* |  |  |  |
| *Ornithodoros moubata* | NA | NA | NA |
| *Rickettsia massiliae* |  |  |  |
| *Rickettsia sibirica* |  |  |  |
| *Amblyomma variegatum* | NA | NA | NA |
| *Hyalomma truncatum* | NA | NA | NA |
| *Rhipicephalus appendiculatus* | NA | NA | NA |
| *Rhipicephalus evertsi* | NA | NA | NA |
| *Rhipicephalus pulchellus* | NA | NA | NA |
| *Rickettsia slovaca* |  |  |  |
| *Candidatus*  Rickettsia africaseptentrionalis |  |  |  |
| *Amblyomma variegatum* | 0.9833 | NA | 1 |
| *Hyalomma rufipes* | 0.3466 | NA | 1 |
| *Candidatus* Rickettsia africaustralis |  |  |  |
| *Amblyomma variegatum* | 0.9625 | NA | 1 |
| *Rickettsia* sp. |  |  |  |
| *Amblyomma eburneum* | NA | NA | NA |
| *Amblyomma gemma* | NA | NA | NA |
| *Amblyomma hebraeum* | NA | NA | NA |
| *Amblyomma paulopunctatum* | NA | NA | NA |
| *Amblyomma tholloni* | NA | NA | NA |
| *Amblyomma variegatum* | NA | NA | NA |
| *Amblyomma* sp. | NA | NA | NA |
| *Hyalomma truncatum* | NA | NA | NA |
| *Hyalomma* sp. | NA | NA | NA |
| *Ixodes muniensis* | NA | NA | NA |
| *Ixodes rasus* | NA | NA | NA |
| *Rhipicephalus annulatus* | NA | NA | NA |
| *Rhipicephalus appendiculatus* | 0.1250 | NA | 1 |
| *Rhipicephalus dux* | NA | NA | NA |
| *Rhipicephalus evertsi* | NA | NA | NA |
| *Rhipicephalus maculatus* | NA | NA | NA |
| *Rhipicephalus pulchellus* | NA | NA | NA |
| *Rhipicephalus* sp. | NA | NA | NA |
| Protist |  |  |  |
| Babesiidae |  |  |  |
| *Babesia bigemina* |  |  |  |
| *Rhipicephalus decoloratus* | NA | NA | NA |
| *Babesia caballi* |  |  |  |
| *Amblyomma variegatum* | NA | NA | NA |
| *Rhipicephalus pulchellus* | NA | NA | NA |
| *Babesia* sp. |  |  |  |
| *Haemaphysalis parmata* | NA | NA | NA |
| *Ixodes muniensis* | NA | NA | NA |
| Hepatozoidae |  |  |  |
| *Hepatozoon canis* |  |  |  |
| *Rhipicephalus decoloratus* | NA | NA | NA |
| *Hepatozoon fitzsimonsi* |  |  |  |
| *Amblyomma sparsum* | NA | NA | NA |
| Theileridae |  |  |  |
| *Theileria bicornis* |  |  |  |
| *Amblyomma tholloni* | NA | NA | NA |
| *Theileria buffeli* |  |  |  |
| *Rhipicephalus appendiculatus* | NA | NA | NA |
| *Theileria* cf. *velifera A* |  |  |  |
| *Amblyomma eburneum* | NA | NA | NA |
| *Theileria* cf. *velifera* JV-2016 |  |  |  |
| *Amblyomma eburneum* | NA | NA | NA |
| *Theileria equi* |  |  |  |
| *Rhipicephalus appendiculatus* | 0.1004 | NA | 1 |
| *Rhipicephalus evertsi* | 0.4375 | NA | 1 |
| *Theileria mutans* |  |  |  |
| *Rhipicephalus decoloratus* | 0.1111 | NA | 1 |
| *Theileria parva* |  |  |  |
| *Rhipicephalus appendiculatus* | 0.0990 | 0.0601~0.1378 | 9 |
| *Rhipicephalus* sp. | NA | NA | NA |
| *Theileria taurotragi* |  |  |  |
| *Rhipicephalus appendiculatus* | NA | NA | NA |
| *Rhipicephalus* sp. | NA | NA | NA |
| *Theileria velifera* |  |  |  |
| *Amblyomma gemma* | NA | NA | NA |
| *Amblyomma tholloni* | NA | NA | NA |
| *Amblyomma variegatum* | NA | NA | NA |
| *Theileria* sp. JV-2016 |  |  |  |
| *Rhipicephalus evertsi* | NA | NA | NA |
| *Theileria* sp. |  |  |  |
| *Amblyomma tholloni* | NA | NA | NA |

# **Fig. S8. Meta-analysis of positive rate of each tick species-associated microbe in a tick species**

**8-1 Positive rate of *Orthonairovirus dugbeense* in *Amblyomma variegatum***

***
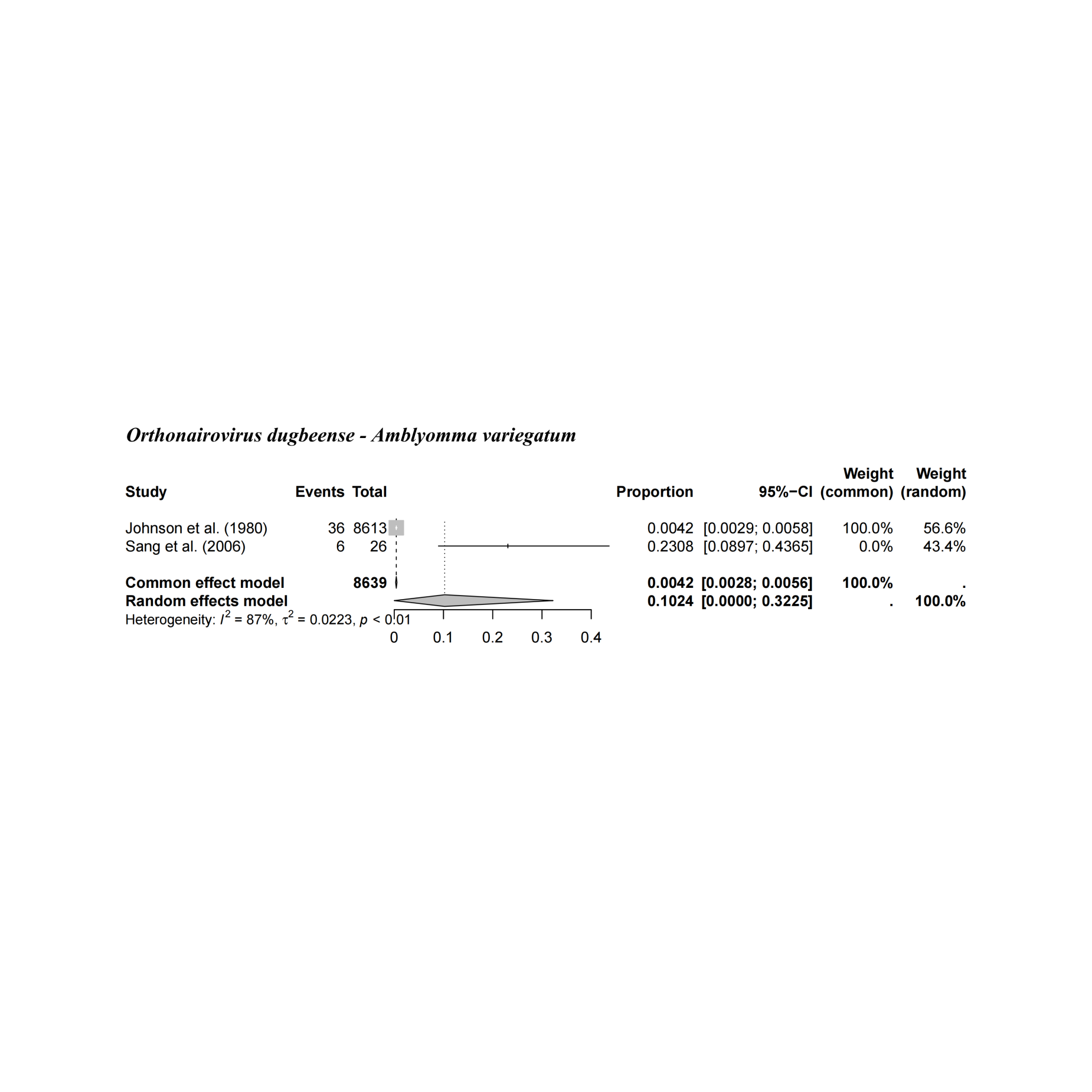
***

**8-2 Positive rate of *Orthonairovirus haemorrhagiae* in *Rhipicephalus decoloratus***


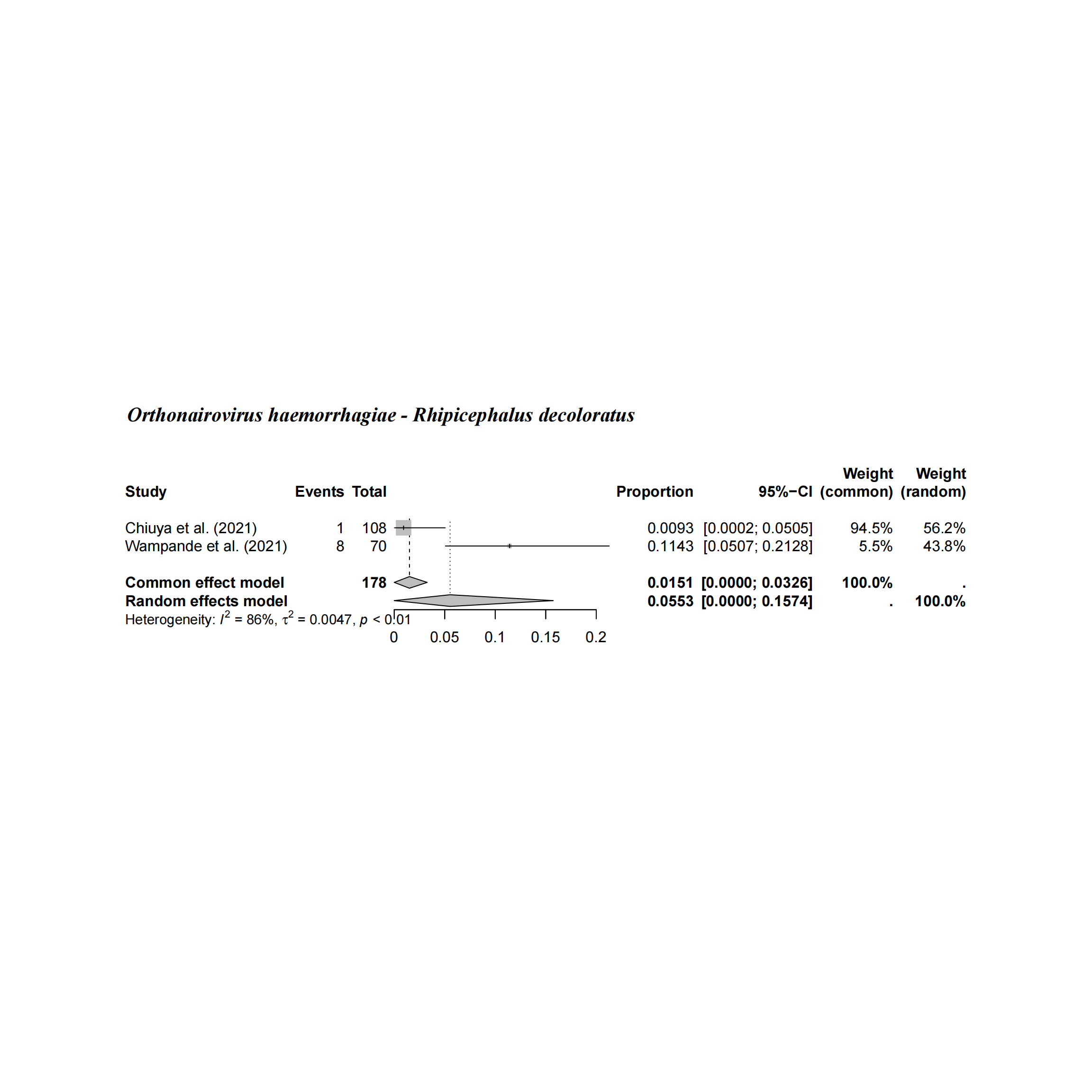


**8-3 Positive rate of *Bandavirus bhanjanagarense* in *Rhipicephalus appendiculatus***

*
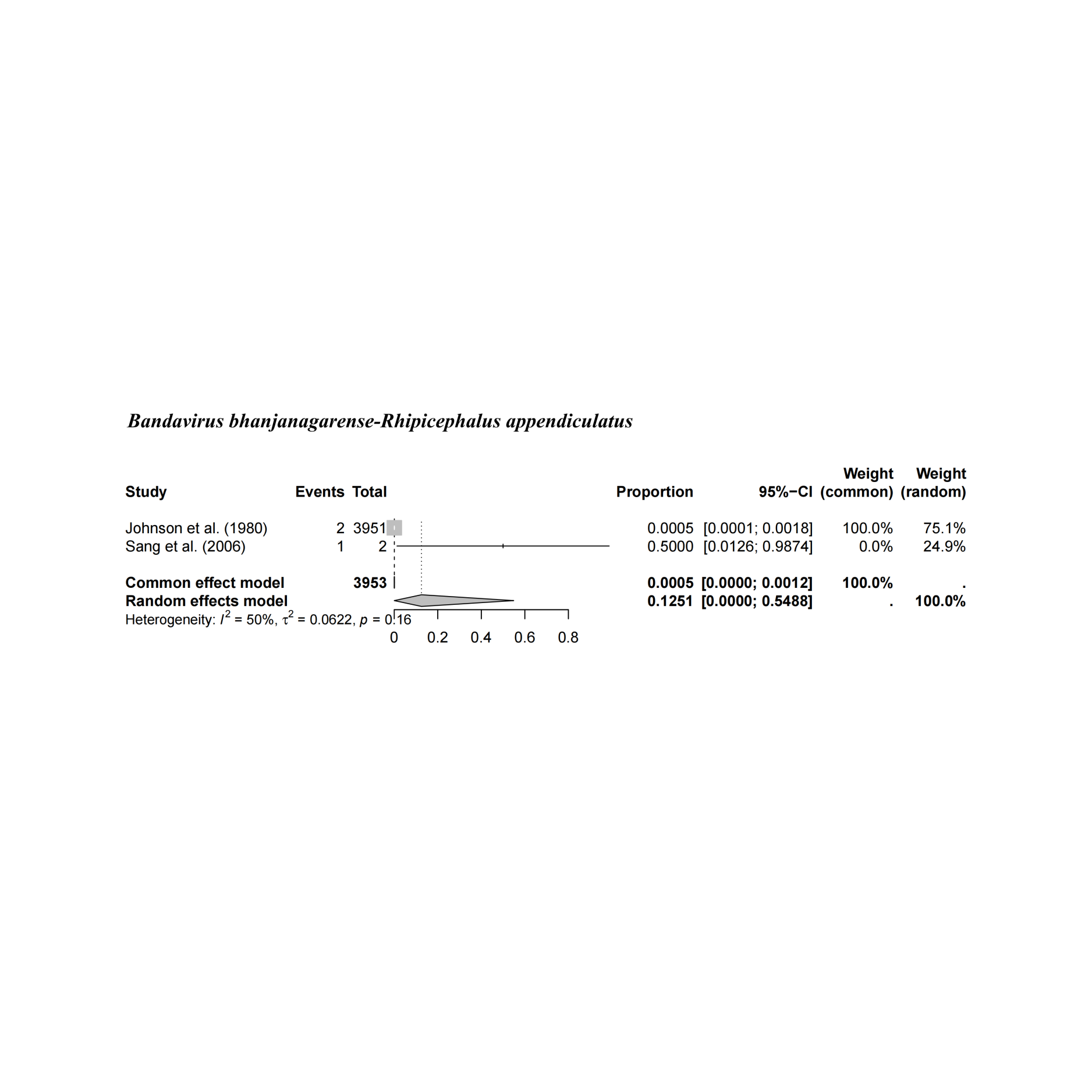
*

**8-4 Positive rate of *Coxiella burnetii* in *Rhipicephalus appendiculatus***


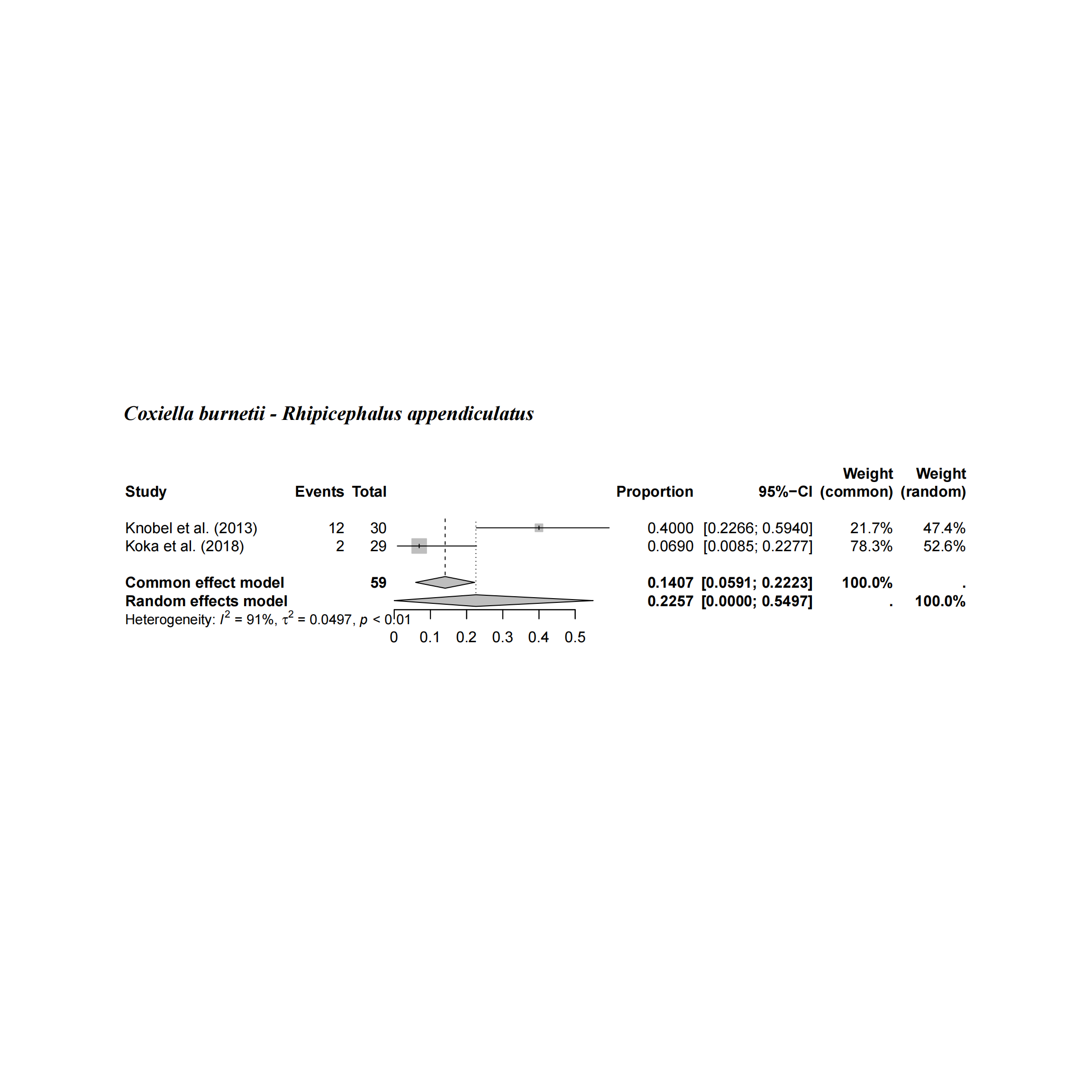


**8-5 Positive rate of *Coxiella burnetii* in *Rhipicephalus pulchellus***


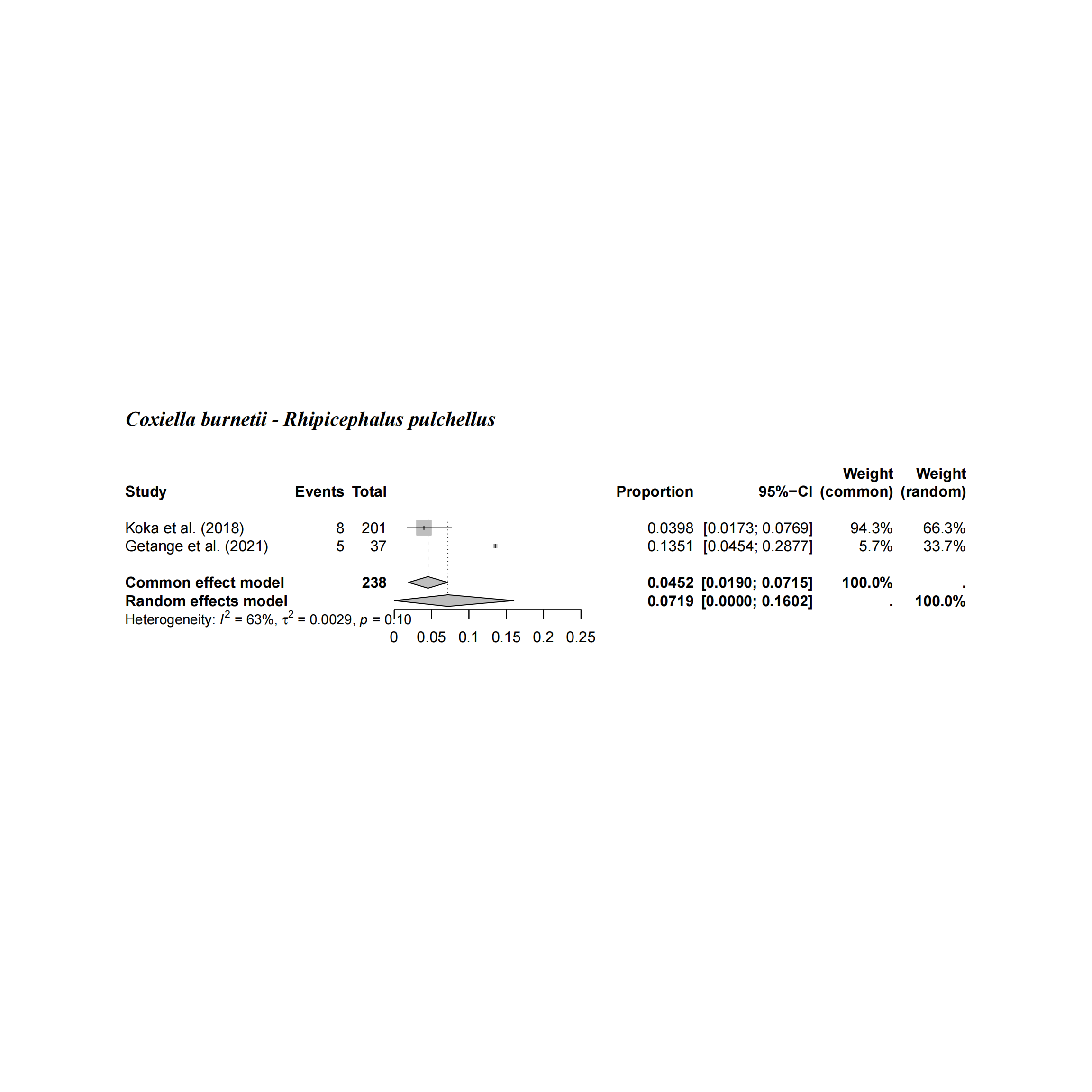


**8-6 Positive rate of *Rickettsia africae* in *Amblyomma variegatum***


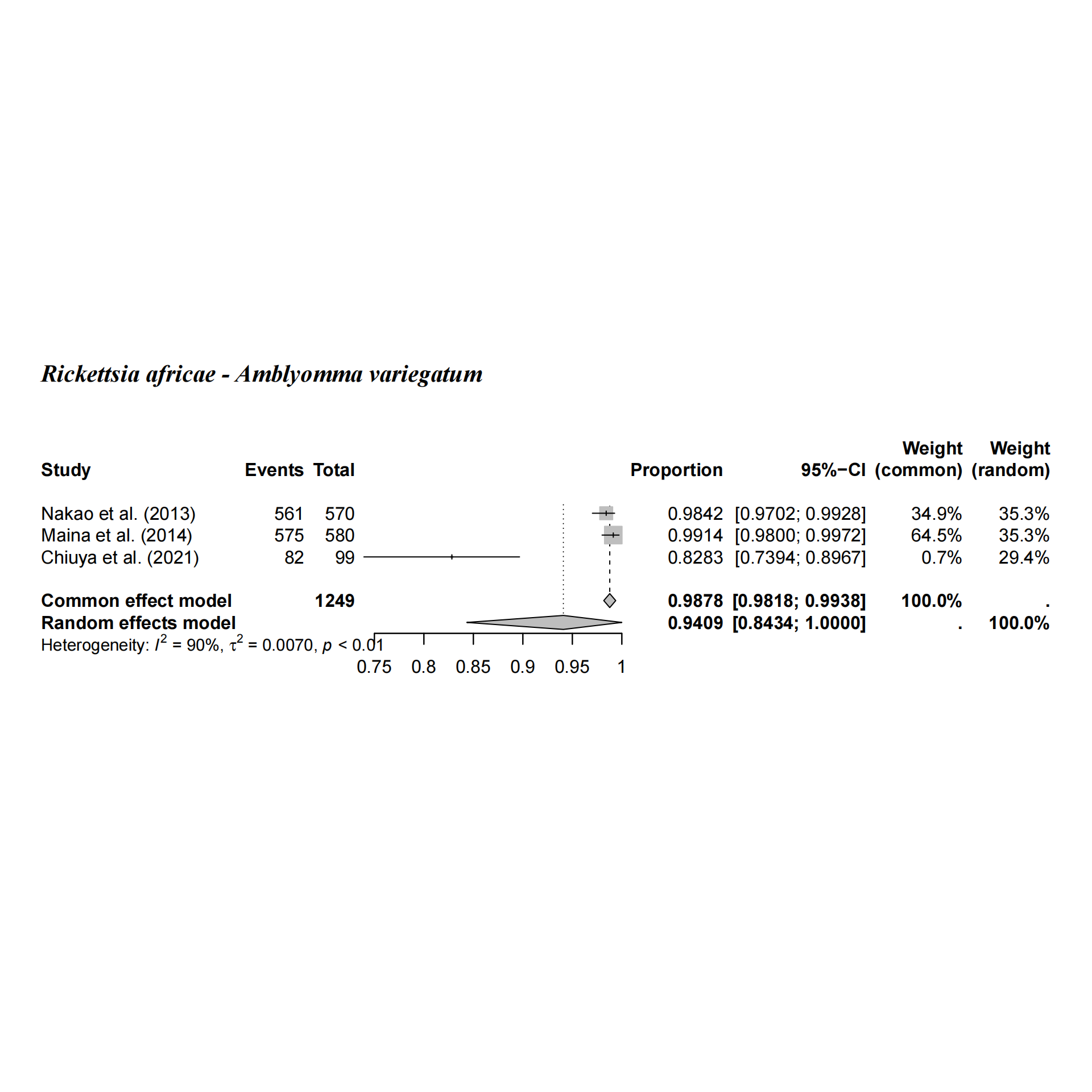


**8-7 Positive rate of *Theileria parva* in *Rhipicephalus appendiculatus***

***
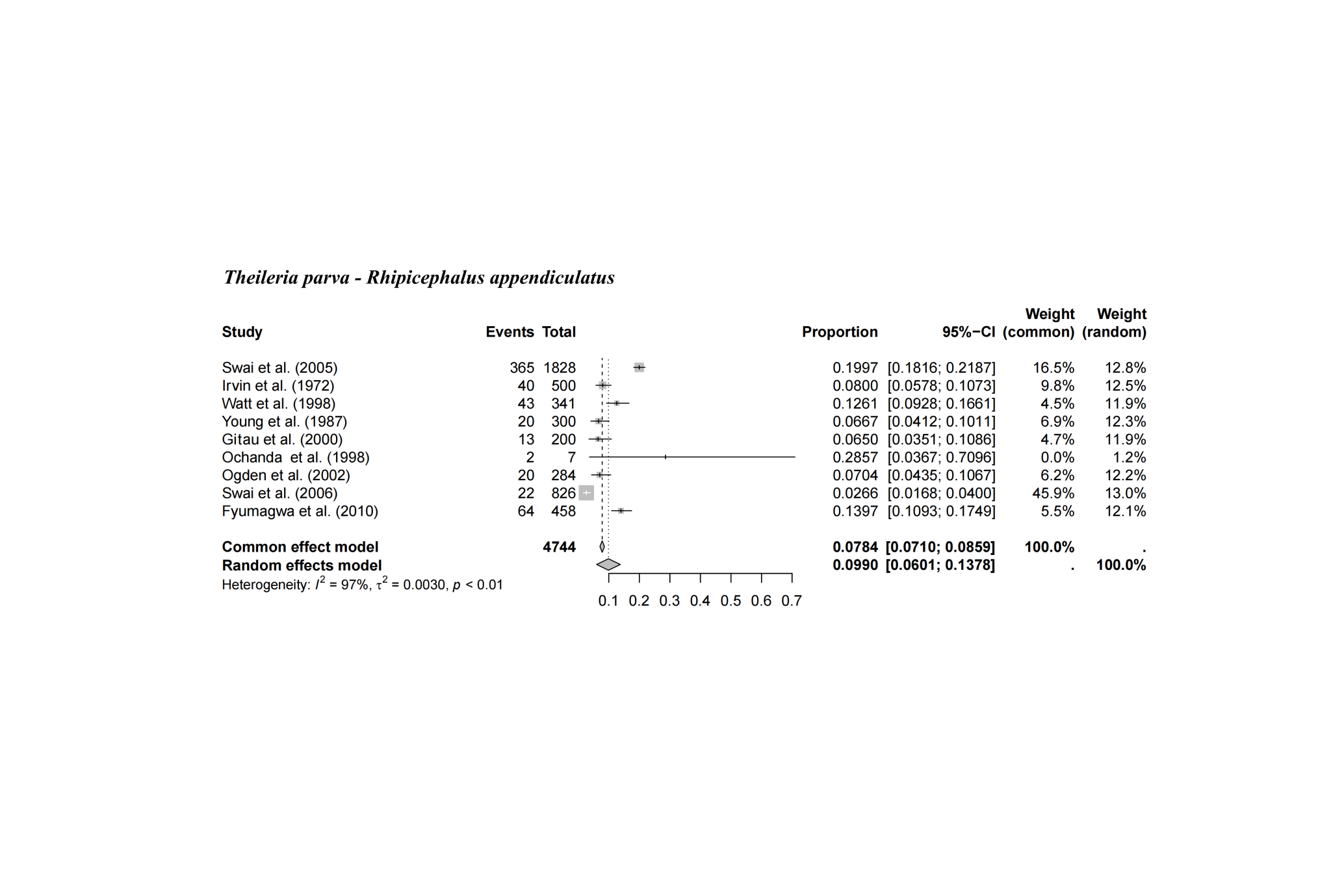
***

# **Fig. S9. The forest Fig. of combined positive rates for each microbe in all positive tick species**

**9-1 Positive rate of *Bandavirus bhanjanagarense***


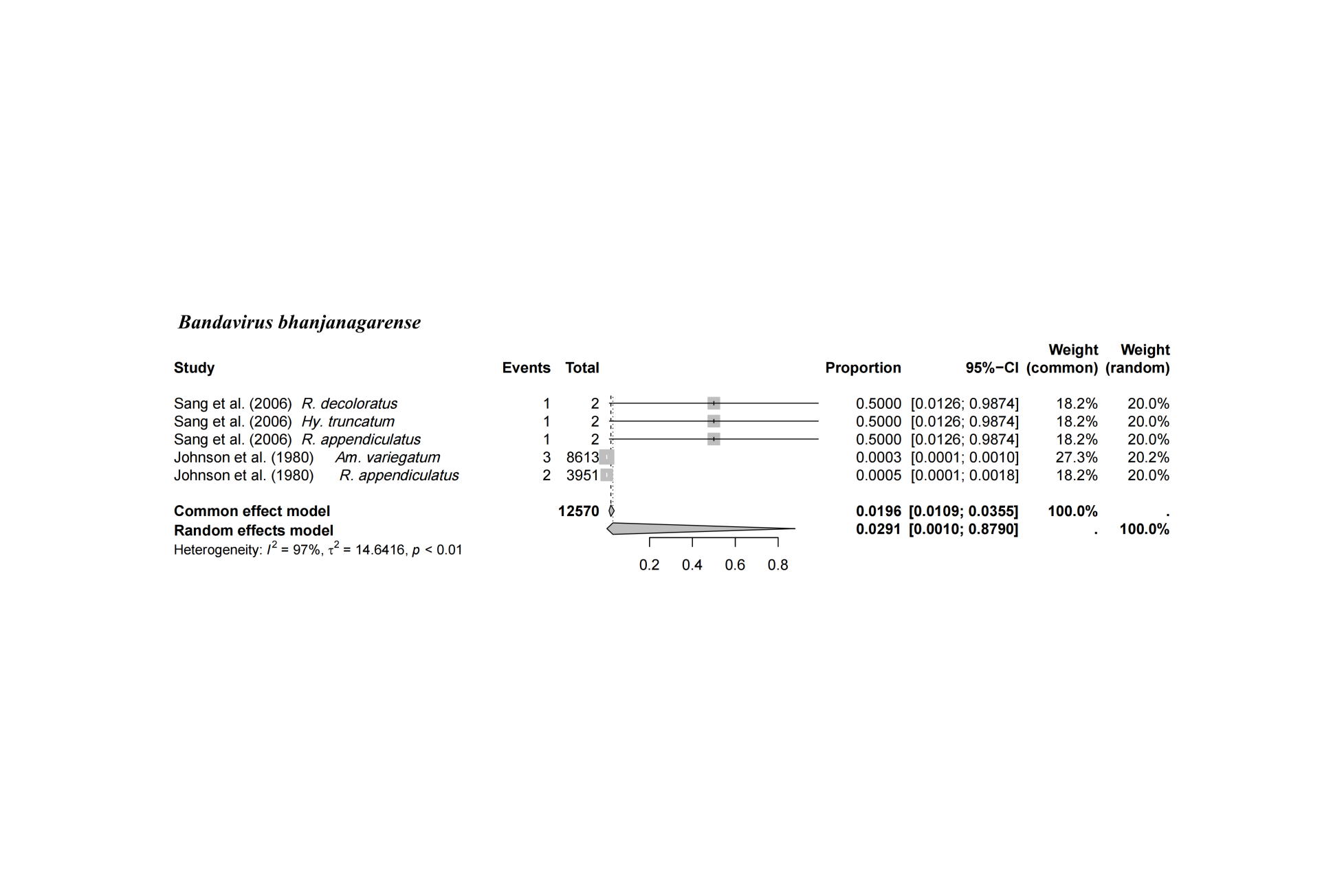


**9-2 Positive rate of *Orthonairovirus amblyommae***


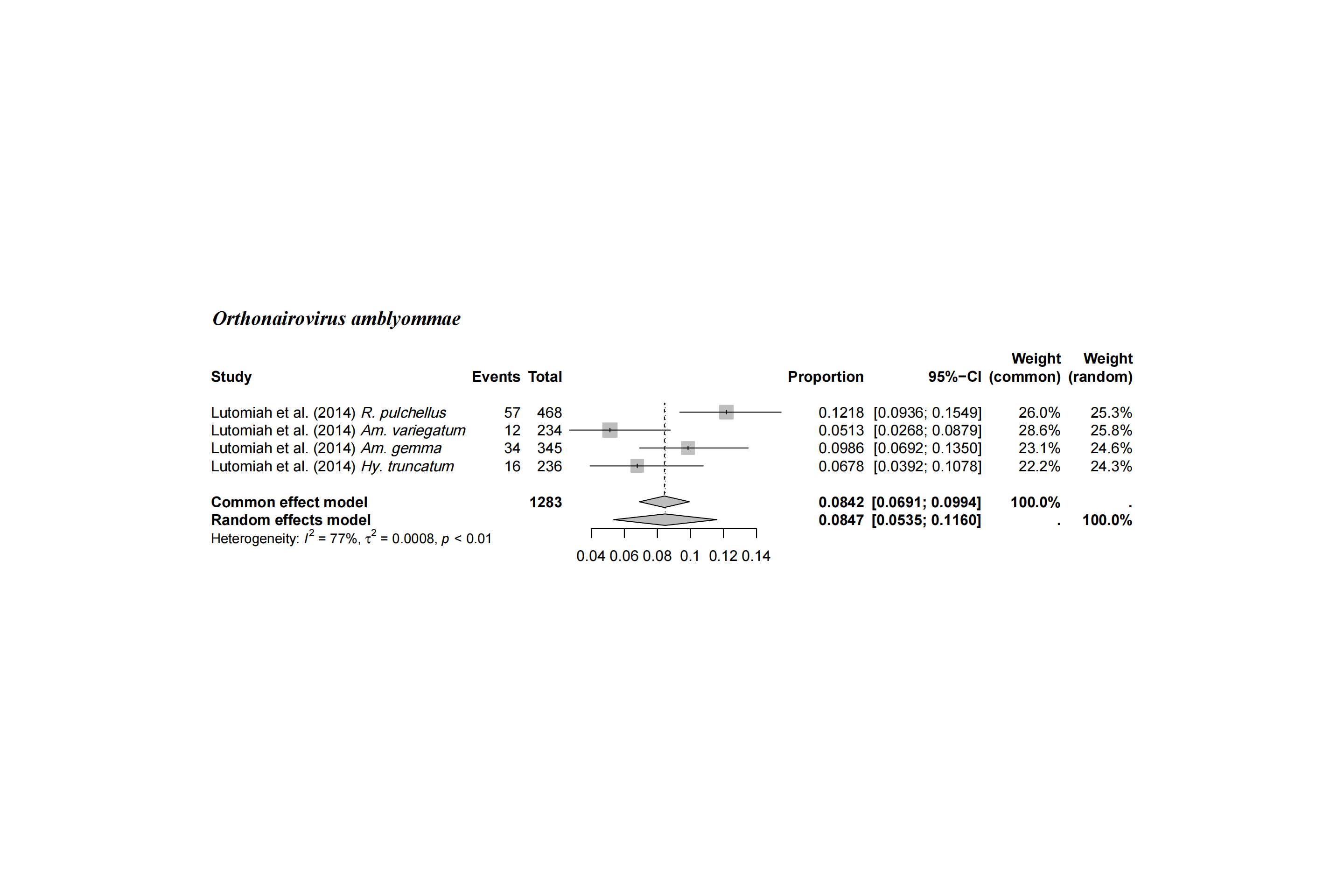


**9-3 Positive rate of *Orthobunyavirus bunyamweraense***


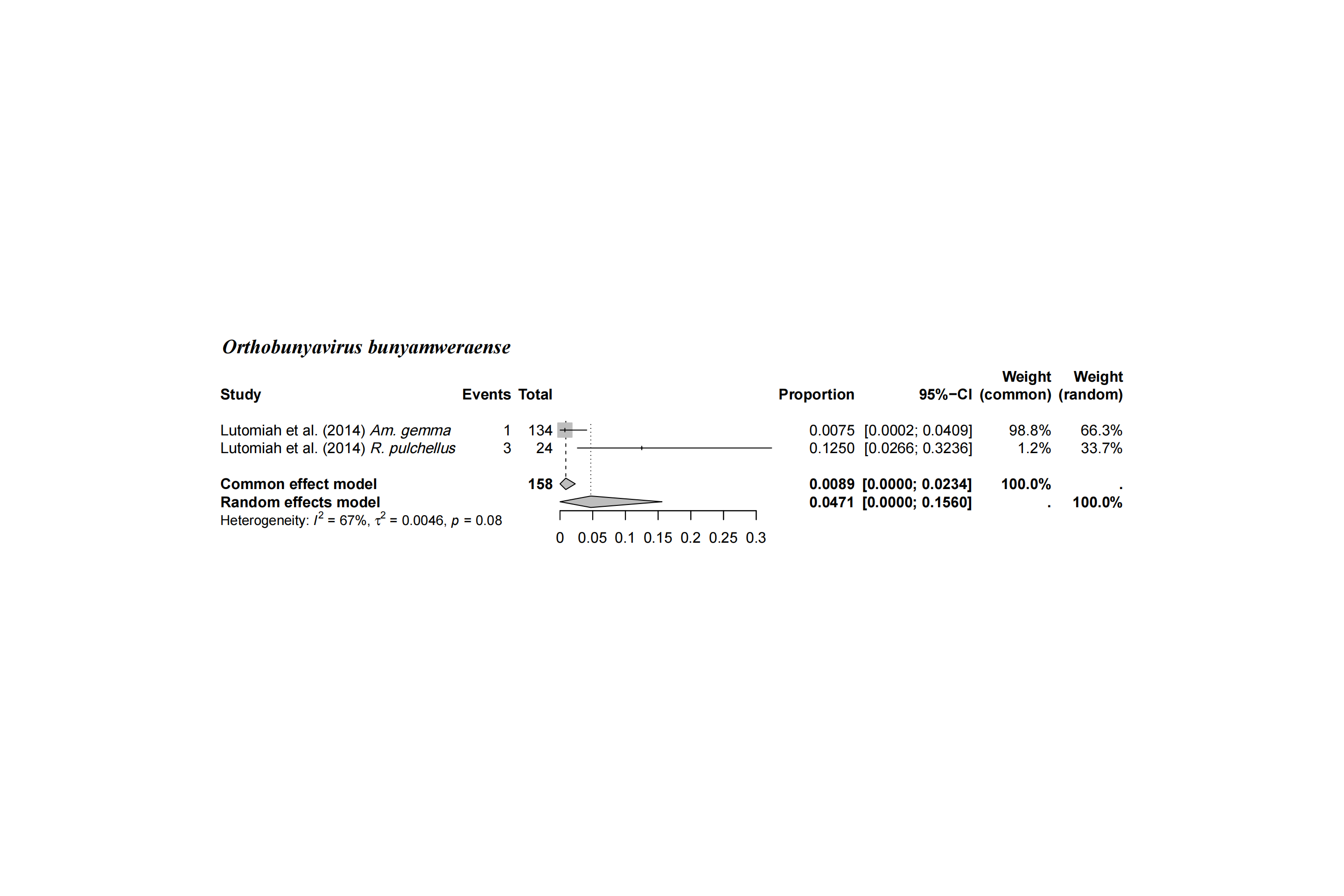


**9-4 Positive rate of *Orthonairovirus dugbeense***


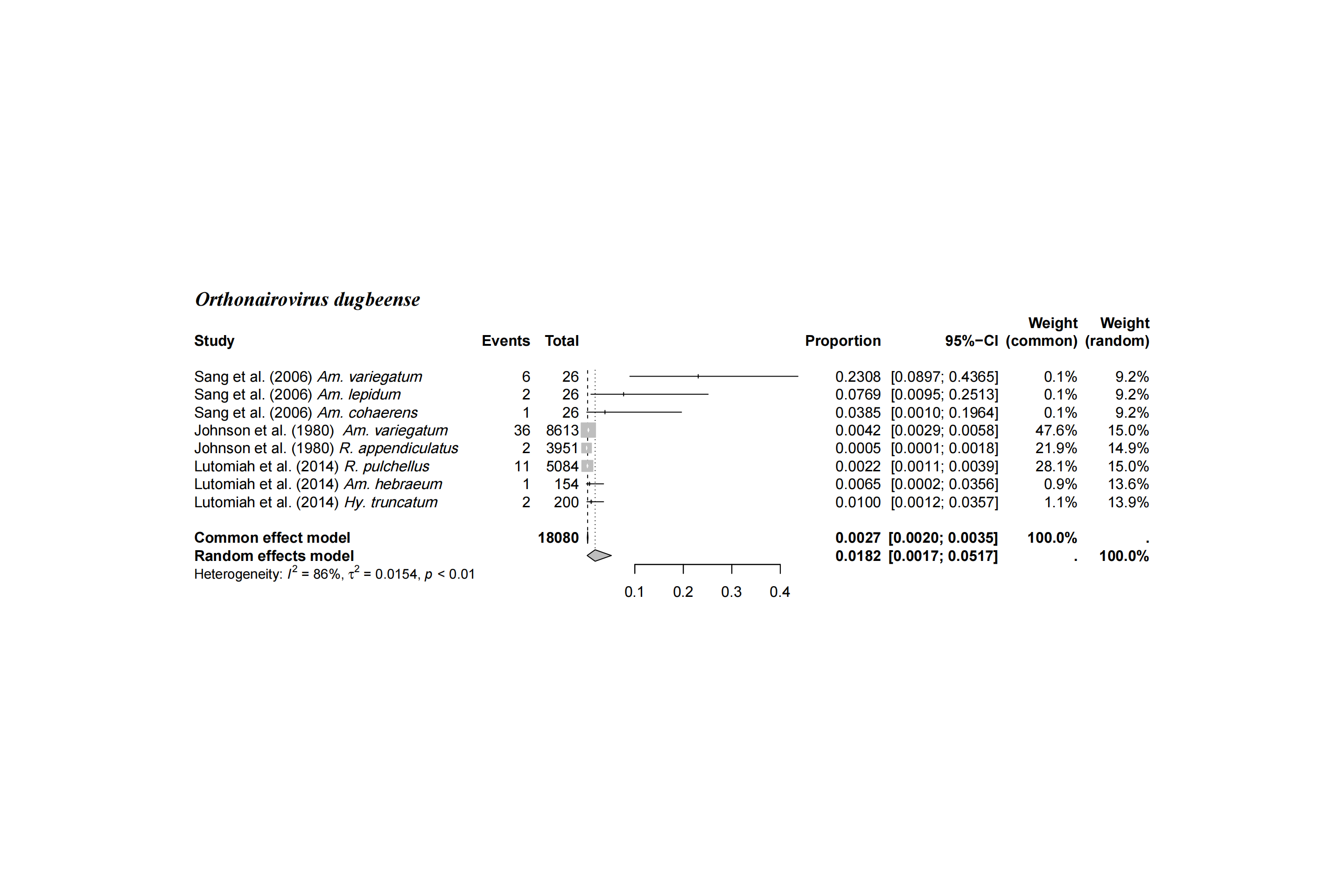


**9-5 Positive rate of *Orthonairovirus haemorrhagiae***


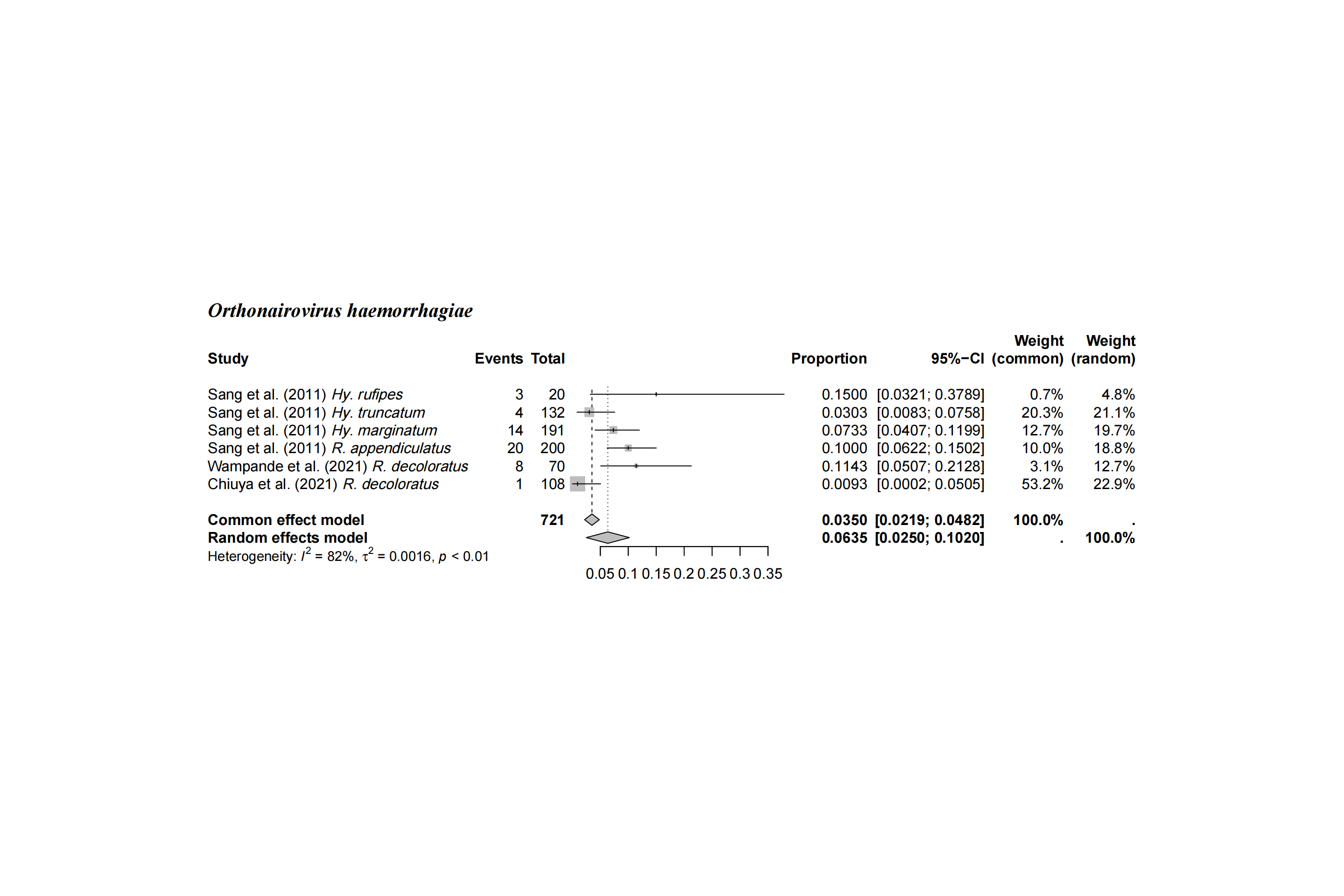


**9-6 Positive rate of *Orthonairovirus parahaemorrhagiae***


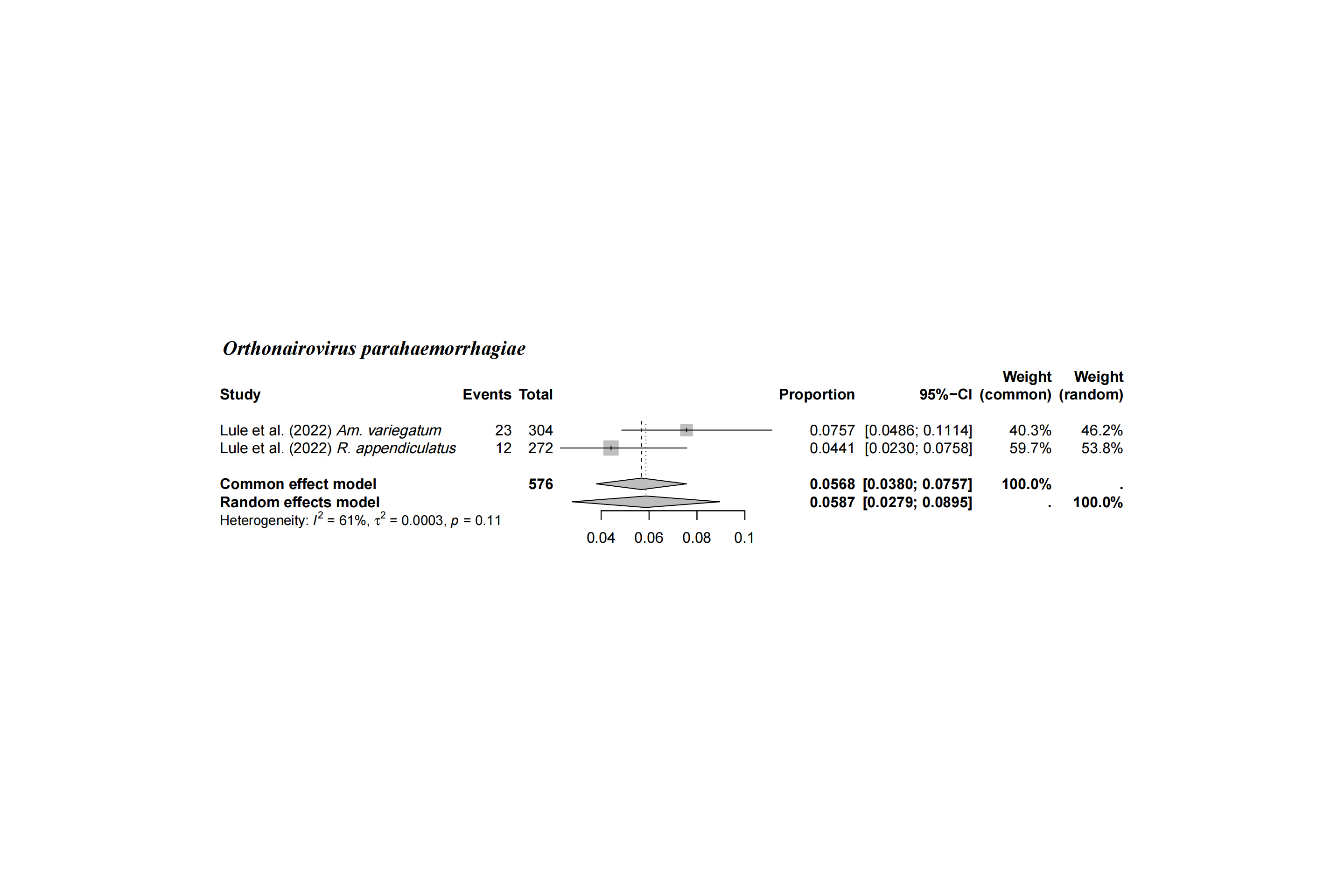


**9-7 Positive rate of *Thogotovirus thogotoense***

*
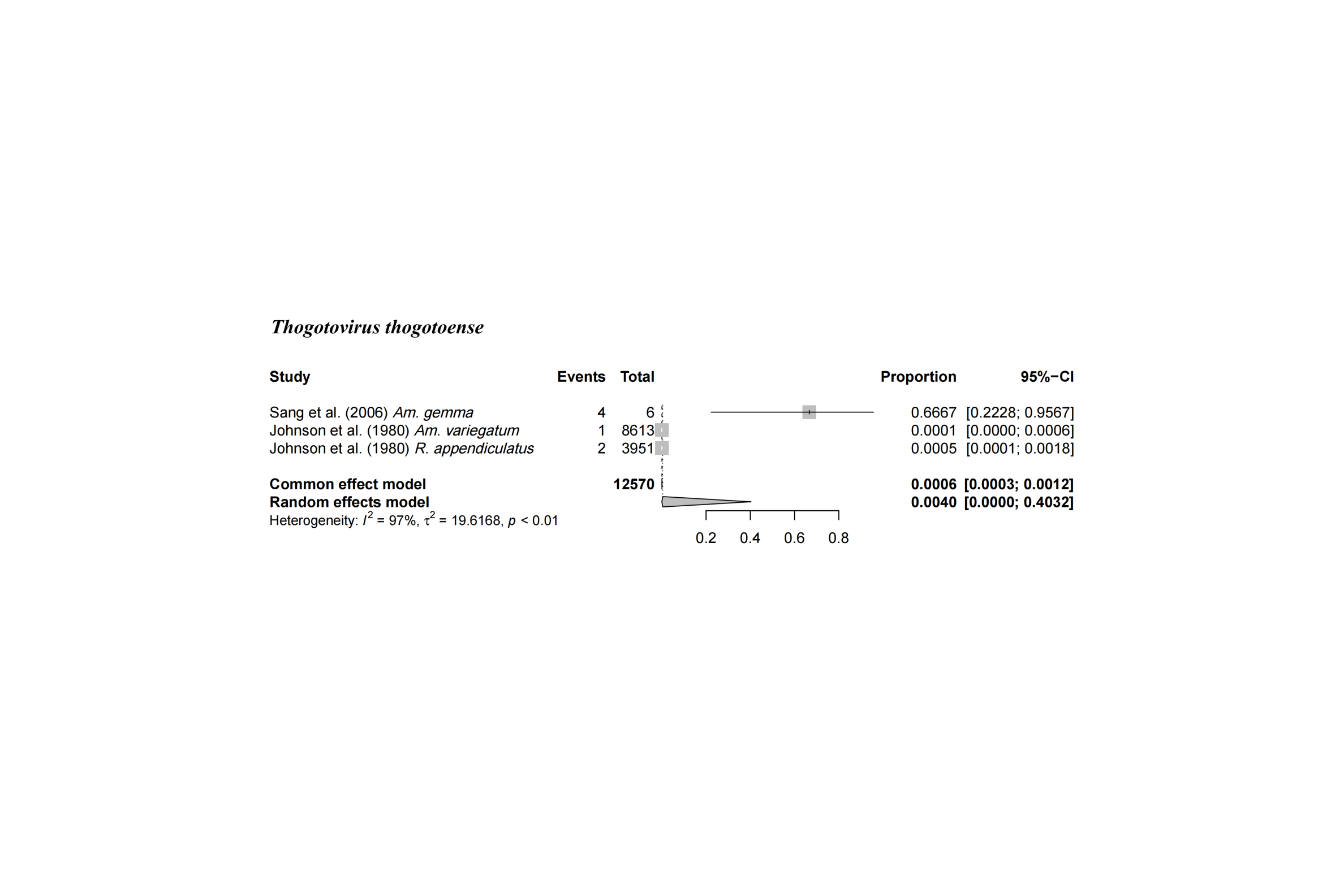
*

**9-8 Positive rate of Unclassified *Phenuiviridae***


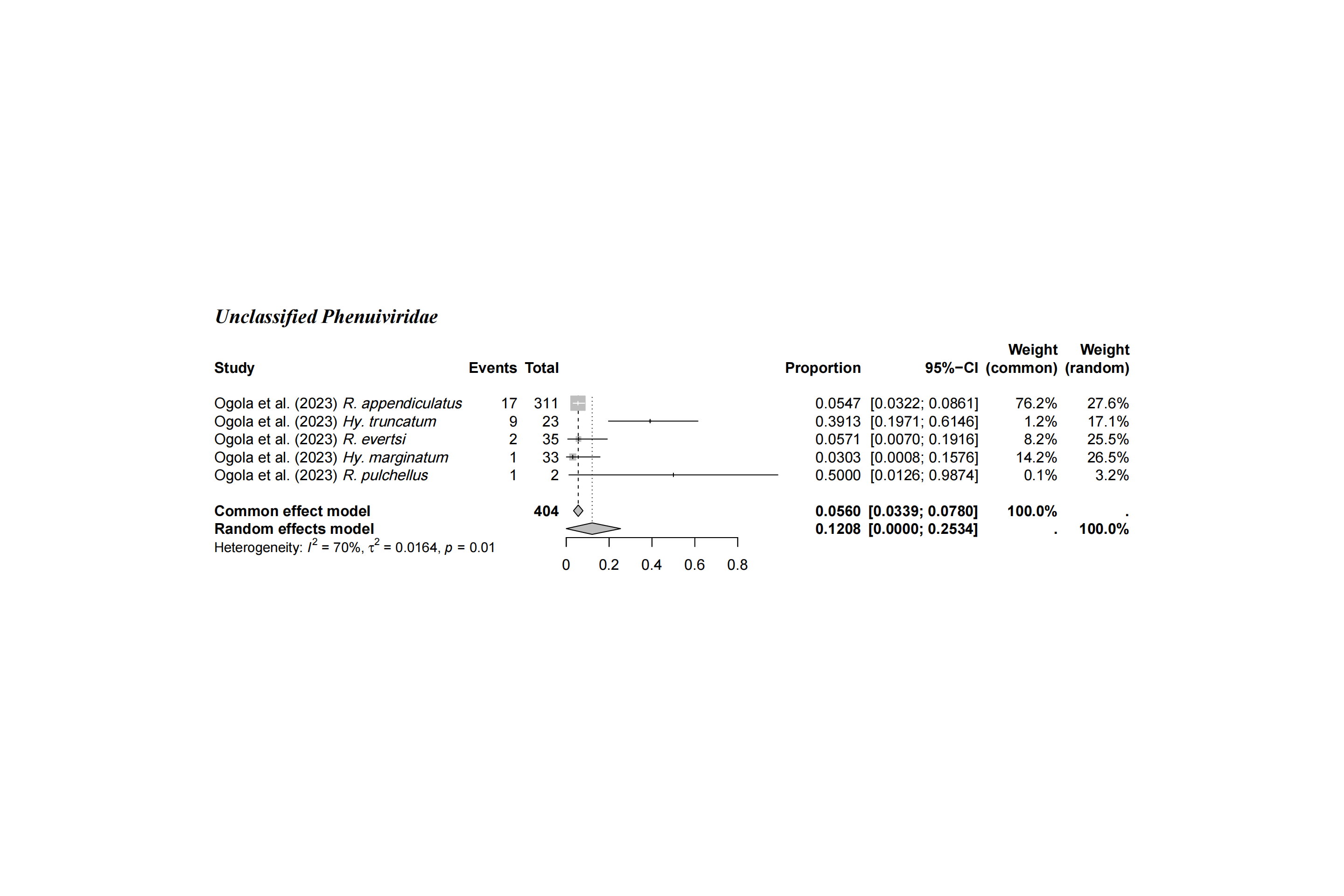


**9-9 Positive rate of *Anaplasma bovis***


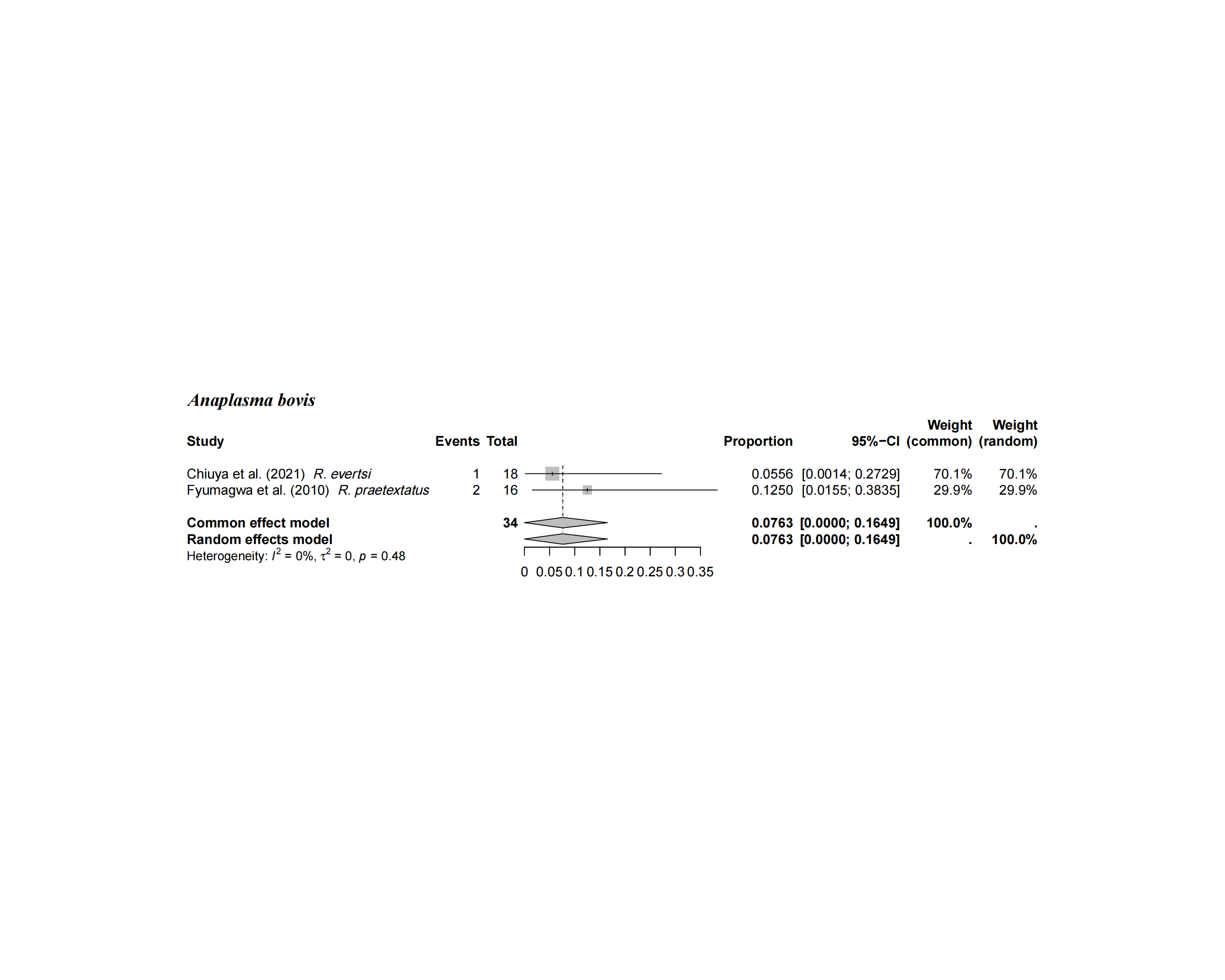


**9-10 Positive rate of *Coxiella burnetii***


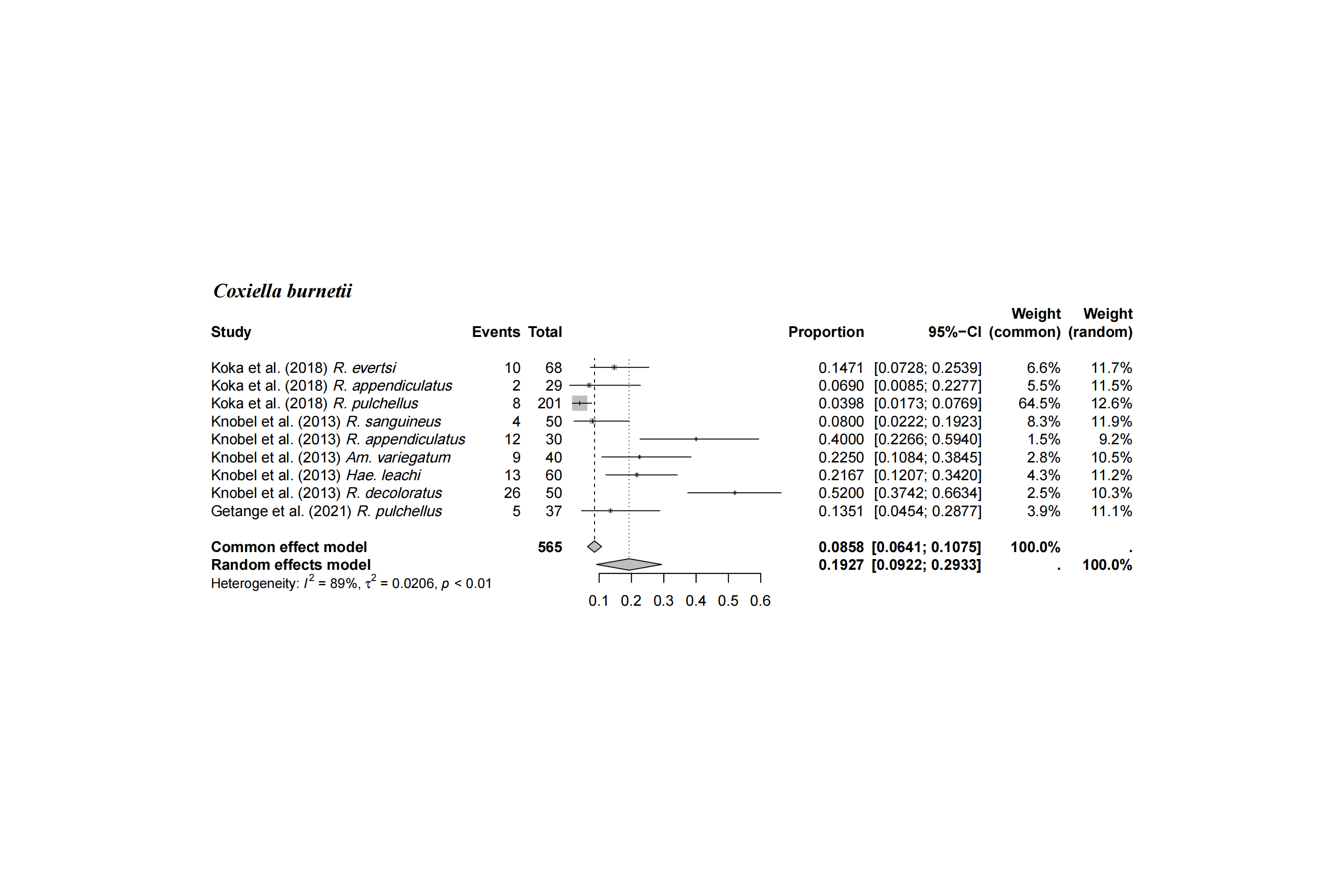


**9-11 Positive rate of *Candidatus Ehrlichia regneryi***


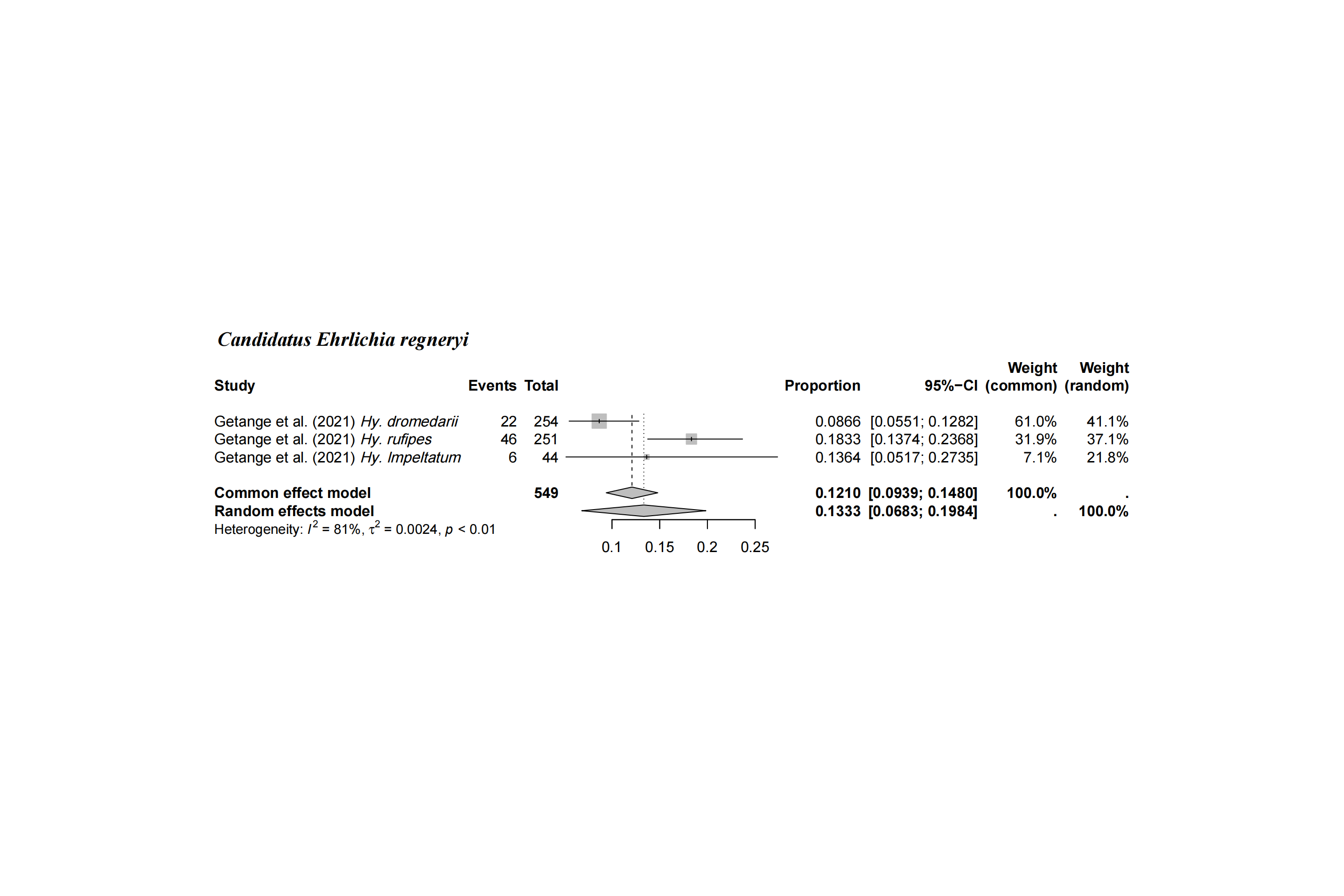


**9-12 Positive rate of *Candidatus Rickettsia africaseptentrionalis***


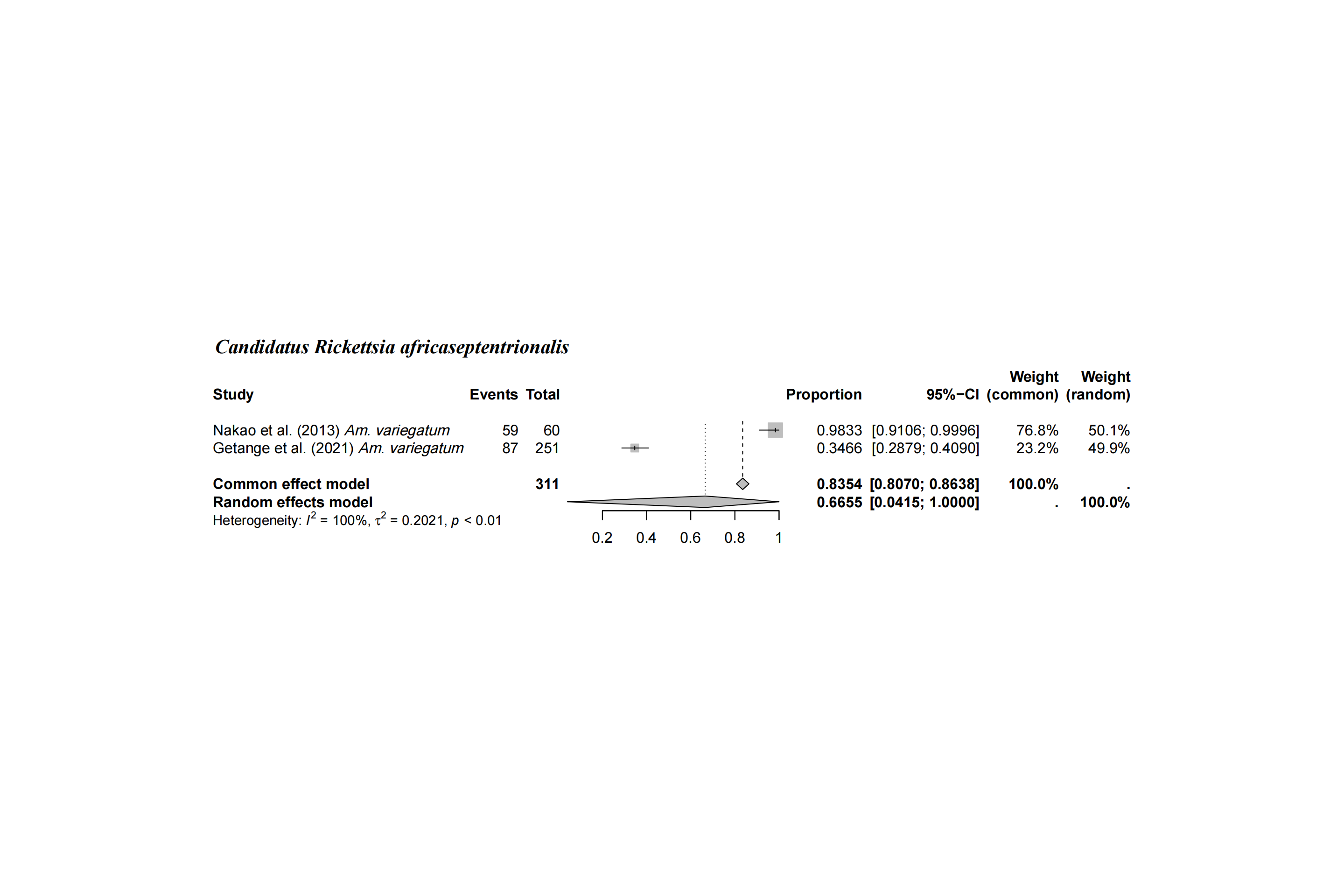


**9-13 Positive rate of *Rickettsia aeschlimannii***


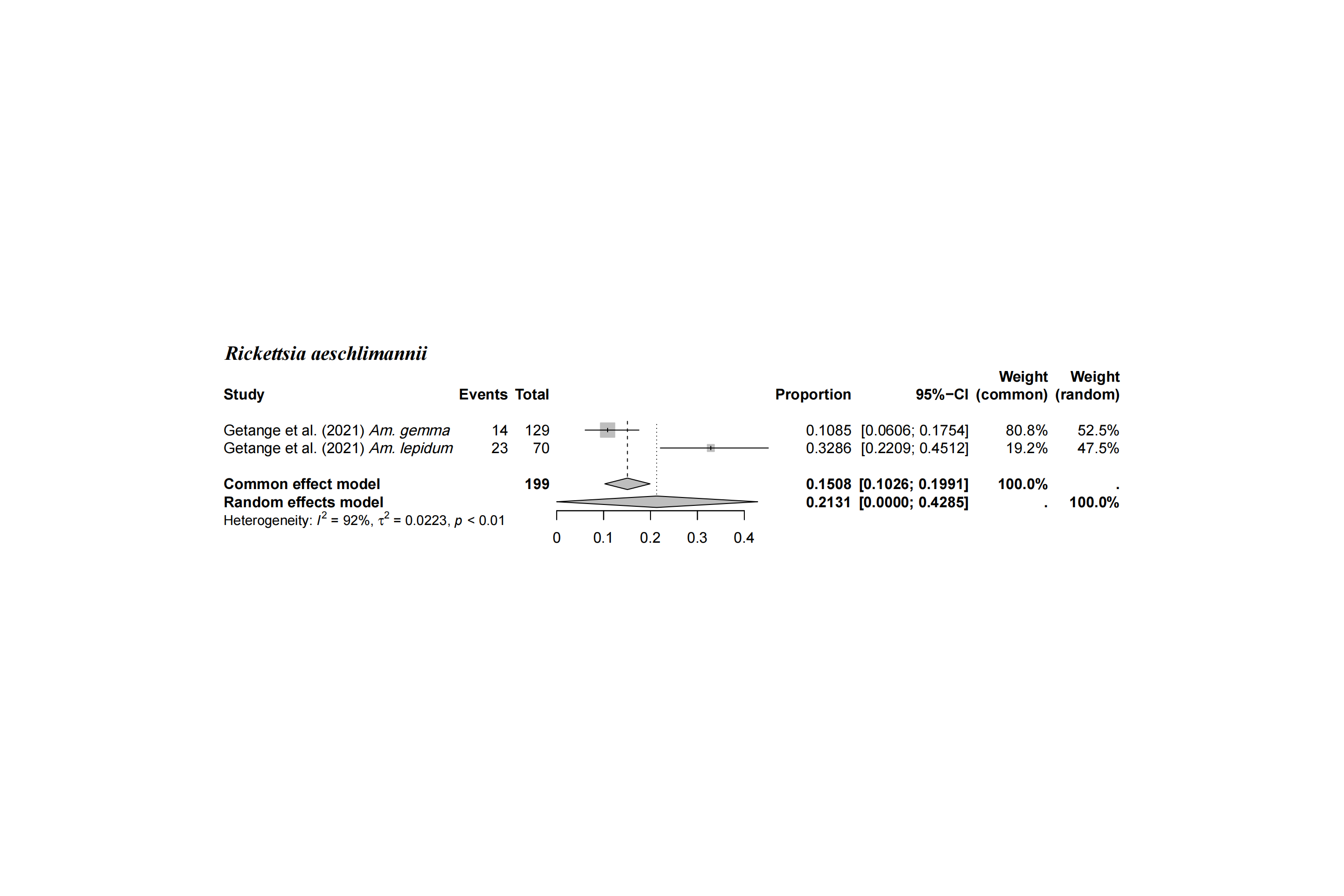


**9-14 Positive rate of *Rickettsia africae***


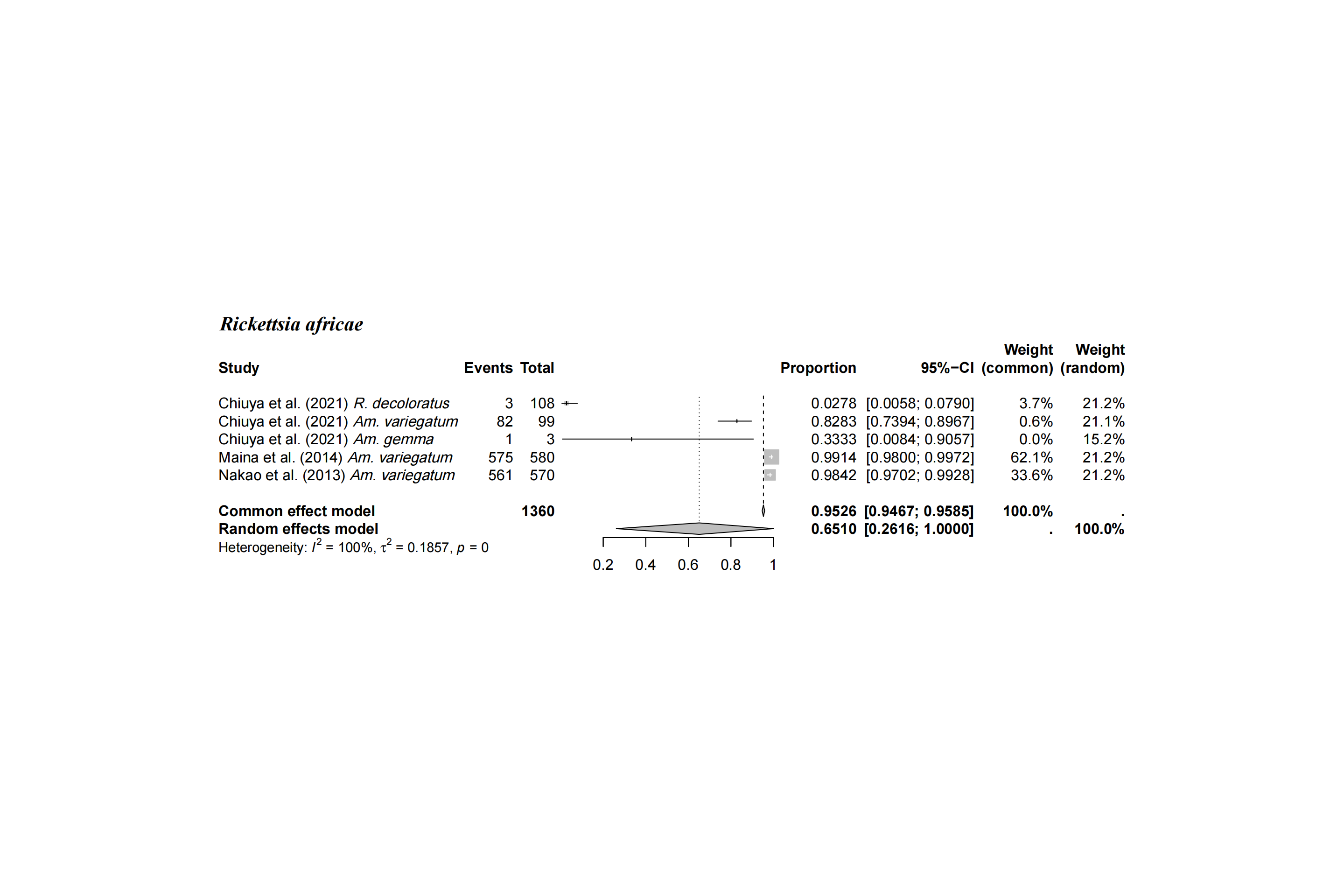


**9-15 Positive rate of *Theileria equi***


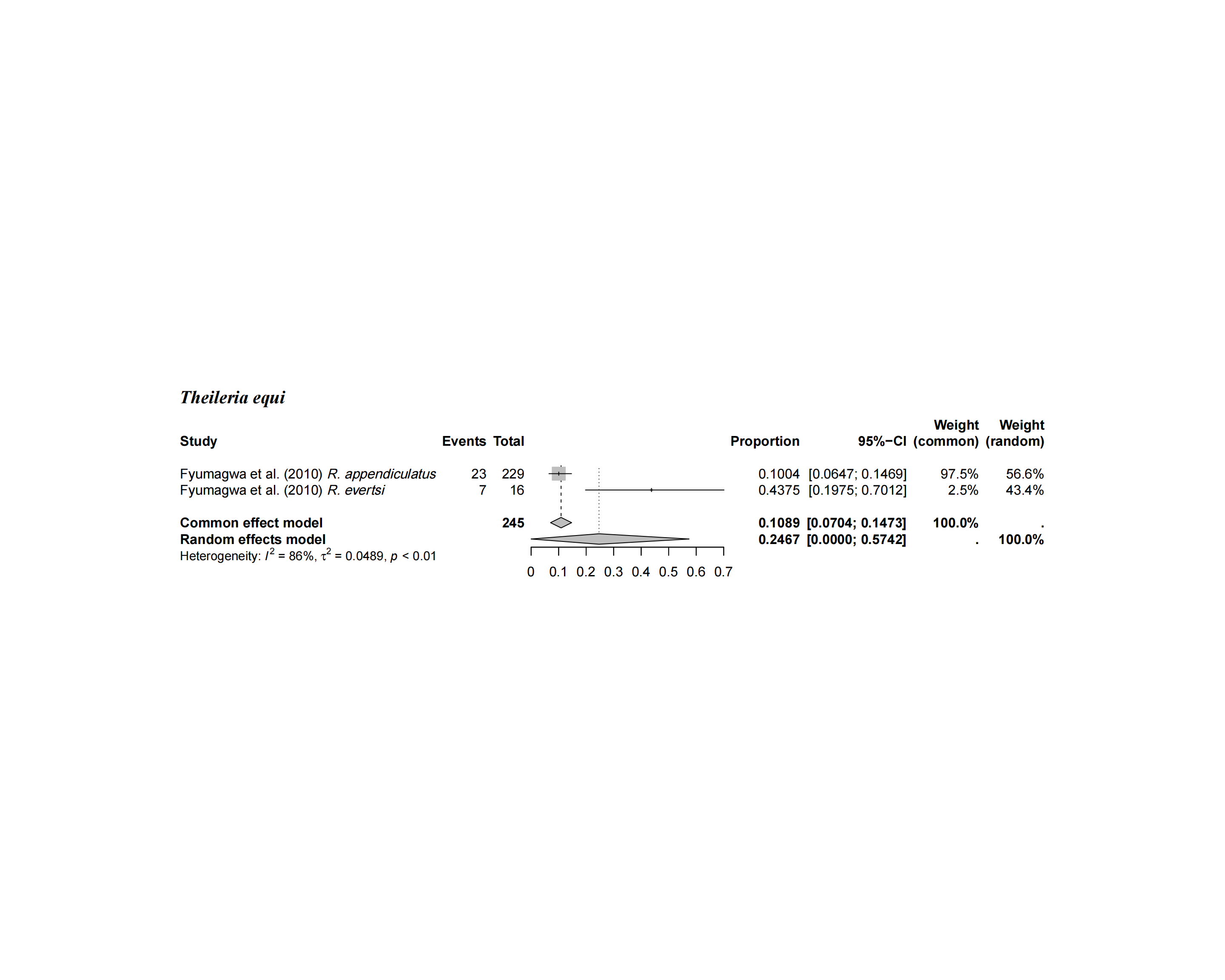


**9-16 Positive rate of *Theileria parva***

***
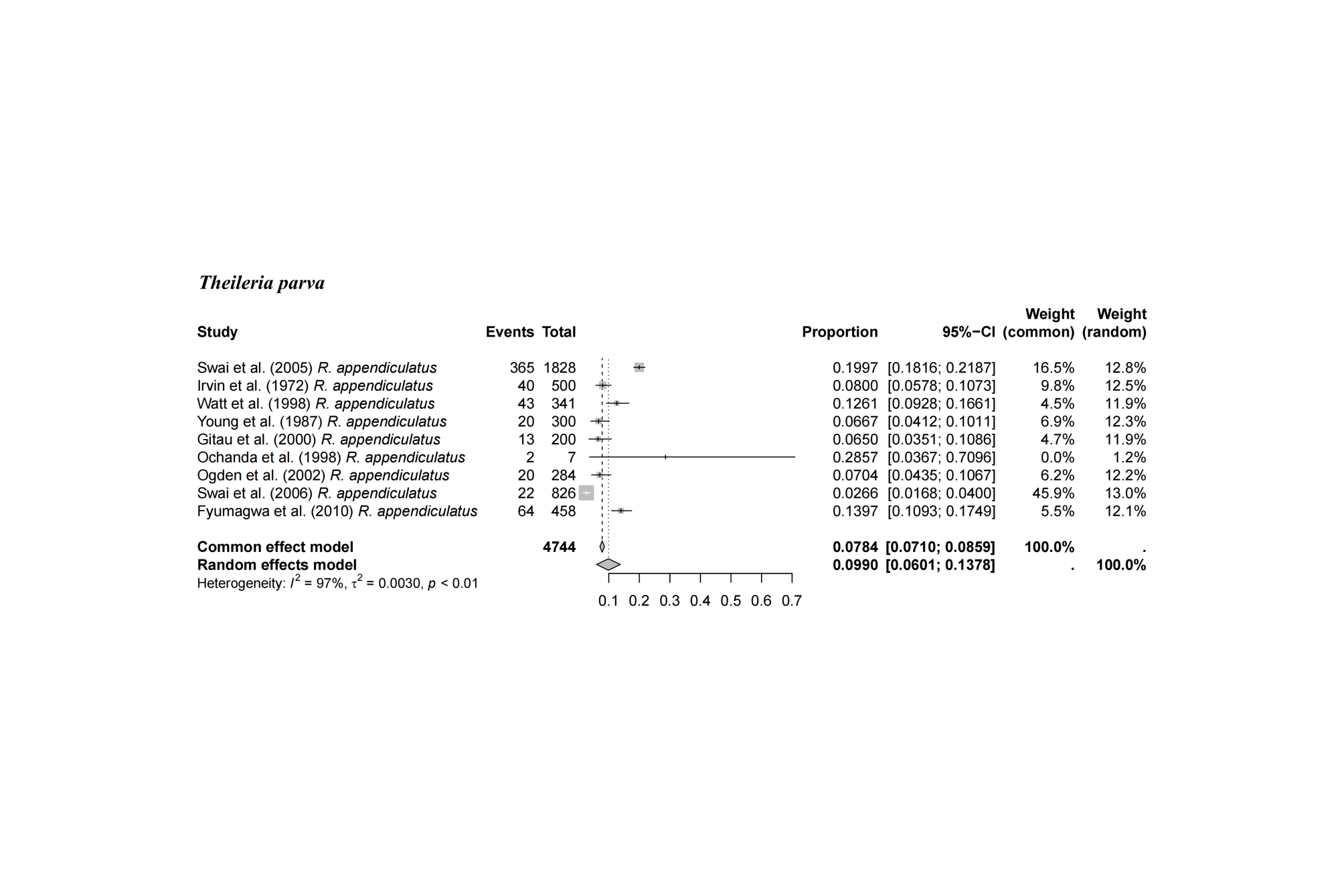
***

# **Table S4. Relative contributions of environmental and meteorological variables to the MaxEnt model**

| **Variable** | **Percent contribution** | **Permutation importance** |
| --- | --- | --- |
| Elevation | 48.7 | 16.2 |
| bio11 | 23.1 | 26.2 |
| Percent Tree Cover | 7 | 3.2 |
| bio7 | 5 | 12.4 |
| bio16 | 4.5 | 8.7 |
| bio18 | 4.1 | 5.8 |
| bio14 | 2.8 | 13.3 |
| bio19 | 2.4 | 12 |
| Land Cover | 2.3 | 2.2 |

# **Fig. S10. Receiver Operating Characteristic (ROC) curve of the best MCP model for *Amblyomma variegatum***

The Maxent model for ***Amblyomma variegatum*** is represented by the Receiver Operating

Characteristic (ROC) curve. This curve is an average of the results obtained from 25 replicate runs. The specificity is determined based on the predicted area.


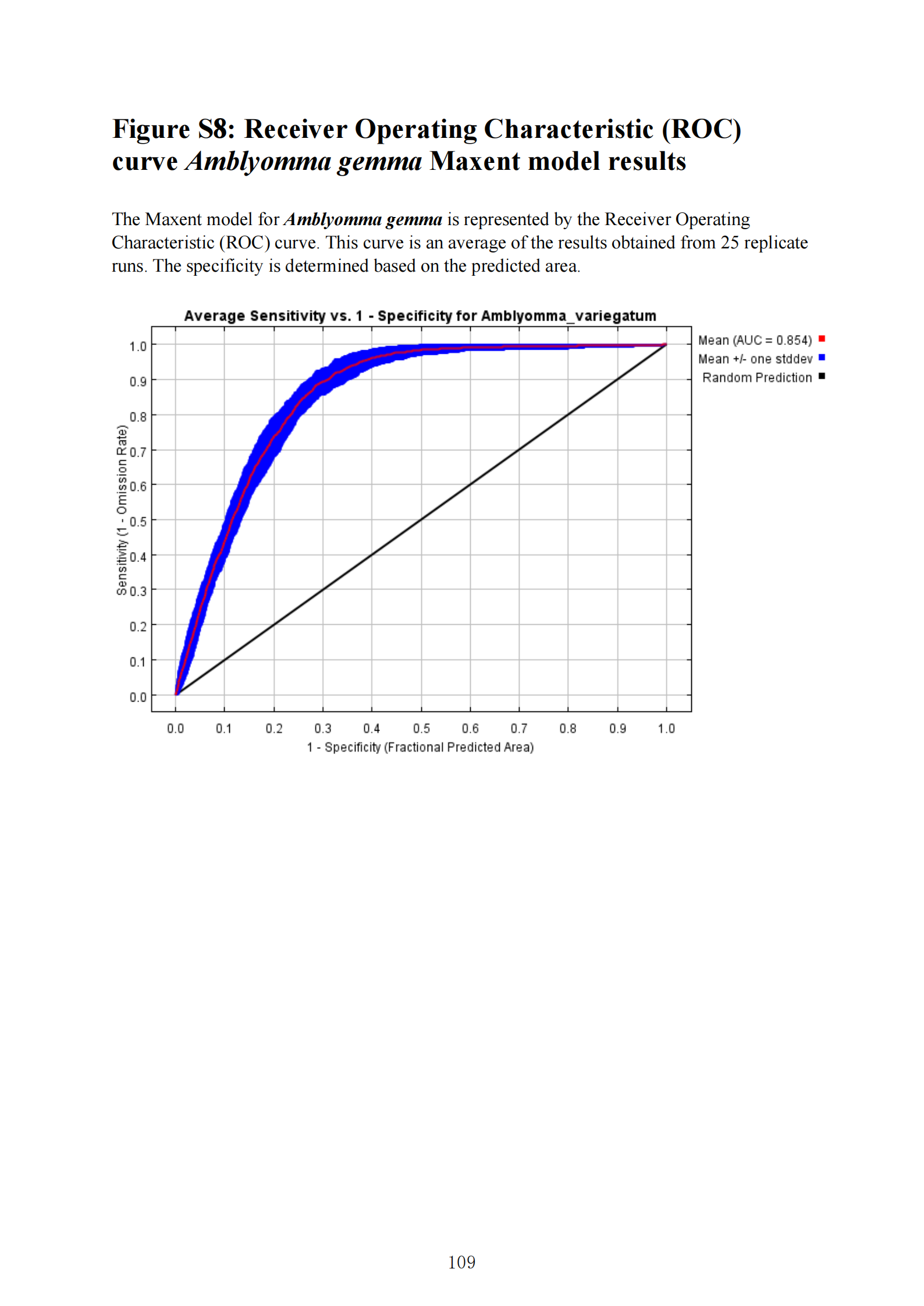


# **Fig. S11. Jackknife plots of MaxEnt model for *Amblyomma variegatum* prediction.**

1. training gain plot; B. AUC; C. test gain plot


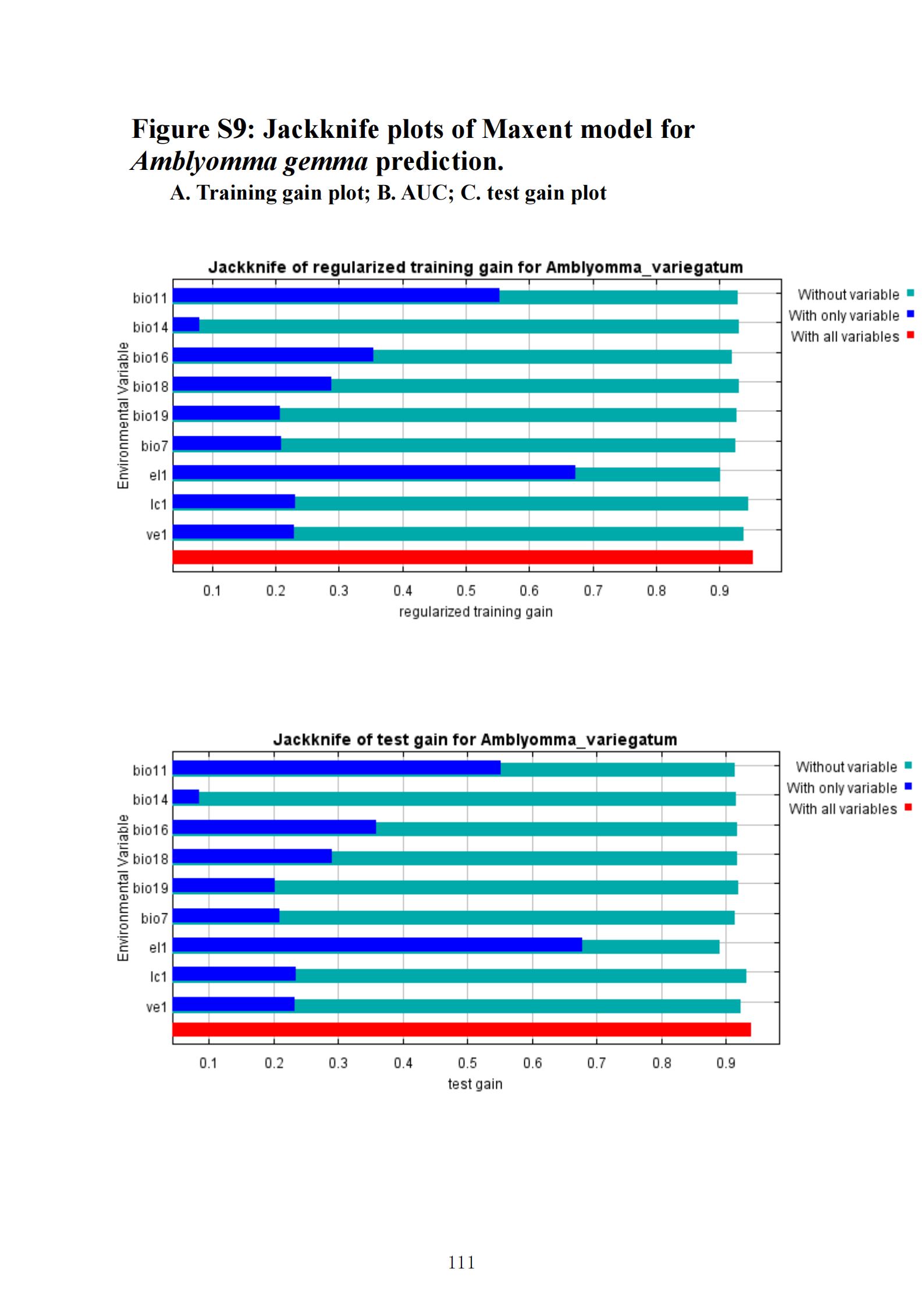


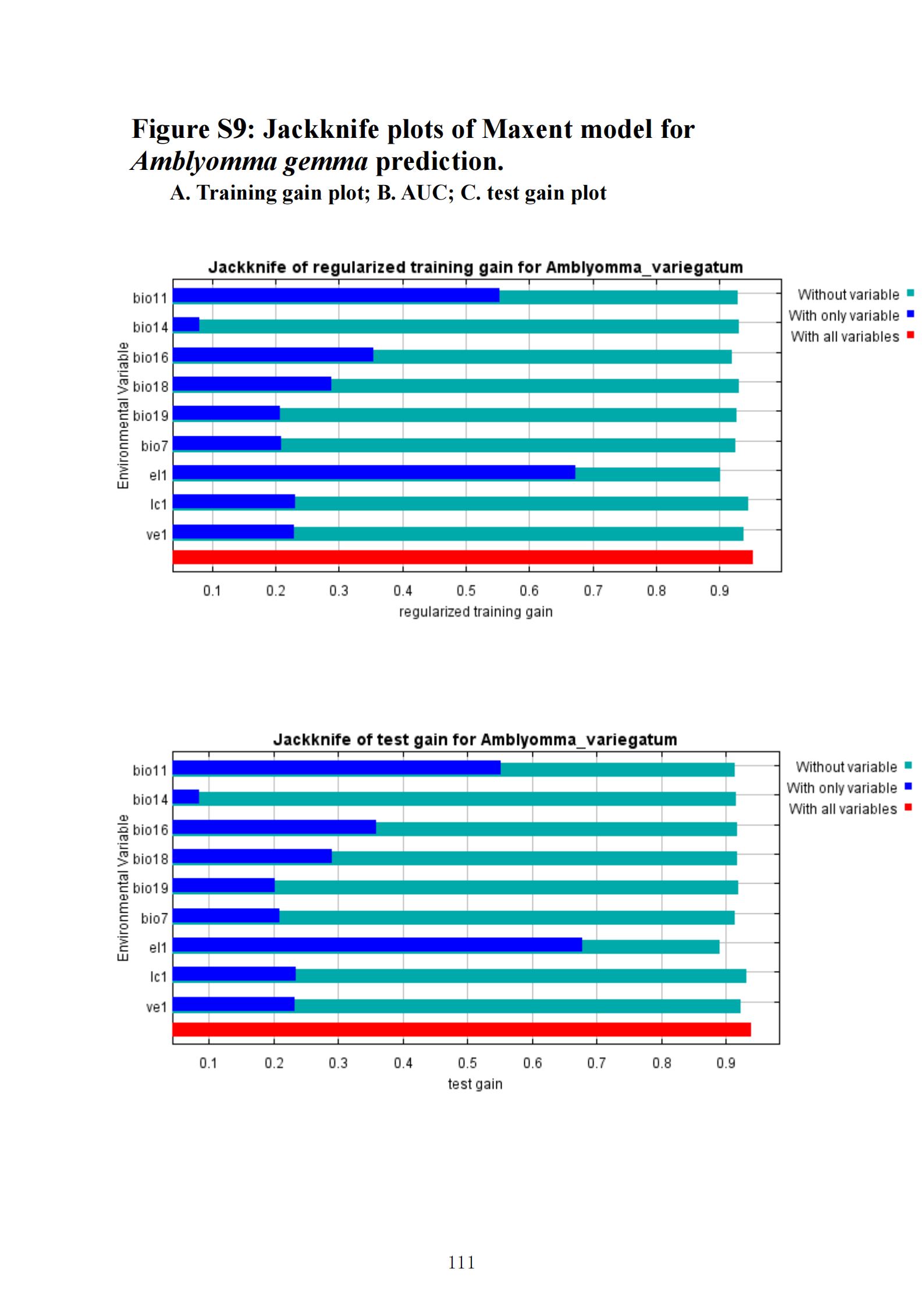


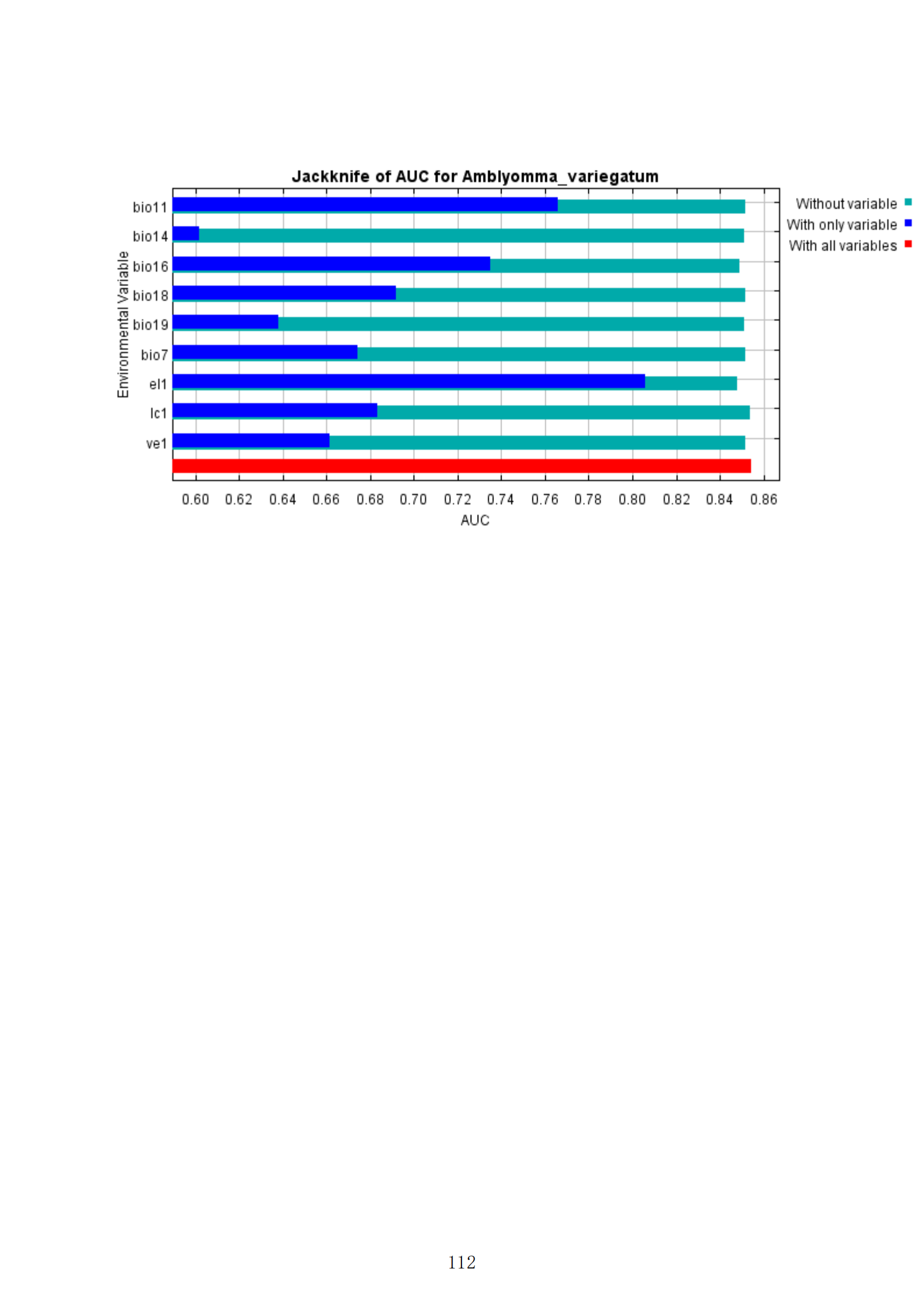


# **Fig. S12. Response curves of environmental variables indicate the likelihood of *Amblyomma variegatum* being present.**

These curves illustrate the average outcomes from 25 repeated Maxent model runs,

represented by the red line. The blue shaded areas show the standard deviation, highlighting the variability across these runs.


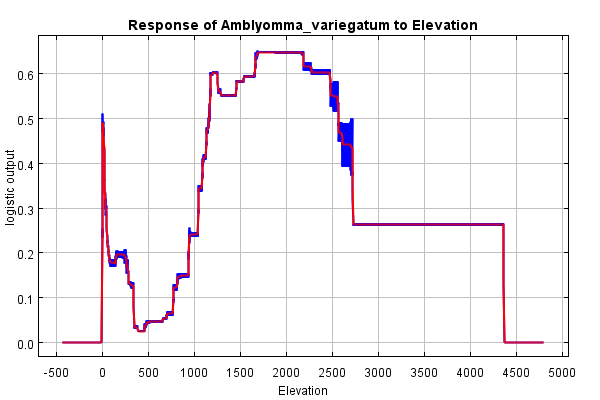

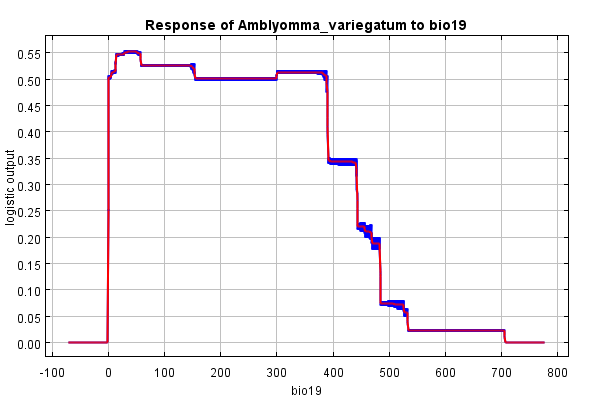

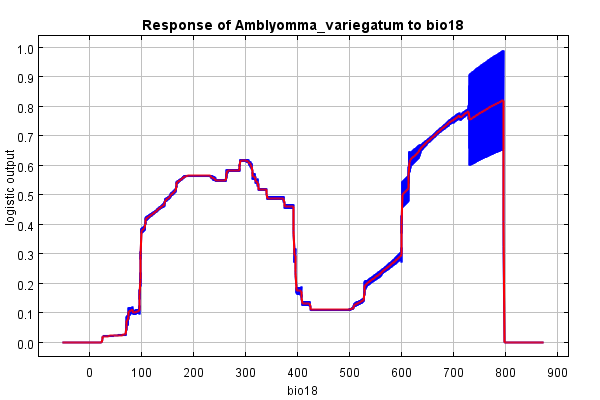

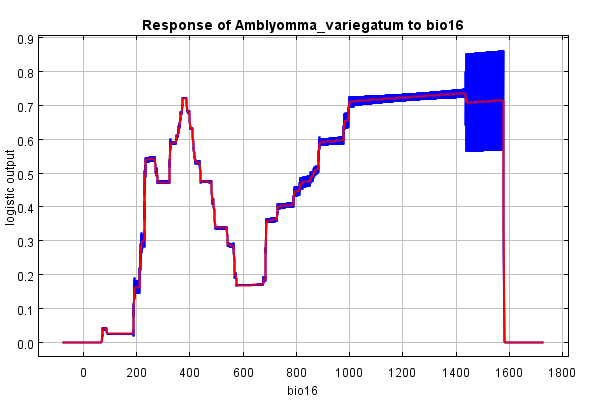

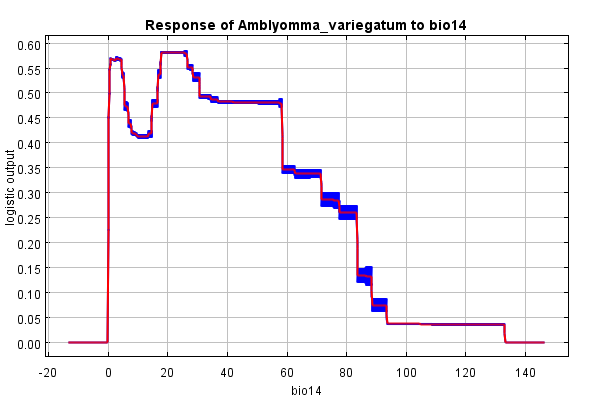

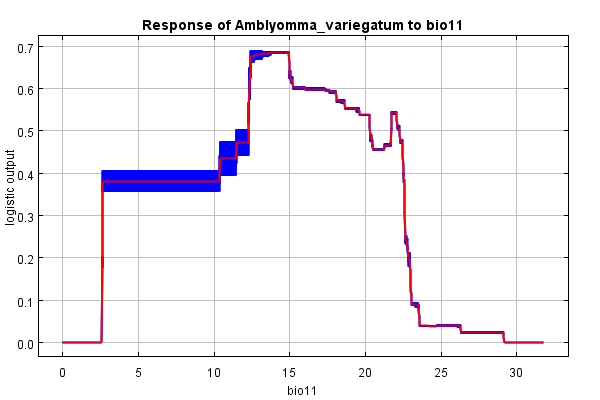

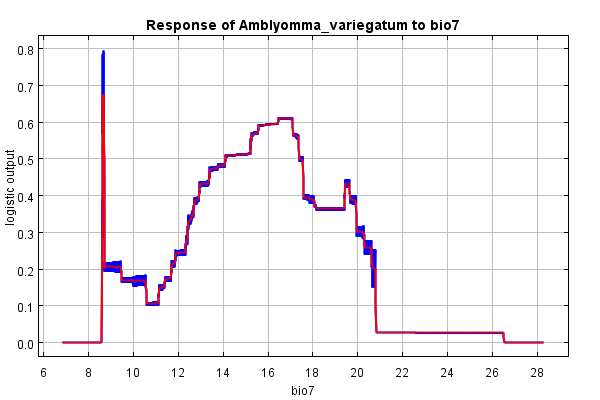

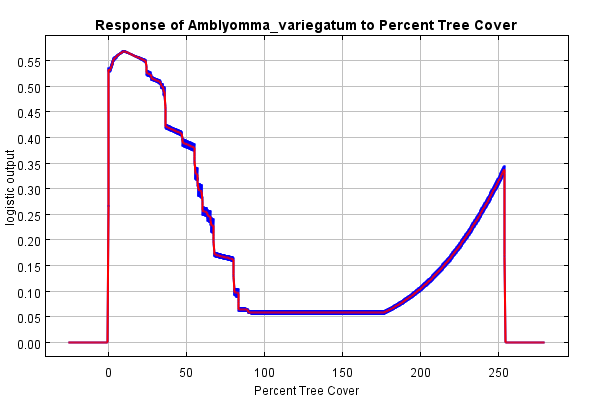


# **Fig. S13. Receiver Operating Characteristic (ROC) curve of the best MCP model for *Haemaphysalis leachi***

The Maxent model for ***Haemaphysalis leachi*** is represented by the Receiver Operating Characteristic (ROC) curve. This curve is an average of the results obtained from 25 replicate runs. The specificity is determined based on the predicted area.


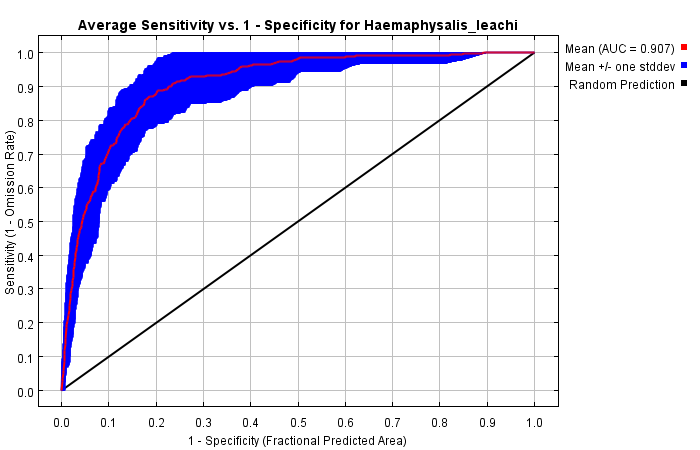


# **Table S5. Relative contributions of environmental and meteorological variables to the MaxEnt model**

| **Variable** | **Percent contribution** | **Permutation importance** |
| --- | --- | --- |
| Elevation | 27.6 | 9.1 |
| bio8 | 22.7 | 0.3 |
| bio11 | 11.4 | 2.2 |
| bio17 | 10.8 | 44.7 |
| bio4 | 5.3 | 4.1 |
| bio19 | 5 | 7.1 |
| Percent Tree Cover | 4.1 | 1.6 |
| Slope | 3.7 | 0.9 |
| Land Cover | 3 | 1.5 |
| bio18 | 2.4 | 5.7 |
| bio15 | 1.9 | 21.3 |
| bio2 | 1.5 | 0.9 |
| bio13 | 0.7 | 0.6 |

# **Fig. S14. Jackknife plots of MaxEnt model for *Haemaphysalis leachi* prediction.**

A. training gain plot; B. AUC; C. test gain plot


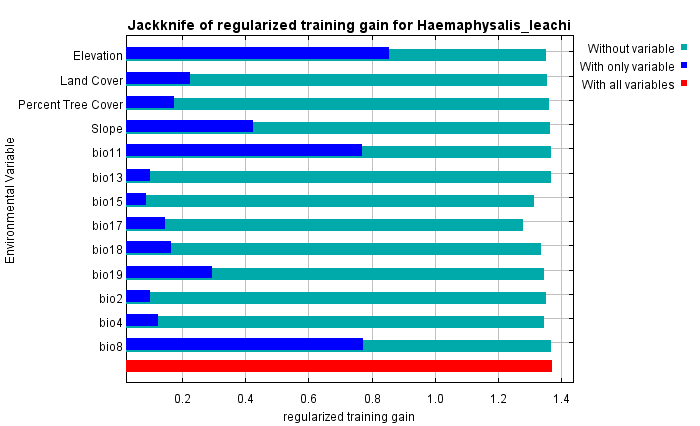

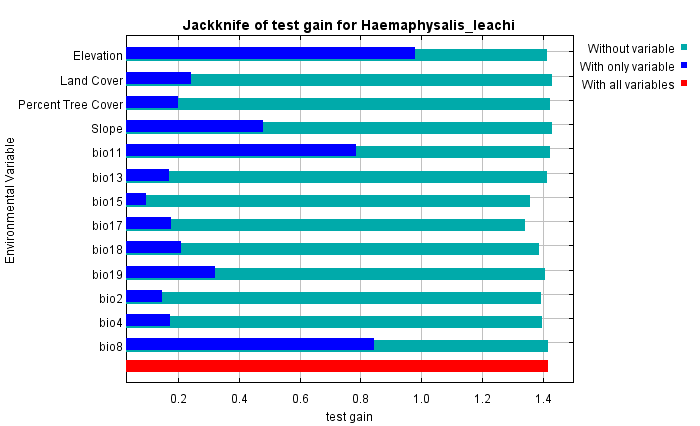

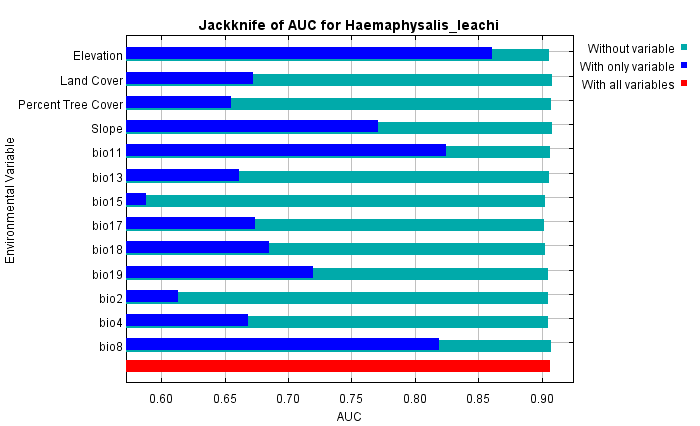


# **Fig. S15. Response curves of environmental variables indicate the likelihood of *Haemaphysalis leachi* being present.**

These curves illustrate the average outcomes from 25 repeated Maxent model runs,

represented by the red line. The blue shaded areas show the standard deviation, highlighting the variability across these runs.


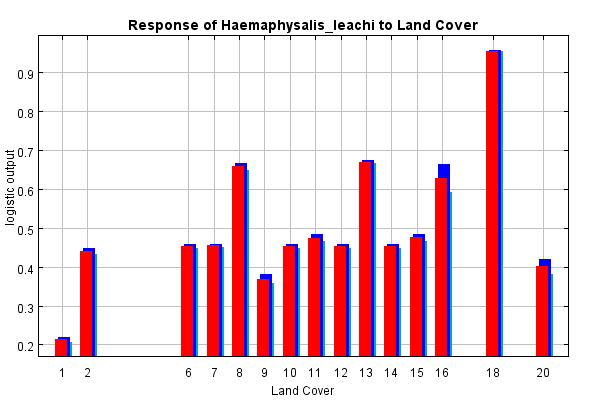

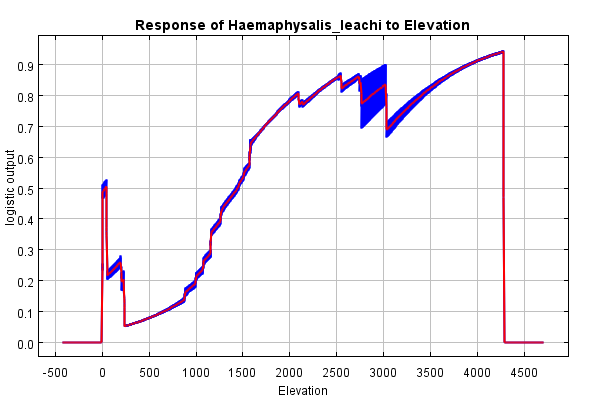

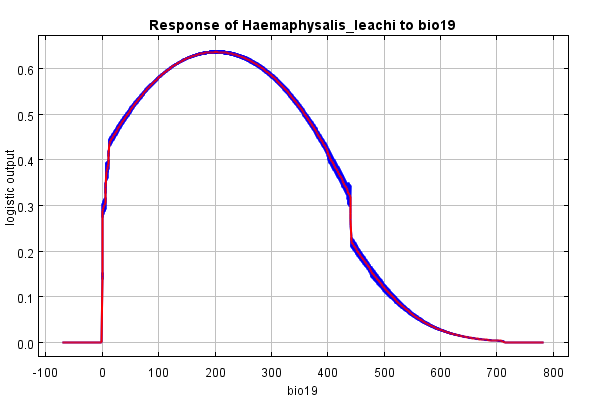

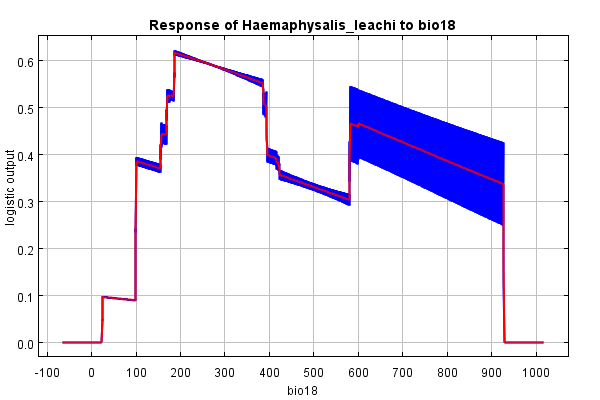

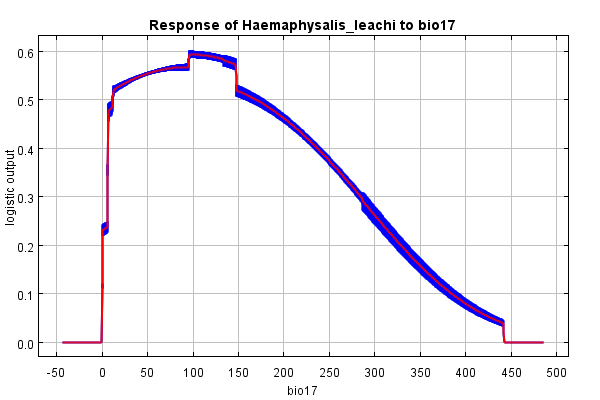

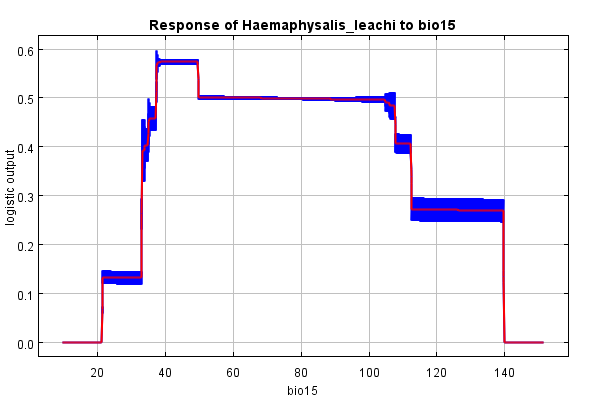

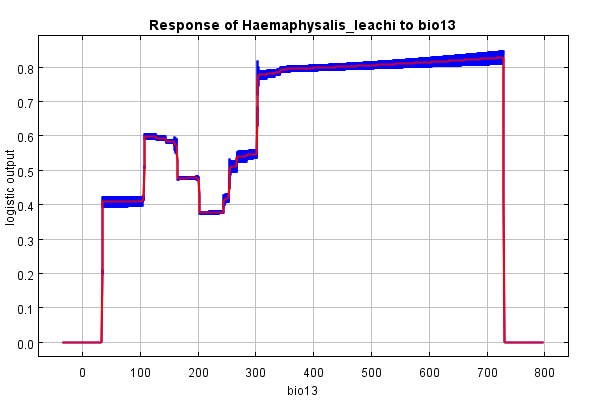

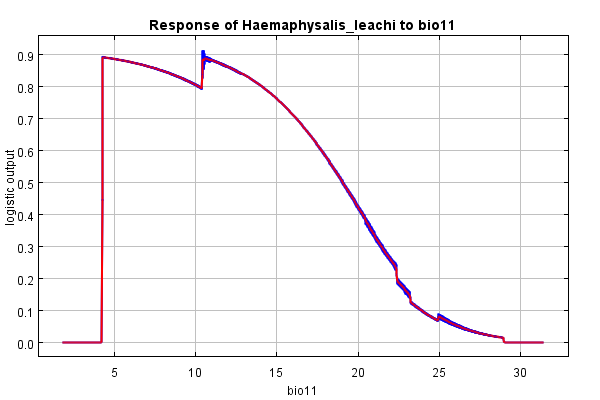

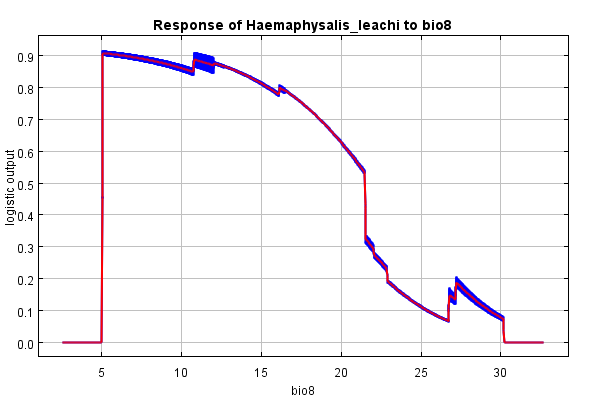

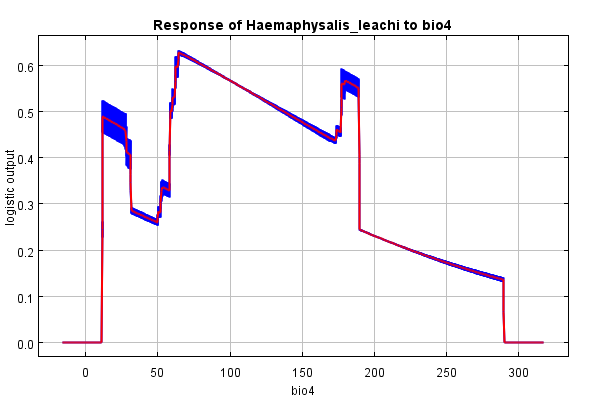

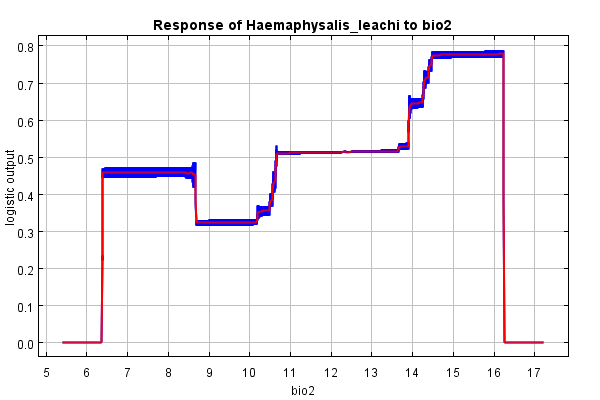

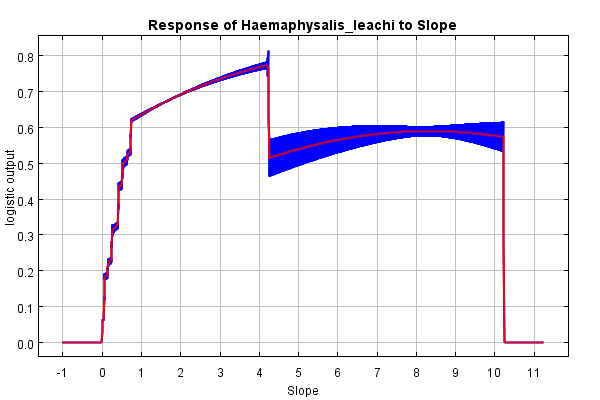

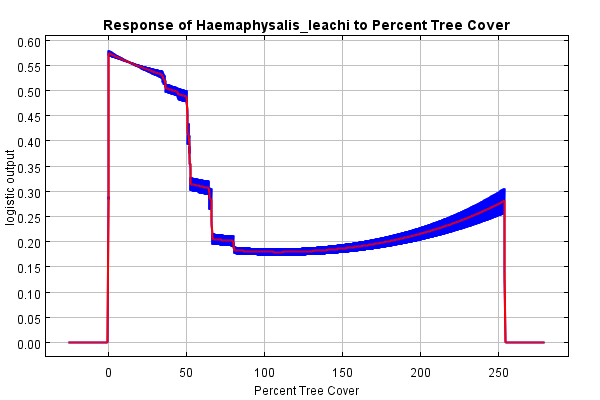


# **Fig. S16. Receiver Operating Characteristic (ROC) curve of the best MCP model for *Hyalomma truncatum***

The Maxent model for ***Hyalomma truncatum*** is represented by the Receiver Operating Characteristic (ROC) curve. This curve is an average of the results obtained from 25 replicate runs. The specificity is determined based on the predicted area.


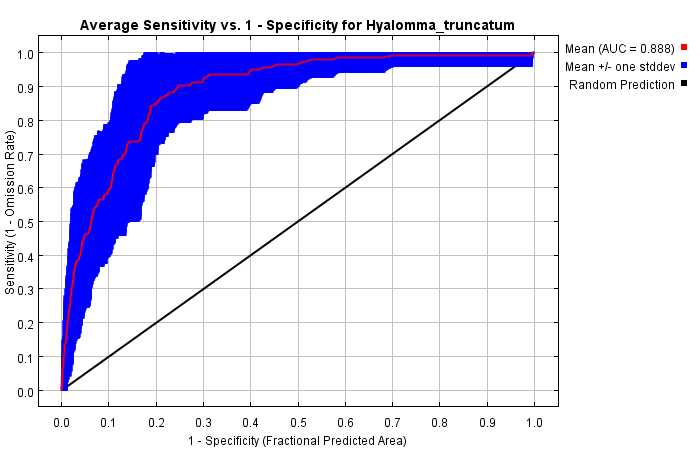


# **Table S6. Relative contributions of environmental and meteorological variables to the MaxEnt model**

| Variable | Percent contribution | Permutation importance |
| --- | --- | --- |
| bio12 | 27.8 | 18.2 |
| bio1 | 23.7 | 11.8 |
| Percent Tree Cover | 13.6 | 8.5 |
| Elevation | 11.2 | 2.4 |
| bio3 | 4.6 | 4.2 |
| bio14 | 4 | 23.6 |
| bio2 | 3.9 | 4.1 |
| bio4 | 3.3 | 1.6 |
| bio19 | 2.7 | 1.8 |
| bio15 | 2.3 | 20.7 |
| Land Cover | 2.2 | 1.3 |
| bio18 | 0.7 | 1.7 |

# **Fig. S17. Jackknife plots of MaxEnt model for *Hyalomma truncatum* prediction.**

A. training gain plot; B. AUC; C. test gain plot


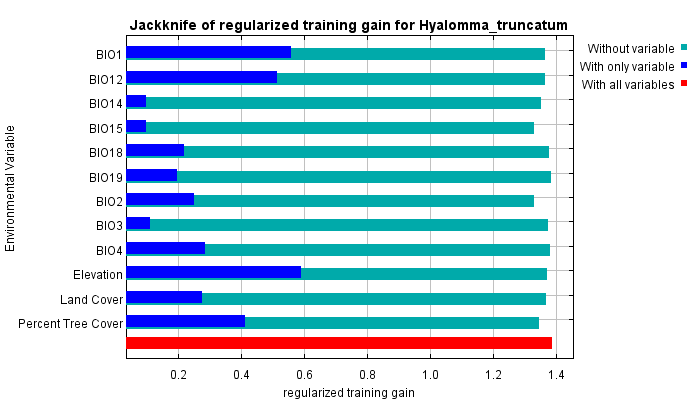

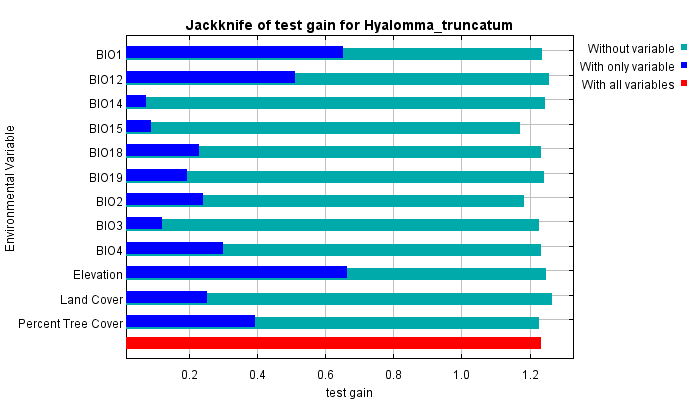

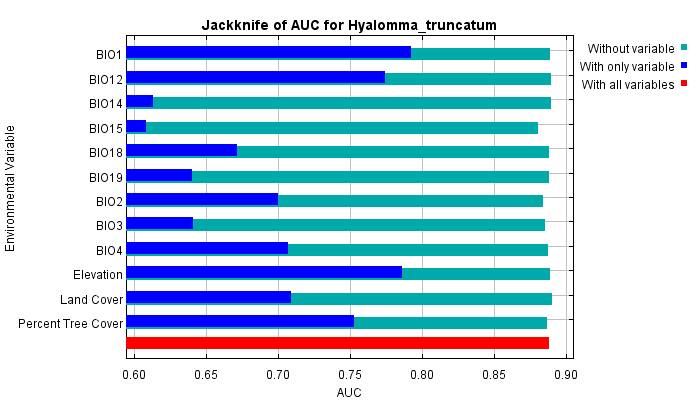


# **Fig. S18. Response curves of environmental variables indicate the likelihood of *Hyalomma truncatum* being present.**

These curves illustrate the average outcomes from 25 repeated Maxent model runs,

represented by the red line. The blue shaded areas show the standard deviation, highlighting the variability across these runs.


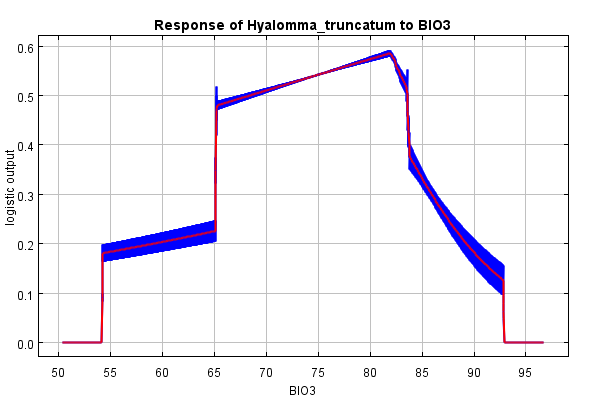

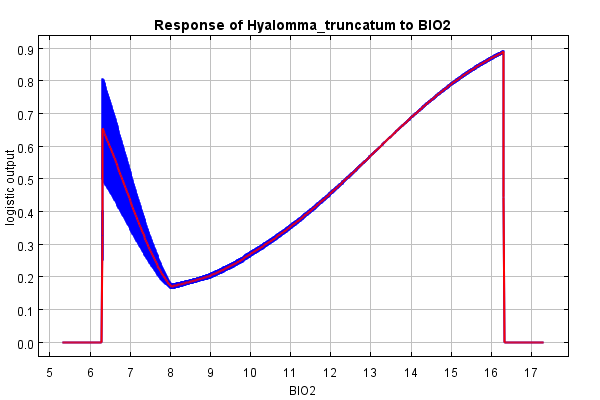

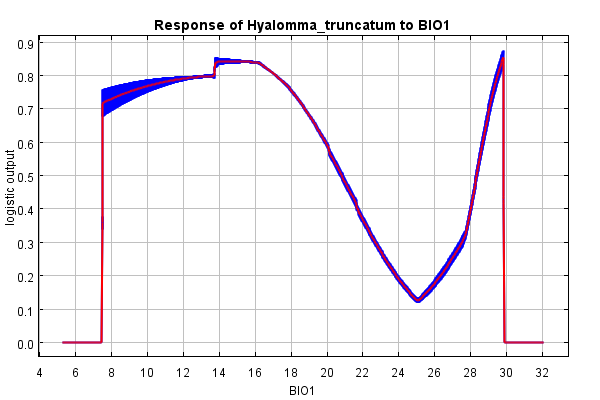

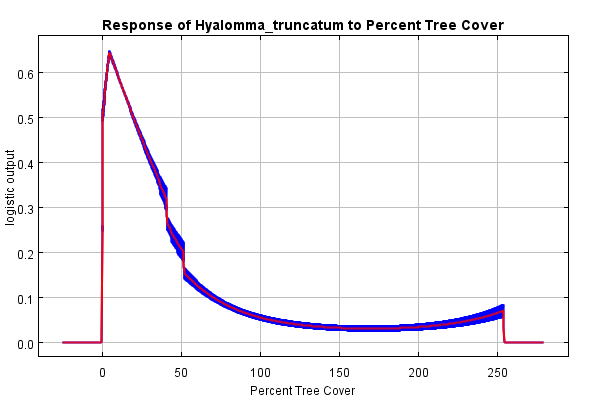

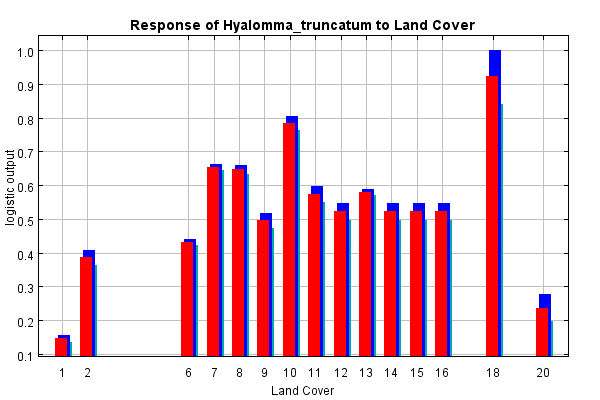

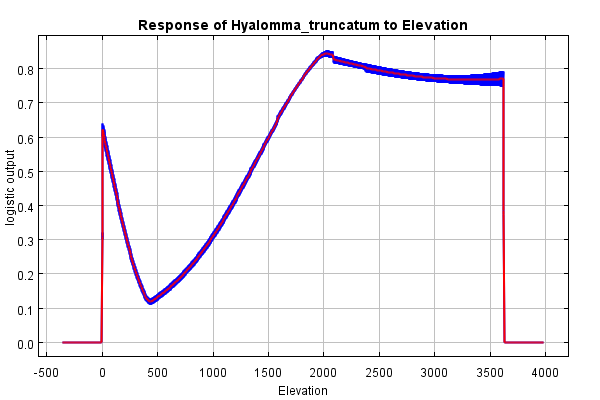

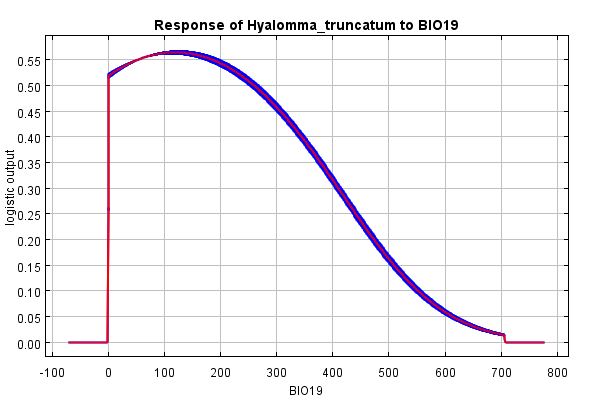

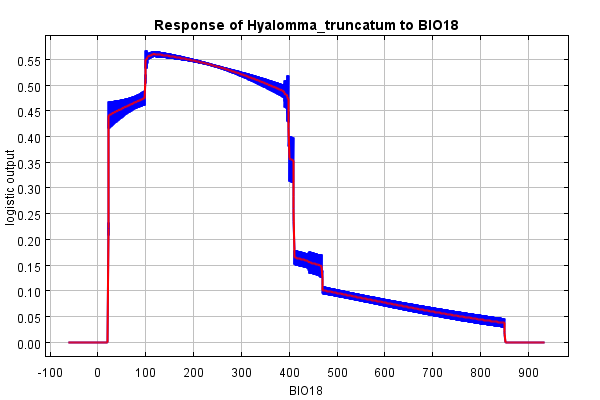

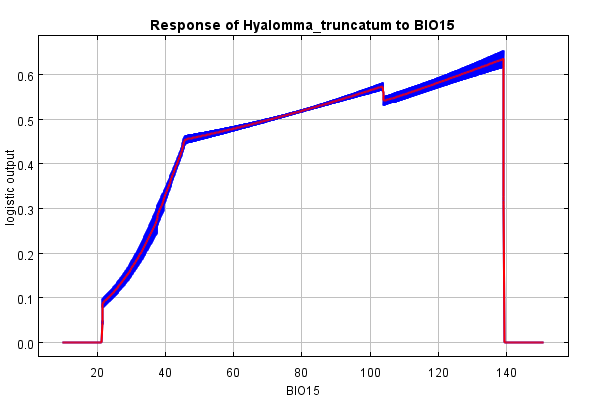

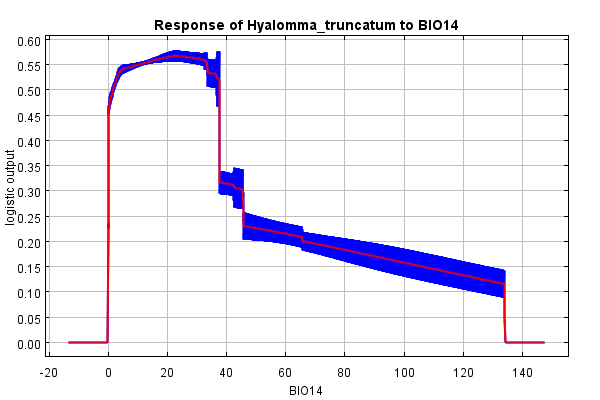

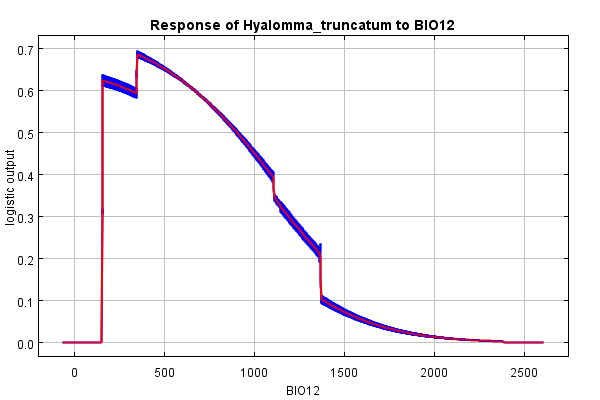

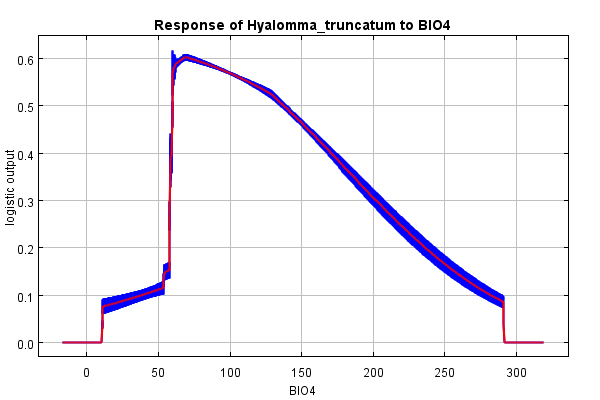


# **Fig. S19. Receiver Operating Characteristic (ROC) curve of the best MCP model for *Rhipicephalus appendiculatus***

The Maxent model for ***Rhipicephalus appendiculatus*** is represented by the Receiver Operating Characteristic (ROC) curve. This curve is an average of the results obtained from 25 replicate runs. The specificity is determined based on the predicted area.


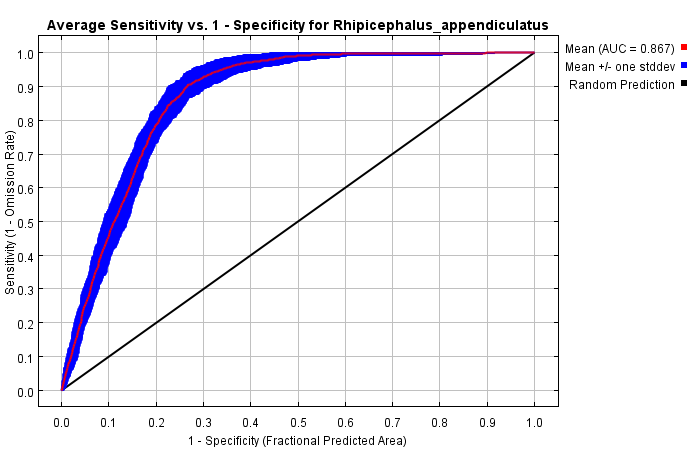


# **Table S7. Relative contributions of environmental and meteorological variables to the MaxEnt model**

| Variable | Percent contribution | Permutation importance |
| --- | --- | --- |
| Elevation | 36 | 10.2 |
| bio11 | 34 | 33.1 |
| Percent Tree Cover | 8.1 | 3.8 |
| bio14 | 5.3 | 15 |
| bio19 | 4.5 | 12.5 |
| bio7 | 4 | 9 |
| bio15 | 2.7 | 10.2 |
| bio18 | 2.7 | 4.6 |
| Land Cover | 1.8 | 1 |
| bio13 | 0.8 | 0.6 |

# **Fig. S20. Jackknife plots of MaxEnt model for *Rhipicephalus appendiculatus* prediction.**

1. training gain plot; B. AUC; C. test gain plot


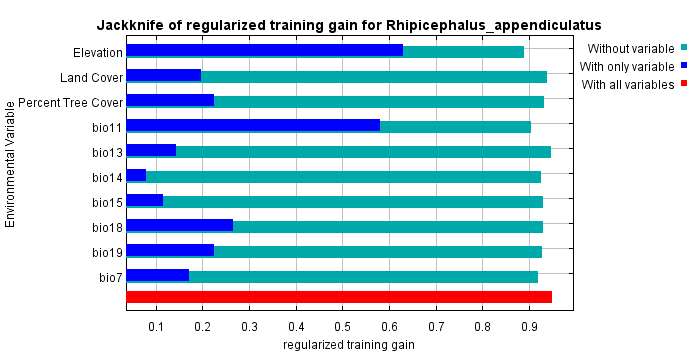

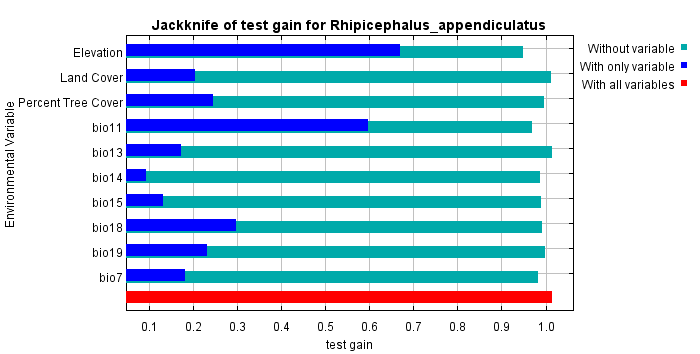

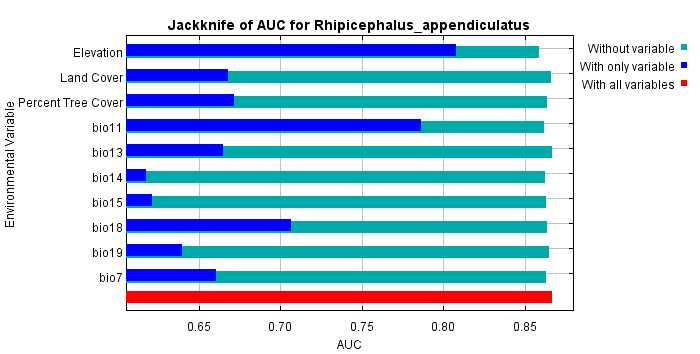


# **Fig. S21. Response curves of environmental variables indicate the likelihood of *Rhipicephalus appendiculatus* being present.**

These curves illustrate the average outcomes from 25 repeated Maxent model runs,

represented by the red line. The blue shaded areas show the standard deviation, highlighting the variability across these runs.


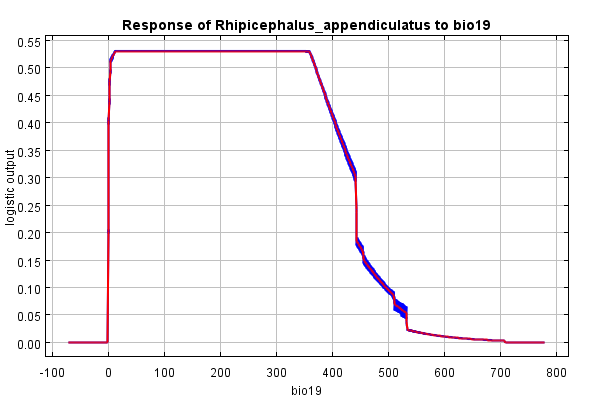

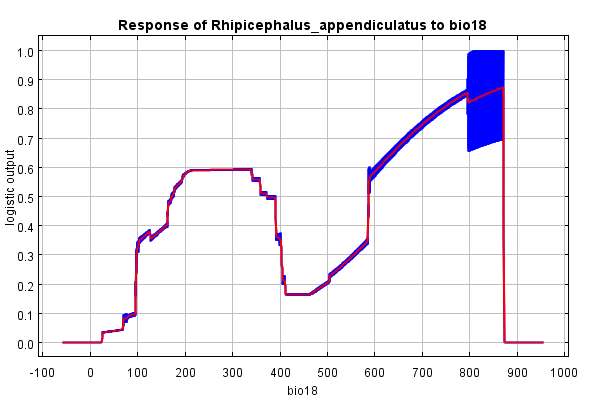

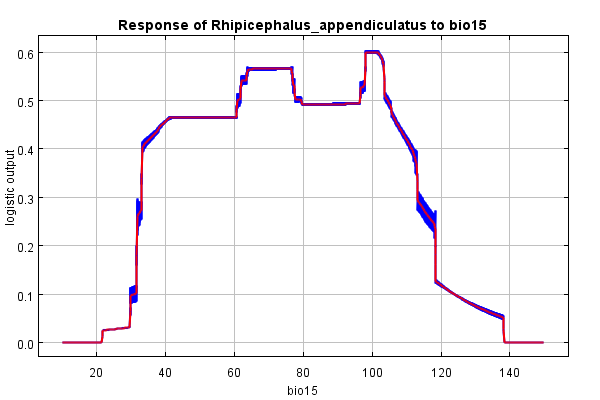

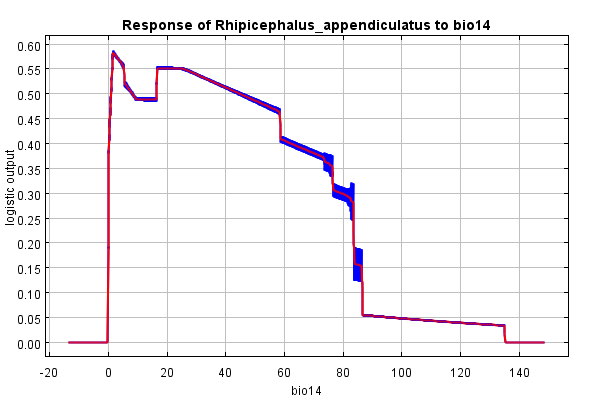

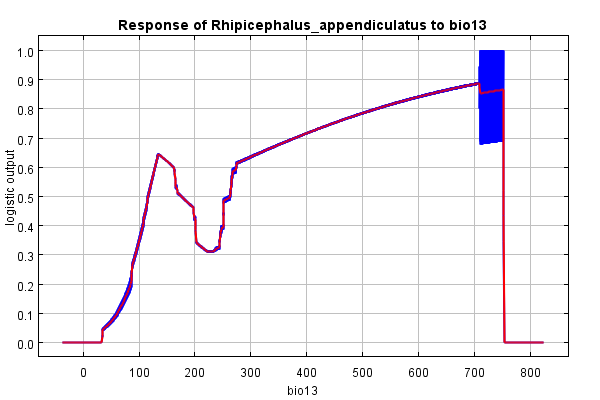

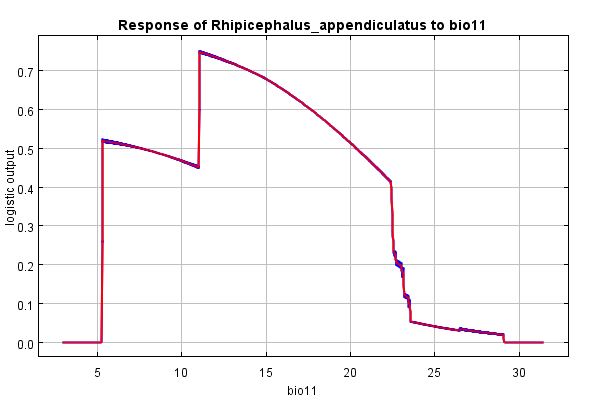

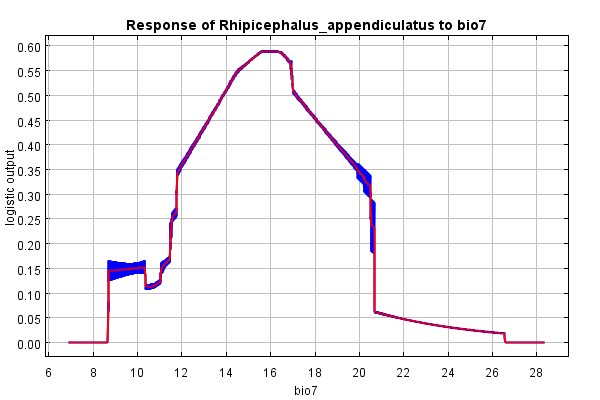

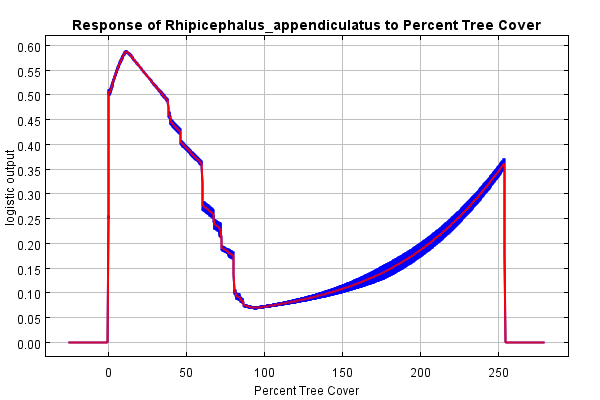

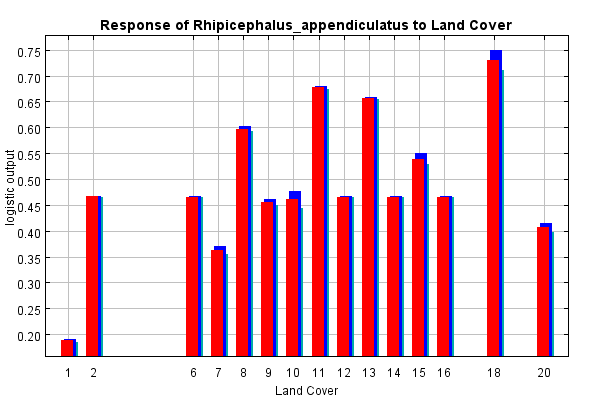

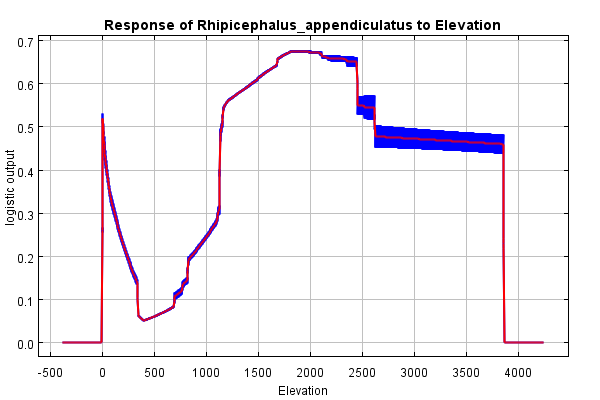

Supplement: Supplementary file 1 — Supplementary material 1. [file 40249_2025_1310_MOESM1_ESM.docx]
